# Antimicrobial Effect of Spices and Their Phytochemicals: A Novel Approach to Overcoming Antibiotic Resistance

**DOI:** 10.1002/mco2.70680

**Published:** 2026-03-22

**Authors:** Hettiyahandi Binodh De Silva, Yanqi Dai, Shervanthi Homer‐Vanniasinkam, Mohan Edirisinghe

**Affiliations:** ^1^ Department of Mechanical Engineering University College London London UK

**Keywords:** antimicrobial action, antibiotic resistance, bioactive, extraction processes, phytochemicals, spices

## Abstract

Antibiotic resistance is a significant global health challenge that demands innovative strategies to combat resistant pathogens. Spices, known for their culinary and medicinal qualities, have emerged as promising sources of antimicrobial agents due to their rich content of potent bioactive phytochemicals. Compounds such as flavonoids, phenolics, alkaloids, and terpenoids exhibit strong antibacterial, antifungal, and antiviral activities. These phytochemicals target microbial cell walls, membranes, and metabolic processes, effectively inhibiting pathogen growth and survival. Additionally, their ability to disrupt biofilms and synergize with conventional antibiotics enhances their potential to counter resistance mechanisms. This review examines the mechanisms and dissemination of antimicrobial resistance, the antimicrobial properties of spices and their phytochemicals, focusing on their modes of action, efficacy against multidrug‐resistant pathogens, specific extraction methods for each phytochemical, synergism with traditional antibiotics, safety and toxicological concerns, future research directions, and challenges in the widespread use of these spice‐derived compounds. It highlights the vast array of antimicrobial solutions derived from these spices and their natural phytochemicals, offering sustainable and effective means to address the escalating threat of antibiotic resistance.

## Graphical abstract

This graphical abstract shows the antibacterial potential of phytochemicals from 15 spices, showcasing key compounds, their extraction methods, and mechanisms of action. It highlights membrane disruption, ROS generation, enzyme inhibition, and biofilm prevention as core antibacterial pathways.

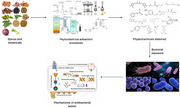

## 1 Introduction

The rise in antibiotic resistance is a growing global health crisis accelerated by the overuse and misuse of antibiotics in medical, agricultural, and industrial settings. As microbes are exposed to antibiotics, they rapidly evolve, developing mechanisms to survive even the most potent drugs, rendering once‐effective treatments obsolete. This phenomenon is exacerbated by improper prescription practices, self‐medication, and the extensive use of antibiotics in livestock to promote growth, which creates an environment that favors the selection of resistant microbial strains [1]. The diminishing effectiveness of antibiotics has spurred a need to explore alternative, sustainable solutions [2]. Among these, the antibacterial properties of spices present a promising avenue for exploration. Therefore, moving toward natural spice‐based antimicrobial therapies offers a novel approach to combating antibiotic resistance and emphasizes the importance of holistic, plant‐based medicines in maintaining and enhancing public health.

Spices are not just plant‐derived substances used primarily for flavoring, coloring, or preserving food in domestic and industrial applications. They are aromatic and contain bioactive compounds responsible for their characteristic taste and smell. Spices are dried seeds, fruits, roots, bark, or other vegetative substances that contain volatile oils, phenolics, alkaloids, or other compounds [3]. Their unique character, contributed by various vital components, makes them intriguing and exciting.

These exotic spices exhibit excellent antimicrobial properties, primarily due to bioactive phytochemicals that disrupt the cellular integrity of microbes. These compounds target the microbial cell membranes, causing alterations in permeability that lead to the leakage of cellular contents and ultimately result in cell death. Additionally, certain phytochemicals derived from spices can interfere with microbial enzyme activity and inhibit critical processes such as energy production, protein synthesis, and nucleic acid function, eventually leading to microbial cellular death. This multifaceted mechanism enables spices to act against a broad spectrum of bacteria, fungi, and other pathogens, making them valuable natural preservatives and potential alternatives to synthetic antimicrobials [4].

Therefore, this extensive review will closely analyze the constitution of the phytochemicals in some of the world's most commercially consumed spice varieties and examine their unique mechanisms responsible for enabling antimicrobial activity against a wide range of pathogenic species.

## 2 Mechanisms and Spread of Antimicrobial Resistance to Traditional Antibiotics

Antibiotics have transformed the field of medicine by making once ‐ lethal infections curable; yet, their effectiveness is increasingly undermined by the ability of bacteria to evolve resistance. Antibiotics exert their effects by targeting essential bacterial processes such as cell wall biosynthesis, protein synthesis, nucleic acid replication, and metabolic pathways. This resistance to antibiotics develops when bacterial species acquire genetic changes that neutralize the effects of antibiotics. These changes may occur spontaneously through point mutations in chromosomal genes or be acquired horizontally through plasmids, transposons, and bacteriophages [5]. The interplay between antibiotic activity and microbial resistance mechanisms explains not only how resistance emerges but also how it spreads rapidly across species and clinical settings.

First, β‐lactam antibiotics, such as cephalosporins, penicillins, and carbapenems, inhibit cell wall synthesis by binding to penicillin‐binding proteins (PBPs), which are transpeptidases responsible for cross‐linking peptidoglycan strands by mimicking the d‐Ala‐d‐Ala motif of the natural substrate. β‐Lactams form covalent complexes with PBPs, preventing peptidoglycan cross‐linking and leading to bacterial cell lysis [6]. Resistance to these antibiotics mainly arises in two ways. First, β‐lactamase enzymes hydrolyze the β‐lactam ring, releasing an inactive open‐ring product. Different classes of β‐lactamases have distinct active‐site chemistries, such as class A, C, and D enzymes, which utilize a serine‐based mechanism. In contrast, class B metallo‐β‐lactamases employ zinc ions to catalyze hydrolysis [7]. The clinical impact is significant, as carbapenemases, such as *Klebsiella pneumoniae* carbapenemase (KPC), New Delhi metallo ‐ β ‐lactamase (NDM), Verona integron ‐ encoded metallo ‐ β ‐ lactamase (VIM), and Oxacillinase ‐ 48 (OXA‐48)‐like variants, can inactivate carbapenems, often leaving few treatment options [8]. Second, resistance can develop through alteration of PBPs themselves [9]. In *Staphylococcus aureus*, the *mecA* gene encodes PBP2a, an alternative transpeptidase with a very low affinity for β‐lactams, meaning that peptidoglycan cross‐linking continues even at high drug concentrations [10]. Similarly, in *Streptococcus pneumoniae*, mosaic PBPs encoded by recombined gene sequences reduce penicillin affinity, underpinning penicillin‐resistant pneumococcal disease [11]. Glycopeptides, such as vancomycin, also target cell wall synthesis but act differently by binding directly to the d‐Ala‐d‐Ala termini of peptidoglycan precursors, thereby sterically blocking transglycosylation and transpeptidation [12]. Resistance occurs through operons such as *vanA* and *vanB*, which encode enzymes that remodel the precursor from d‐Ala‐d‐Ala to d‐Ala‐d‐Lac. This substitution removes a critical hydrogen bond, reducing vancomycin's binding affinity by around 1000‐fold, and enzymes such as VanH (a dehydrogenase producing d‐lactate) and VanA (a ligase attaching d‐Lac to d‐Ala) work together to reprogramme the biosynthetic pathway [13]. Meanwhile, VanX removes residual d‐Ala‐d‐Ala, ensuring full resistance [14]. This precise enzymatic activity demonstrates that resistance can develop not only through gene acquisition but also through reorganization of core metabolic pathways.

Second, protein synthesis inhibitors constitute another major category. Aminoglycosides such as tobramycin and gentamicin irreversibly bind to the 30S ribosomal subunit, causing codon misreading and disrupting protein synthesis [15]. Resistance mainly arises through aminoglycoside‐modifying enzymes. Acetyltransferases (AAC) transfer acetyl groups, phosphotransferases (APH) add phosphate groups, and nucleotidyltransferases (ANT) attach adenyl groups to specific hydroxyl or amino sites on the aminoglycoside molecule, preventing their binding to ribosomes [16]. More recently, plasmid‐borne 16 S rRNA methyltransferases, such as ArmA, methylate nucleotides within the aminoglycoside binding site itself, blocking drug interaction with the ribosome and conferring broad‐spectrum aminoglycoside resistance [17]. Tetracyclines, which reversibly bind to the 30S subunit and inhibit aminoacyl‐tRNA binding to the A‐site, are resisted by efflux pumps and ribosomal protection proteins. Efflux pumps, encoded by *tetA* or *tetB*, utilize the proton motive force to expel tetracyclines. Ribosomal protection proteins, such as Tet(M) and Tet(O), are GTPases that mimic elongation factors, dislodging tetracycline from the ribosome and thereby restoring protein synthesis [18]. Macrolides, lincosamides, and streptogramin B antibiotics bind to the 50S ribosomal subunit at the peptidyl transferase center, obstructing elongation [19]. Resistance is generally mediated by *erm* methyltransferases, which methylate adenine at position A2058 of the 23S rRNA, altering the conformation of the binding site. The *erm* genes are frequently carried on mobile genetic elements and can be induced by sub‐inhibitory antibiotic exposure, rendering resistance widespread and difficult to control [20]. Linezolid, an oxazolidinone, also binds at the 23S rRNA site [21]. Still, resistance develops via the plasmid‐borne *cfr* gene, which methylates A2503 of the 23S rRNA, conferring cross‐resistance to multiple ribosome‐targeting drugs [22]. Point mutations in ribosomal proteins L3 and L4 can also reduce drug affinity [23].

Similarly, both target modification and protective proteins confer resistance to drugs that target nucleic acid synthesis. Fluoroquinolones inhibit DNA gyrase and topoisomerase IV by stabilizing the DNA enzyme cleavage complex, causing lethal double‐stranded breaks [24]. Resistance arises through mutations in the quinolone resistance‐determining regions of the *gyrA*, *gyrB*, *parC*, and *parE* genes, which diminish the binding of fluoroquinolones. Plasmid‐mediated resistance involves Qnr proteins, which are pentapeptide‐repeat proteins that physically bind to topoisomerase IV and DNA gyrase, thereby shielding them from the action of quinolones. Rifamycins, such as rifampicin, inhibit RNA synthesis by binding to the β‐subunit of RNA polymerase [25]. However, in *Mycobacterium tuberculosis*, mutations in the *rpoB* gene alter this subunit, preventing binding [26].

In addition to drug inactivation and target modification, bacteria can resist antibiotics by limiting their entry. Gram‐negative bacteria achieve this by altering porin proteins in the outer membrane, reducing permeability. For instance, *Pseudomonas aeruginosa* often mutates or downregulates the OprD porin, thereby decreasing imipenem uptake [27], while *Enterobacter* and *Klebsiella* spp. downregulate OmpF and OmpC, which reduces cephalosporin penetration [28]. Some antibiotics, such as fosfomycin, require specific transporters for entry, and mutations in GlpT or UhpT transporters can completely prevent entry [29].

Additionally, efflux pumps actively export antibiotics, thereby lowering intracellular concentrations. These pumps may belong to different superfamilies, including the major facilitator superfamily, the resistance–nodulation–division (RND) and the adenosine triphosphate (ATP)‐binding cassette transporters [30]. In *E. coli*, the AcrAB–TolC system, a tripartite RND pump, spans the periplasm, inner membrane and outer membrane, utilizing the proton motive force to expel fluoroquinolones, tetracyclines, chloramphenicol, and β‐lactams [31]. *P.aeruginosa* possesses multiple efflux systems, including MexAB–OprM, which extrudes a wide range of drugs [32]. Overexpression of these pumps can transform low‐level resistance into clinically significant multidrug resistance.

Another strategy is metabolic bypass, where bacteria acquire alternative enzymes that bypass the blocked pathway. Sulfonamides inhibit dihydropteroate synthase (DHPS) in folate synthesis, but plasmid‐borne *sul* genes encode resistant DHPS variants with reduced affinity [33]. Trimethoprim inhibits dihydrofolate reductase (DHFR), yet resistance is conferred by *dfr* genes that encode alternative DHFR enzymes [34]. These genes are often embedded in integrons that also carry resistance to other drug classes, facilitating coselection [35].

Cell envelope modification also contributes to resistance, especially against last‐resort drugs. Colistin, a polymyxin, targets the negatively charged lipid A component of lipopolysaccharides (LPS) in the outer membranes of Gram‐negative bacteria, disrupting membrane integrity [36]. The plasmid‐mediated *mcr* genes encode phosphoethanolamine transferases, which modify lipid A by attaching phosphoethanolamine, thereby reducing colistin binding affinity [37]. Since the discovery of *mcr‐1* in 2015, numerous *mcr* variants have been identified worldwide [38]. Chromosomal mutations, such as *mgrB* inactivation in *Klebsiella pneumoniae*, also alter lipid A modification pathways, conferring resistance to colistin [39].

Finally, bacteria within biofilms demonstrate a collective resistance phenotype. Biofilms are structured communities enclosed in an extracellular polymeric matrix made up of polysaccharides, proteins, and extracellular DNA. This matrix limits antibiotic penetration and forms microenvironments with gradients of nutrients, oxygen, and pH [40]. Inside biofilms, many cells enter a slow‐growing or dormant “persister” state, which is naturally resistant to antibiotics that target active processes [41]. Biofilm‐associated infections, such as those caused by *Pseudomonas aeruginosa* in cystic fibrosis lungs [42] or *Staphylococcus epidermidis* on indwelling devices, are therefore hard to eradicate, often necessitating device removal or long‐term suppressive therapy [43].

This developing antibiotic resistance spreads through several distinct modes, each contributing to the rapid dissemination of resistant traits in bacterial populations. Vertical transmission occurs when resistance arises through chromosomal mutations and is inherited by daughter cells during replication [44]. For instance, mutations in the rpoB gene of *Mycobacterium tuberculosi*s are passed clonally, conferring rifampicin resistance [45]. More dynamic, however, is horizontal gene transfer, which operates through three primary routes. First, conjugation involves the transfer of plasmids via pilus‐mediated contact between bacteria; this is the most clinically significant mode and is exemplified by the spread of blaNDM‐1 carbapenemase and mcr‐1 colistin resistance genes among *Enterobacterales* [46]. Similarly, transformation occurs when bacteria take up naked DNA from the environment, as seen in Streptococcus pneumoniae, which integrates foreign PBP genes from commensal streptococci, leading to penicillin resistance [47]. Additionally, transduction, mediated by bacteriophages, enables the packaging of resistance genes into phage particles and their transfer between bacteria. In staphylococci, phages have been shown to mobilize genes, including mecA, which confers methicillin resistance [48]. Finally, mobile genetic elements such as integrons and transposons facilitate the clustering and movement of resistance genes across different platforms. Class 1 integrons often carry sul and dfr genes, providing resistance to sulfonamides and trimethoprim, alongside other determinants [49].

Together, these mechanisms create a powerful genetic toolkit that enables resistance to spread within species, across genera, and even between environmental and clinical settings.

Therefore, as the global crisis of antimicrobial resistance continues to worsen, there is an urgent need to explore innovative strategies beyond the traditional development of new antibiotics. Conventional drugs are increasingly losing their effectiveness, and the pipeline for new agents remains limited. The global scale of damage from antimicrobial resistance is clearly visible through the Global Antimicrobial Resistance and Use Surveillance System reports and the World Health Organisation reports, which have reported 1.27 million deaths directly attributable to drug‐resistant bacterial infections and an additional 4.95 million deaths were estimated to be associated with bacterial antibiotic resistance [44] in 2019 [50].

## 3 Analysis of Spices and their Unique Antimicrobial Phytochemicals

In this context, researchers are turning to natural sources for potential solutions, with particular interest in plant‐derived compounds that may either complement existing antibiotics or serve as alternatives. Among these, spice‐derived phytochemicals have attracted increasing attention for their diverse biological activities and potential to enhance antimicrobial therapy through novel mechanisms, which will be examined in detail below.

As shown in Table 1, spices contain a variety of phytochemicals that have diverse functions. Each spice will be analyzed individually below, focusing on the potential of spice‐based antibacterial therapies. Table 2 presents a comprehensive overview of 14 major spices, their main phytochemicals, and their chemical structures. The associated letters provide a unique reference code for use in describing them in this review.

**TABLE 1:** Major global spices and botanicals defined by phytochemical profile, origin, organoleptic traits, extraction methods, form, and commercial use.

Spice	Phytochemicals	Main antibacterial phytochemical	Geographic location	Organoleptic classification	Extraction processes	Physical form	Main uses
Turmeric [51]	Curcuminoids, turmerones, sesquiterpenes	Curcumin	South Asia, India	Earthy, bitter, pungent	Solvent extraction, supercritical CO_2_	Powder, dried rhizome	Anti‐inflammatory, digestive health, coloring agent
Garlic [52]	Allicin, sulfur compounds, flavonoids	Allicin	Central Asia, Mediterranean	Pungent, spicy, sulfurous	Steam distillation, cold pressing	Fresh bulb, powder, oil	Immunity boost, cardiovascular health, flavoring
Cinnamon [53]	Cinnamaldehyde, eugenol, coumarin, linalool	Cinnamaldehyde	South Asia, Sri Lanka	Sweet, warm, woody	Steam distillation, solvent extraction	Bark, powder, oil	Flavoring, antimicrobial, antidiabetic
Clove [54]	Eugenol, beta‐caryophyllene, tannins	Eugenol	Indonesia, Madagascar	Spicy, strong, sweet	Steam distillation, hydrodistillation	Whole buds, powder, oil	Pain relief (dental), flavoring, antimicrobial
Ginger [55]	Gingerol, shogaol, zingerone	Gingerol	South Asia, India	Spicy, pungent, warming	Steam distillation, solvent extraction	Fresh root, dried, powder	Nausea relief, digestion aid, anti‐inflammatory
Black pepper [56]	Piperine, volatile oils, pipene	Piperine	South India, Southeast Asia	Spicy, pungent, sharp	Steam distillation, supercritical CO_2_	Whole peppercorn, powder	Digestive aid, flavoring, stimulant
Cumin [57]	Cuminaldehyde, terpenes, flavonoids	Cuminaldehyde	Middle East, Mediterranean	Earthy, warm, bitter	Steam distillation, solvent extraction	Whole seeds, powder	Flavoring, digestive health, anti‐inflammatory
Cardamom [58]	Cineole, alpha‐terpineol, linalool	Cineole	South India, Guatemala	Sweet, warm, slightly minty	Steam distillation, supercritical CO_2_	Whole pods, powder	Digestive aid, flavoring, breath freshener
Bayleaf [59]	Cineole, linalool, myrcene, sabinene	Cineole	Southeast Asia (Indonesia)	Herbal, slightly floral, bitter	Steam distillation, solvent extraction	Whole dried leaves	Flavoring, anti‐inflammatory, stress relief
Nutmeg [60]	Myristicin, elemicin, eugenol	Myristicin	India, Mediterranean	Sweet, warm, slightly nutty	Steam distillation, solvent extraction	Whole seeds, powder	Flavoring, digestive health, sedative
Basil [61]	Linalool, eugenol, methyl cinnamate	Eugenol	India, Mediterranean	Fresh, sweet, peppery	Steam distillation, hydrodistillation	Fresh leaves, dried, oil	Flavoring, stress relief, antimicrobial
Mustard [62]	Sinigrin, isothiocyanates, allyl isothiocyanate	Allyl isothiocyanate	Mediterranean, Asia	Pungent, sharp, bitter	Steam distillation, cold pressing	Seeds, powder, oil	Flavoring, stimulant, anti‐inflammatory
Fenugreek [63]	Saponins, alkaloids, flavonoids, coumarins	Saponins	Mediterranean, South Asia	Bitter, slightly sweet	Solvent extraction, hydrollization	Whole seeds, powder	Lactation aid, digestive health, antidiabetic
Lemongrass [64]	Citral, limonene, myrcene, geraniol	Citral	South Asia, India	Citrusy, fresh, lemon‐like	Steam distillation, solvent extraction	Fresh, dried, oil	Flavoring, anti‐inflammatory, stress relief
Oregano [65]	Carvacrol, thymol, rosmarinic acid, terpenes	Carvacrol	Mediterranean, South Europe	Pungent, herbal, slightly bitter	Steam distillation, solvent extraction, Supercritical CO_2_	Dried leaves, powder, oil	Flavoring, antimicrobial, antioxidant, digestive aid

**TABLE 2:** Main phytochemicals and their chemical structures of different spices.

Spice	Phytochemical name	Phytochemical structure	Associated letter
Turmeric [66]	Curcumin	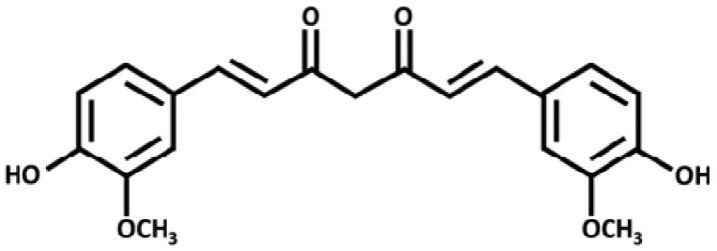	1a.
	Turmerones	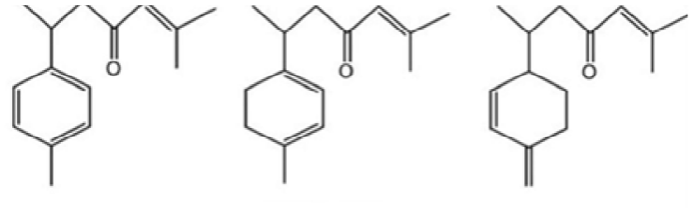	1b.
	1,8‐Cineole	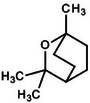	1c.
	Germacrone	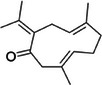	1d.
	Zingiberene	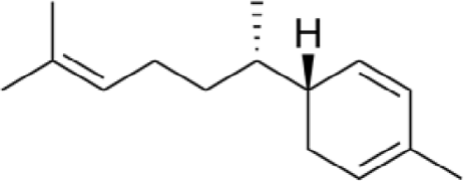	1e.
Garlic [67]	Allicin	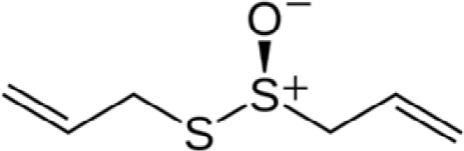	2a.
	Diallyl disulfide (DADS)	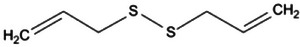	2b.
	Diallyl trisulfide (DATS)	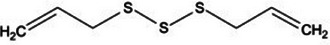	2c.
	Ajoene	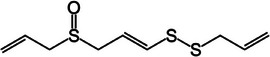	2d.
	S‐allyl cysteine	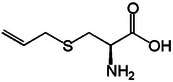	2e.
	Allixin	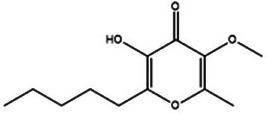	2f.
	Quercetin	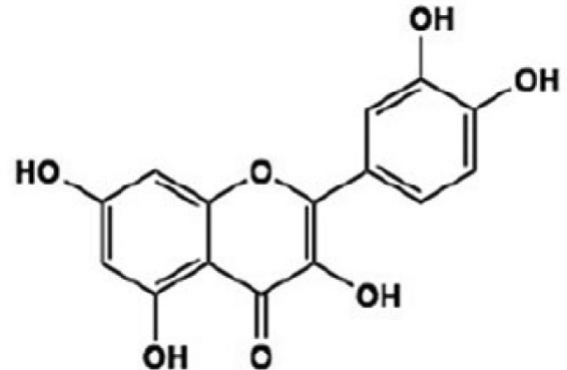	2g.
	Kaempferol	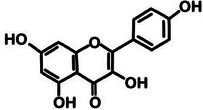	2h.
Cinnamon [68]	Cinnamaldehyde	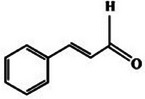	3a.
	Eugenol	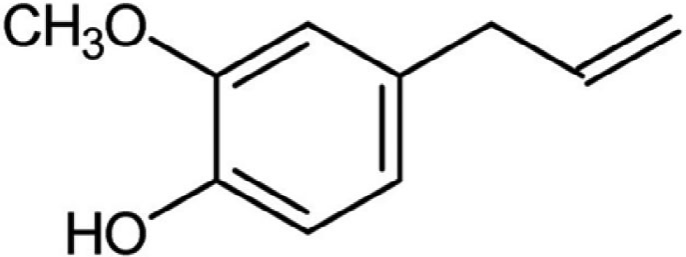	3b.
	Cinnamic acid	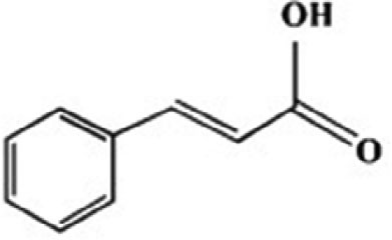	3c.
	Procyanidins	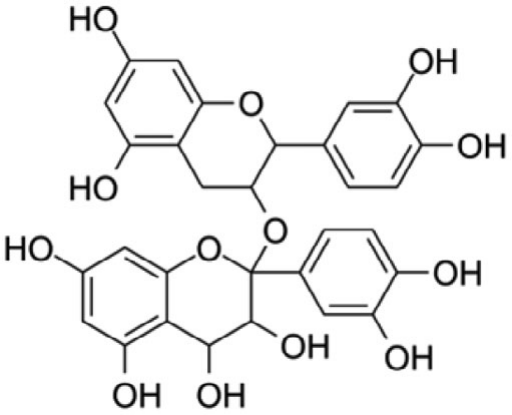	3d.
	Coumarin	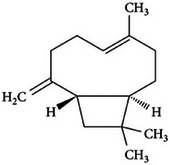	3e.
	Catechin	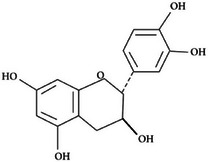	3f.
Clove [68]	Beta‐caryophyllene	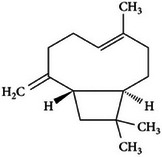	4a.
	Tannins	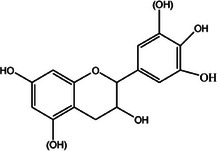	4b.
	Gallic acid	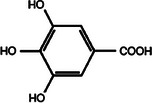	4c.
	Oleanolic acid	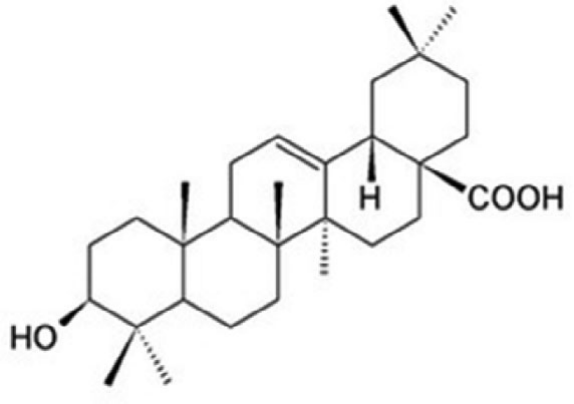	4d.
Ginger [69]	[6]‐Gingerol	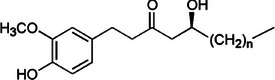	5a.
	Shogaols	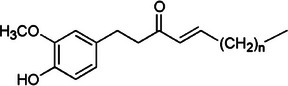	5b.
	Zingerone	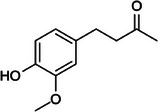	5c.
	Paradol	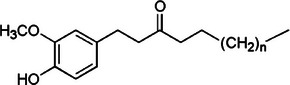	5d.
Black pepper [70]	Piperine	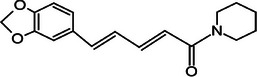	6a.
Cumin [71]	Cuminaldehyde	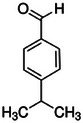	7a.
	P‐cymene	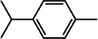	7b.
	Limonene	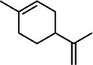	7c.
Cardamom [72]	α‐Terpineol	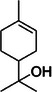	8a.
	Terpinyl acetate	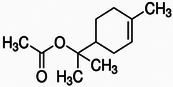	8b.
	Sabinene	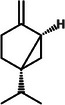	8c.
	Borneol	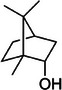	8d.
Bay leaf [73]	Linalool	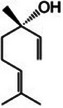	9a.
Nutmeg [74]	Myristicin	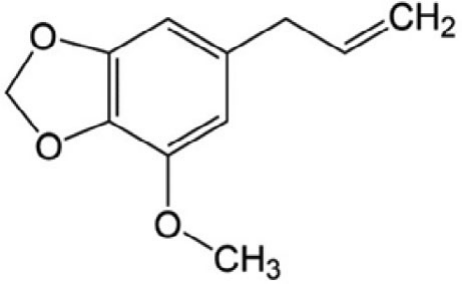	10a.
	Safrole	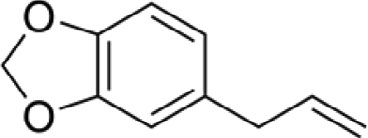	10b.
Basil [75]	Methyl chavicol	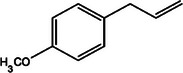	11a.
	Camphor	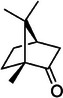	11b.
Mustard [76]	Sinigrin	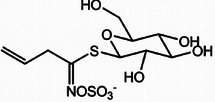	12a.
	AITC	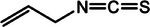	12b.
	Phenethyl isothiocyanate (PEITC)	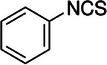	12c.
	Erucic acid	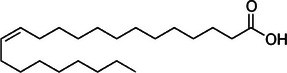	12d.
Fenugreek [77]	Diosgenin	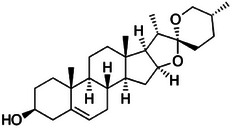	13a.
	Trigonelline	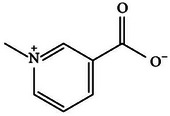	13b.
	Galactomannan	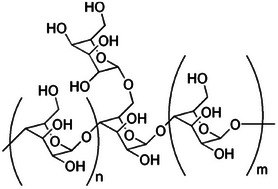	13c.
	Vitexin	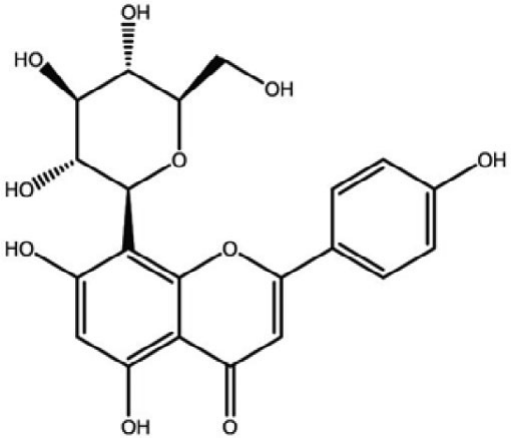	13d.
	Isovitexin	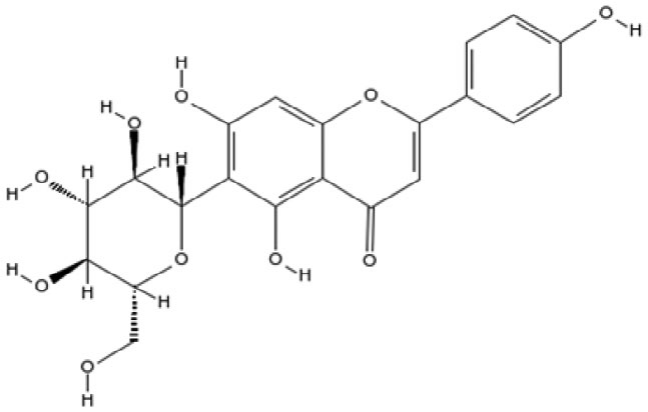	13e.
Lemongrass [65]	Geraniol	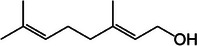	14a.
	Cymbopogonol	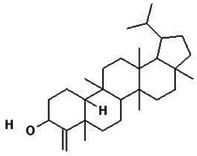	14b.
	Terpinolene	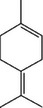	14c.
	Elemicin	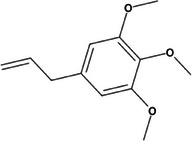	14d.
Oregano [65]	Carvacrol	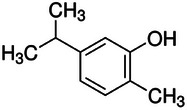	15a.
	Thymol	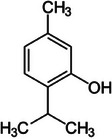	15b.
	Rosmarinic acid	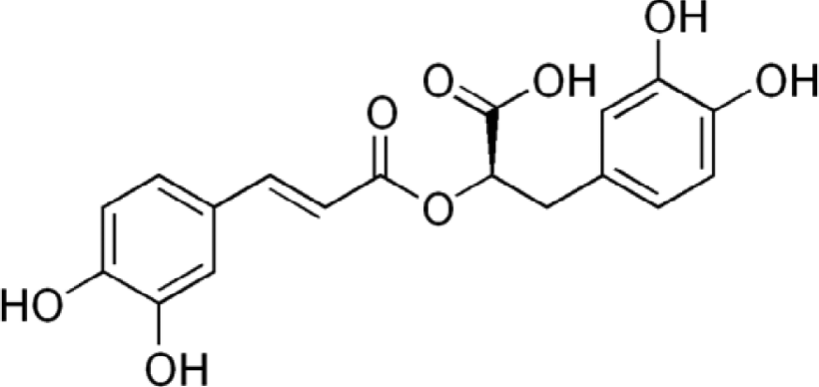	15c.
	Ursolic acid	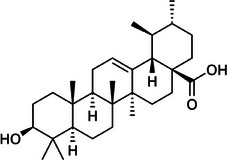	15d.

### 3.1 Turmeric

Turmeric (*Curcuma longa*) is rich in bioactive phytochemicals, most notably curcuminoids and essential oils, which exhibit potent antimicrobial properties [78]. These compounds target microbial cell membranes, enzymes, and vital metabolic processes, making turmeric an effective natural antimicrobial agent. Below is an advanced description of the primary phytochemicals in turmeric and their mechanisms of antimicrobial action.

First, curcumin, the most prominent curcuminoid in turmeric, is a polyphenol known for its potent antimicrobial and antioxidant properties. Structurally, curcumin (C_21_H_20_O_6_), as observed in Table 2:1a, is a diarylheptanoid characterized by two aromatic phenyl rings connected by a seven‐carbon chain with conjugated double bonds. Each phenyl ring is substituted with hydroxyl (‐OH) and methoxy (‐OCH_3_) groups at the fourth and fifth positions, which enhances its electrophilic reactivity [79]. This unique structure allows curcumin to interact effectively with microbial membranes and proteins. Curcumin and its derivatives exert antimicrobial activity through a multitude of mechanisms, primarily disrupting microbial membranes and inhibiting enzyme functions. Curcumin's lipophilic nature enables integration into microbial lipid bilayers, destabilizing the cell membrane and increasing permeability. This leads to the leakage of essential intracellular contents such as ATP, ions, and metabolites, ultimately resulting in microbial cell death. This membrane‐disruptive activity is particularly effective against gram‐positive bacteria such as *Staphylococcus aureus* and fungal pathogens such as *Candida albicans* [80]. Furthermore, curcumin inhibits microbial enzyme activity by interacting with thiol (‐SH) and amine groups within enzyme active sites, disrupting critical processes such as protein synthesis, DNA replication, and cell wall biosynthesis. Curcumin's ability to form hydrogen bonds with microbial nucleic acids further interferes with replication and transcription processes. By inhibiting ATP synthase, curcumin reduces energy production in microbial cells, severely impairing their survival and proliferation [81]. These multifaceted mechanisms make curcumin a highly effective antimicrobial agent.

Second, turmerones, the primary constituents of turmeric essential oil, are sesquiterpenoids (C_15_H_22_O), as observed in Table 2:1b, and are crucial to turmeric's antimicrobial action. Structurally, turmerones comprise a 15‐carbon backbone derived from isoprene units, featuring a characteristic furan ring fused to a hydrophobic carbon chain. Their structure includes varying hydroxyl (‐OH) or carbonyl (‐C═O) groups, influencing their reactivity and lipophilicity and enhancing their ability to interact with microbial membranes and enzymes [82]. Turmerones, particularly ar‐turmerone and α‐turmerone, exert their antimicrobial effects by integrating into microbial cell membranes, where their lipophilic nature causes membrane destabilization. This increases membrane permeability, leading to the leakage of essential intracellular contents such as ATP and ions, eventually causing microbial cell death. This membrane disruption is especially effective against gram‐negative bacteria, such as *Escherichia coli* and *Pseudomonas aeruginosa* [83], whose outer membrane is usually resistant to many antimicrobial agents. Additionally, beyond membrane disruption, turmerones are responsible for the inhibition of microbial enzymes responsible for protein and cell wall synthesis, which is brought about by binding to nucleophilic residues, such as serine or cysteine, and inactivating active sites of microbial enzymes, that halt key metabolic processes essential for microbial growth and replication [84]. This makes them particularly effective against fungal pathogens, such as *Aspergillus niger* and *Candida* species, where they disrupt both membrane integrity and essential enzyme systems. These multifaceted antimicrobial mechanisms make turmerones vital for turmeric's defense against various pathogens.

Additionally, 1,8‐cineole, as observed in Table 2:1c, is a monoterpene oxide found in turmeric essential oil, which plays a crucial role in its antimicrobial activity. Structurally, cineole (C_10_H_18_O) is a cyclic ether with a 10‐carbon backbone derived from two isoprene units, and the presence of the oxygen atom in the ether group enhances its overall reactivity [85]. This unique structure enables cineole to interact effectively with microbial proteins and cell membranes. Its primary mechanism of action involves disrupting microbial membranes by embedding itself within the lipid bilayer, altering membrane dynamics, and increasing permeability. This disruption causes the leakage of essential intracellular components such as ions and ATP, leading to cell death. In addition to membrane disruption, cineole inhibits microbial enzymes by modifying active sites, particularly those containing thiol (‐SH) groups, which are crucial for energy metabolism and cell wall biosynthesis. This dual mechanism of membrane disruption and enzyme inhibition makes 1,8‐cineole effective against a broad range of pathogens, including gram‐positive, gram‐negative bacteria and fungal pathogens.

Furthermore, germacrone, as observed in Table 2:1d, is another key sesquiterpene found in turmeric essential oil, which plays a significant role in its overall antimicrobial properties. Structurally, germacrone (C_15_H_22_O) is a bicyclic sesquiterpene with a 15‐carbon backbone featuring two fused rings and a carbonyl group (‐C═O) that enhances its reactivity [86]. This carbonyl group facilitates germacrone interactions with microbial proteins and enzymes, contributing to its antimicrobial action. Germacrone primarily disrupts microbial membranes through its lipophilic nature, enabling it to integrate into lipid bilayers and increase membrane permeability. This leads to the leakage of essential intracellular components, resulting in membrane destabilization and eventual cell death. Additionally, germacrone inhibits microbial enzymes involved in critical processes like DNA replication, protein synthesis, and cell wall biosynthesis by binding to active sites with nucleophilic groups. This enzyme inhibition disrupts vital metabolic pathways making germacrone particularly effective against gram‐positive bacterial and fungal pathogens, thus inhibiting microbial growth.

Finally, shown in Table 2:1e, zingiberene is a sesquiterpene found in turmeric's essential oil and is crucial in its antimicrobial and anti‐inflammatory effects. Structurally, zingiberene (C_15_H_24_) is a bicyclic sesquiterpene equipped with a highly hydrophobic nature, facilitating efficient integration into lipid‐rich environments such as microbial membranes [86]. By embedding into the lipid bilayer, zingiberene disrupts membrane integrity and increases permeability, leading to the leakage of essential intracellular contents and ultimately causing microbial cell death. Additionally, zingiberene enhances the effects of other antimicrobial compounds in turmeric by facilitating their penetration into microbial cells and targeting intracellular components. Its effectiveness is particularly noted against gram‐negative bacteria and fungal pathogens, where it destabilizes protective outer barriers such as the cell wall and outer membranes, resulting in enhanced antibacterial action.

### 3.2 Garlic

Garlic (*Allium sativum*) is well known for its potent antimicrobial properties, attributed to a complex array of bioactive phytochemicals, including organosulfur compounds, flavonoids, and phenolic compounds. These compounds exhibit intense antibacterial, antiviral and antifungal action by targeting microbial membranes, enzymes, and other metabolic pathways.

First, allicin (C_6_H_10_OS_2_) is a highly reactive sulfur‐containing compound produced when garlic is crushed or chopped, occurring when the enzyme alliinase converts the precursor alliin into allicin. Structurally, Table 2:2a shows that allicin is characterized by its thiosulfinate group (‐S(O)S‐), which is responsible for its potent reactivity and biological activity [87]. This unique sulfur‐based structure enables allicin to engage in potent interactions with microbial enzymes, proteins, and cell membranes, contributing to its broad‐spectrum antimicrobial properties [88]. The thiosulfinate group in allicin is particularly reactive toward thiol groups found in cysteine residues, and forming disulfide bonds with these cysteine residues disrupts the functioning of critical enzymes, thus interfering with vital metabolic processes. One of the primary targets of allicin is the microbial enzymes involved in cell wall biosynthesis, energy metabolism, and protein synthesis, occurring through the binding of allicin to cysteine residues in enzyme active sites, which blocks overall microbial catalytic function, leading to the disruption of key cellular processes [89]. This is observed through allicin's inhibition of enzymes essential for peptidoglycan synthesis, which weakens bacterial cell walls. This mode of antibacterial action is particularly detrimental against gram‐positive bacteria that rely heavily on robust peptidoglycan layers for structural integrity. In terms of energy metabolism, allicin affects enzymes involved in ATP production, reducing the energy available for microbial growth and survival. Allicin also disrupts protein synthesis by interfering with enzymes involved in amino acid metabolism and translation, essential for the synthesis of new proteins for microbial development and replication. Additionally, allicin has been shown to affect DNA replication by disrupting enzymes responsible for the replication process, further inhibiting microbial proliferation. Allicin binds to these enzymes and impairs the cell's replication machinery, slowing down or stopping microbial growth. Furthermore, allicin's lipophilic properties facilitate effective penetration into microbial cell membranes, destabilizing overall cellular structural integrity. This membrane disruption is especially effective against gram‐positive bacterial and fungal pathogens, which are more susceptible to destabilized or weakened lipid bilayers [89]. Once embedded in the microbial membrane, allicin increases membrane permeability, leading to the leakage of critical intracellular components such as ions, ATP, and metabolites, disrupting overall cellular osmotic balance and leading to cell death [88]. Therefore, the combination of enzyme inhibition, disruption of DNA replication, and membrane destabilization makes allicin a potent antimicrobial agent and an effective natural antimicrobial compound in garlic.

Second, diallyl disulfide (DADS; C_6_H_10_S_2_) and diallyl trisulfide (DATS; C_6_H_10_S_3_) are organosulfur compounds derived from the breakdown of allicin and, as observed in Table 2:2b,2c, are characterized by their disulfide (S‐S) and trisulfide (S‐S‐S) linkages [88]. DADS and DATS exert their antimicrobial effects through membrane disruption and enzyme inhibition. These sulfur–sulfur bonds enable them to react with thiol groups in microbial proteins, mainly targeting enzymes critical for energy metabolism, such as ATP synthase, and those involved in DNA replication and protein synthesis. By interacting with these enzymes, DADS and DATS inactivate vital metabolic pathways, impairing microbial growth and reproduction. Additionally, their lipophilic nature facilitates integration into microbial lipid bilayers, destabilizing membrane integrity and increasing permeability, which leads to the leakage of intracellular contents and loss of cell viability. This dual action makes DADS and DATS especially effective against gram‐positive bacteria such as *Staphylococcus aureus* and gram‐negative bacteria such as *Escherichia coli* [90], weakening microbial defenses on multiple fronts.

Moreover, ajoene (C_9_H_14_OS_3_) is a highly reactive organosulfur compound that forms during the decomposition of allicin and, as observed in Table 2:2d, is characterized by a vinyl disulfide group (‐S‐S‐) and a sulfoxide group. Its potent antimicrobial activity is driven by its dual ability to disrupt microbial cell membranes and inhibit key microbial enzymes [88]. The disulfide bond in ajoene reacts with thiol groups in microbial proteins, leading to protein denaturation and enzyme inactivation. Ajoene targets enzymes involved in critical processes such as cell wall biosynthesis, DNA replication, and protein synthesis, effectively halting microbial growth and replication. Through the integration into microbial membranes, particularly those of fungal pathogens such as *Candida albicans* or gram‐positive bacteria such as *Bacillus subtilis* [91], ajoene increases membrane permeability, resulting in leakage of intracellular components and eventual cell death [92]. Additionally, ajoene inhibits biofilm formation, making it especially effective against potent biofilm‐associated infections caused by pathogens such as *Staphylococcus aureus* and *Pseudomonas aeruginosa* [91], which are a leading cause for infection due to their resistance toward antibiotics and other traditional modes of treatment.

Additionally, s‐allyl cysteine (SAC; C_6_H_11_NO_2_S) is a water‐soluble organosulfur compound found in garlic, which is derived from cysteine with an allyl group attached to the sulfur atom as observed in Table 2:2e, SAC primarily involves antimicrobial action by inhibiting microbial enzymes critical for DNA replication and protein synthesis. SAC's thiol group facilitates interactions with cysteine residues in microbial proteins, which inactivates enzymes essential for microbial growth and reproduction. Additionally, SAC exhibits membrane‐disruptive properties, particularly against gram‐positive bacteria, where it destabilizes the lipid bilayer, increasing membrane permeability and resulting in the leakage of intracellular components [93]. Beyond its direct antimicrobial action, SAC also possesses antioxidant properties, neutralizing reactive oxygen species (ROS) within microbial cells. This reduction in oxidative stress further weakens the microbes’ ability to survive, amplifying SAC's overall antimicrobial effectiveness.

Furthermore, allixin (C_9_H_10_O_3_) is a dihydrofuran compound derived from garlic, and as observed in Table 2:2f, it contains a furan ring and a benzene ring, along with hydroxyl and methoxy groups, which enhances overall reactivity. Allixin exhibits broad‐spectrum antimicrobial activity by disrupting microbial membranes and inhibiting key enzymes. Allixin's lipophilic nature enables integration into microbial lipid bilayers, destabilizing the membrane, increasing membrane permeability, and causing the leakage of vital intracellular components. Additionally, allixin inhibits the production of enzymes critical for DNA replication, cell wall biosynthesis, and protein synthesis by interacting and disrupting active sites and impairs microbial development and reproduction. This dual antibacterial action mechanism of enzyme inhibition and membrane disruption makes allixin particularly effective against various pathogens, including fungal species such as *Aspergillus niger* and bacterial species such as *Salmonella enteric* [94].

Moreover, quercetin (C_15_H_10_O_7_) is a flavonoid found in garlic and, as observed in Table 2:2g, contains a flavone backbone with multiple hydroxyl groups (‐OH) attached to its benzene rings, which contributes to its potent antioxidant and antimicrobial properties. Quercetin increases antimicrobial activity by inhibiting microbial DNA gyrase, an enzyme essential for bacterial DNA replication. The hydroxyl groups enable quercetin to interact with microbial DNA, disrupting replication and transcription processes and thus inhibiting microbial growth. Additionally, quercetin acts as a potent antioxidant by neutralizing ROS, which reduces oxidative stress within microbial cells and indirectly impairs their survival in hostile environments. This combination of direct enzyme inhibition and oxidative stress reduction improves the antibacterial versatility of quercetin. It makes it effective against fungal pathogens such as *Candida albicans* and gram‐positive bacterial species such as *Staphylococcus aureus* [46].

Finally, kaempferol (C_15_H_10_O_6_) is a flavonoid similar in structure to quercetin and, as seen in Table 2:2h, contains a flavone backbone with hydroxyl groups that enhance its reactivity. Kaempferol inhibits microbial growth through the disruption to cell wall biosynthesis and interference with DNA replication. Kaempferol inhibits and disrupts microbial replication and transcription processes by forming hydrogen bonds with microbial nucleic acids, which slows microbial proliferation. In addition to its direct antimicrobial effects, kaempferol exhibits antioxidant activity by scavenging free radicals within microbial cells, thus reducing oxidative stress and weakening microbial defenses. This dual mechanism of action makes kaempferol particularly effective against gram‐positive bacterial and fungal species such as *Aspergillus fumigatus* [95].

### 3.3 Cinnamon

Cinnamon is one of the world's oldest known spices, with an ancient history dating back millennia. The cinnamon spice is derived from the inner bark of trees belonging to the *Cinnamomum* genus and is primarily from two species known as *Cinnamomum verum* (commonly known as “true cinnamon” or “Ceylon cinnamon”) and *Cinnamomum cassia* (widely known as “Chinese cinnamon” or “cassia”). Although both species belong to the *Lauraceae* family, they vary in taste, texture, and chemical composition. Ceylon cinnamon, native to Sri Lanka, is known for its delicate flavor and soft, crumbly texture, while cassia originates from China and has a stronger, spicier flavor and thicker bark. The primary phytochemicals in cinnamon include cinnamaldehyde, eugenol, cinnamic acid, coumarin, and various other flavonoids. These compounds, particularly cinnamaldehyde and eugenol, are responsible for cinnamon's antioxidant, anti‐inflammatory, antimicrobial, and antidiabetic properties. However, coumarin is found in detectable levels only in Cassia.

First, cinnamaldehyde (C_9_H_8_O) is the primary bioactive compound in cinnamon and is a highly reactive phenylpropanoid. As observed in Table 2:3a, this structure consists of a benzene ring attached to α β‐unsaturated aldehyde group (‐CH═CH‐CHO) [96]. This combination gives cinnamaldehyde its characteristic reactivity, distinct aroma, and antimicrobial potency. Cinnamaldehyde's molecular structure facilitates effective interaction with microbial membranes and proteins, making it a potent antimicrobial agent. The lipophilic nature of cinnamaldehyde enables it to embed itself within the lipid bilayers of microbial cell membranes, where it disrupts membrane integrity. This causes an increase in membrane fluidity and permeability, resulting in the leakage of essential intracellular components such as ions, ATP, and metabolites. The loss of these critical cellular components disrupts microbial osmotic balance and energy metabolism, eventually resulting in cell death. Beyond its impact on membranes, cinnamaldehyde also targets microbial proteins and enzymes by forming covalent bonds with reactive thiol groups leading to the inactivation of enzymes essential for microbial survival, including those involved in protein synthesis, energy production, and cell wall biosynthesis [97]. This disruption of enzymatic function further debilitates microbial cells by halting critical processes such as protein assembly and DNA replication, eventually slowing or stopping microbial growth. Cinnamaldehyde's antimicrobial spectrum is broad, making it effective against both gram‐positive bacteria such as *Streptococci* and *Staphylococci* strains and gram‐negative bacterial strains such as *E.coli* [98], as well as fungal pathogens such as *Candida albicans* [99]. Cinnamaldehyde's ability to target multiple microbial pathways reduces the likelihood of resistance development. Additionally, cinnamaldehyde has proven to be effective in inhibiting the formation of biofilms through disruption to quorum sensing and overall disruption to bacterial colony architecture and adhesion, thus reducing the persistence and resistance of pathogens [100]. Overall, cinnamaldehyde's dual membrane disruption, enzyme inhibition mechanisms, and antibiofilm properties make it an excellent antimicrobial phytochemical.

Second, eugenol (C_10_H_12_O_2_) is a potent phenolic compound found in cinnamon and other spices like cloves. As observed in Table 2:3b, eugenol is composed of a benzene ring attached to a methoxy group (‐OCH_3_) and a hydroxyl group (‐OH) at different positions, enhancing its chemical reactivity and biological activity. The methoxy group increases the eugenol's lipophilicity. In contrast, the hydroxyl group facilitates hydrogen bonding with microbial proteins, enabling integration into the lipid bilayers of microbial membranes, which alters the membrane's fluidity and disrupts its structural integrity, leading to increased membrane permeability [101]. As membrane permeability increases, essential intracellular components leak out, severely compromising the microbial cell's homeostasis and energy balance, ultimately resulting in cell death. In addition to its membrane‐disruptive properties, eugenol targets microbial enzymes such as enzymes involved in DNA replication, protein synthesis, and cell wall biosynthesis. The hydroxyl group in eugenol forms hydrogen bonds with active sites on microbial proteins and enzymes, inactivating them and preventing essential metabolic functions from occurring. This dual ability to disrupt membranes and inhibit essential enzymes makes eugenol highly effective against many pathogens, such as gram‐positive bacteria like *Staphylococcus aureus*, which are vulnerable to the eugenol's effects on cell wall integrity, and fungal pathogens like *Candida albicans* because eugenol disrupts the synthesis of vital components essential for fungal growth and reproduction [102]. Moreover, eugenol's antioxidant properties further contribute to its antimicrobial efficacy because eugenol reduces oxidative stress and disrupts microbial survival in hostile environments, leading to cell death. In contrast, simultaneously, its antioxidant effect can protect host cells from oxidative damage, making eugenol a powerful antimicrobial agent, reducing collateral damage to human and host tissues during infections. This combination of membrane disruption, enzyme inhibition, and antioxidant action makes eugenol a highly potent antibacterial compound, widely recognized for its ability to combat bacterial, fungal, and even viral pathogens while providing protective benefits to host cells [103].

Additionally, cinnamic acid (C_9_H_8_O_2_) is a phenylpropanoid found in cinnamon and, as observed in Table 2:3c, is characterized by a benzene ring attached to a propenoic acid group, providing an amphiphilic nature, which facilitates interactions with both lipophilic and hydrophilic components of microbial cells. This dual nature enables cinnamic acid to disrupt microbial membranes through the integration into lipid bilayers, where its hydrophobic portion destabilizes membrane structure, increases permeability, and causes the leakage of vital intracellular contents such as ions and metabolites. Additionally, cinnamic acid interferes with microbial energy metabolism by inhibiting enzymes in ATP production, thereby reducing the energy available for cellular processes. It also inhibits cell wall biosynthesis, further disrupting the structural integrity of microbial cells. This combined action and cinnamic acid's amphiphilic nature makes it highly effective against a wide range of pathogens, particularly gram‐negative bacteria such as *Escherichia coli* and fungal pathogenic species such as *Aspergillus niger* [104].

Furthermore, procyanidins are flavonoid polymers in cinnamon and, as observed in Table 2:3d, are made up of catechin and epicatechin subunits linked by carbon‐carbon bonds. These large polyphenolic molecules are characterized by multiple hydroxyl groups (‐OH), which equips them with unique hydrophilic properties and the ability to form hydrogen bonds with microbial proteins and DNA [105]. Procyanidins exert antimicrobial activity through interactions with microbial protein and nucleic acids, inhibiting protein synthesis and DNA replication. Their hydrogen bonding with microbial DNA disrupts transcription and replication processes, effectively slowing microbial growth and proliferation. Additionally, procyanidins possess strong antioxidant activity and neutralize ROS within microbial cells and reduce oxidative stress, thus decreasing microbial survival in hostile environments. Procyanidins are particularly effective against gram‐positive bacteria such as *Bacillus subtilis* and fungal pathogenic strains such as *Candida albicans* [106]. Furthermore, they have been shown to enhance the immune system's response to infections, making them an essential component in cinnamon's antimicrobial defencs.

Moreover, coumarin (C_9_H_6_O_2_) is a benzopyrone compound found in undetectable concentrations in *Cinnamomum Verum* but in high concentrations in *Cinnamomum Cassia* (Table 2:3e). Coumarin is characterized by a benzene ring fused to a pyrone ring, which gives it lipophilic properties that effectively disrupt microbial membranes by integrating into microbial lipid bilayers, which increases membrane fluidity and permeability and leads to the leakage of essential intracellular components, ultimately resulting in microbial cell death. In addition to coumarin's membrane‐disruptive properties, it also exhibits enzyme‐inhibitory activity. Coumarin is especially effective against enzymes involved in DNA replication and protein synthesis, as it impairs microbial growth and replication by interacting with the active sites on these critical enzymes. This dual mechanism makes coumarin effective against a broad range of pathogens, including gram‐negative and gram‐positive bacterial and cumi.

Finally, catechins are another type of flavonoid found in cinnamon (Table 2:3f) and are equipped with a benzopyran ring structure and multiple hydroxyl groups, which facilitate the exertion of strong antimicrobial effects through the disruption of key microbial processes. The hydroxyl groups enable catechins to form hydrogen bonds with microbial nucleic acids, interfering with DNA replication and transcription, thus inhibiting overall microbial growth and reproduction. In addition to their direct antimicrobial action, catechins act as powerful antioxidants, reduce oxidative stress within microbial cells by ROS, and weaken microbial survival in hostile conditions. Catechins are particularly effective against bacterial species such as *Staphylococcus aureus* and fungal pathogens such as *Aspergillus niger* [107].

### 3.4 Cloves

Cloves (*Syzygium aromaticum*) are rich in bioactive compounds, with eugenol being the most abundant and responsible for their characteristic aroma and many of their medicinal properties. Cloves also contain other vital phytochemicals such as acetyl eugenol, beta‐caryophyllene, flavonoids, tannins, and triterpenoids, each contributing to cloves’ diverse and unique pharmacological activities. Eugenol's structures, functionality, and mechanisms have already been discussed in Section 2.3.

However, there are various other important constituents in cloves that contributes to its antimicrobial properties such as beta‐caryophyllene (C_15_H_24_) is a sesquiterpene found abundantly in clove essential oil. As observed in Table 2:4a, beta‐caryophyllene possesses a cyclic hydrocarbon configuration featuring two fused rings and multiple double bonds. This unique architecture contributes to its strong lipophilic properties, essential for seamless integration into lipid bilayers, leading to increased membrane destabilization and disruption. Once integrated into the membrane, it disturbs the structural arrangement of lipids, weakening the membrane's integrity and increasing its permeability, resulting in the leakage of intracellular contents and leading to the disruption of cellular homeostasis and, eventually, microbial cell death. Beta‐caryophyllene has demonstrated effectiveness against a broad spectrum of microorganisms, including gram‐positive bacteria, such as *Staphylococcus aureus*, gram‐negative bacteria, such as *Escherichia*, and fungal pathogens, such as *Candida albicans* [108]. Beyond its direct antimicrobial effects, beta‐caryophyllene exhibits anti‐inflammatory properties that enhance its therapeutic potential. During microbial infections, the host's immune response triggers inflammation, and by reducing inflammation and oxidative stress, beta‐caryophyllene not only helps to limit the damage caused by the immune response but also improves overall antimicrobial efficacy. This is because inflammatory responses can create an environment that fosters microbial growth and survival [108]. This dual action of antimicrobial activity and inflammation control positions beta‐caryophyllene as a particularly useful compound in treating infections where excessive inflammation is a concern.

Similarly, tannins are polyphenolic compounds that, as observed in Table 2:4b, consist of multiple hydroxyl groups attached to an aromatic ring structure, making them large, water‐soluble molecules capable of forming hydrogen bonds with proteins and other macromolecules. These hydroxyl groups allow tannins to exert antimicrobial activity by binding to and precipitating microbial proteins, particularly those located in the cell membrane and cell wall. This interaction leads to protein denaturation and destabilization of the cell wall, increasing microbial vulnerability to osmotic stress and immune system attacks. Additionally, tannins interfere with microbial enzyme systems, inhibiting critical processes such as DNA replication and energy metabolism, which makes them effective against a wide range of bacterial and fungal pathogens. Moreover, tannins possess strong antioxidant properties, which neutralize ROS, to increase the susceptibility of microbes to oxidative stress, and reduce microbial survivability [109].

Additionally, gallic acid (C_7_H_6_O_5_), as seen in Table 2:4c, is a phenolic acid consisting of a benzoic acid structure, which contains three hydroxyl groups that form hydrogen bonds and chelate metal ions. This reactivity underpins gallic acid's antimicrobial solid activity, as it can disrupt microbial membranes by penetrating lipid bilayers, which increases their permeability and causes the leakage of essential intracellular contents. Additionally, gallic acid inhibits microbial enzymes by binding to metal cofactors and active site residues, interfering with crucial processes such as DNA replication, protein synthesis, and ATP production. This ability to disrupt fundamental cellular functions makes it effective against many pathogens, including gram‐negative bacteria, gram‐positive bacteria, and fungal species. Beyond its direct antimicrobial action, gallic acid's antioxidant properties reduce oxidative stress within microbial cells, further weakening their defense mechanisms and enhancing its overall antibacterial efficacy.

Finally, oleanolic acid (C_30_H_48_O_3_), as observed in Table 2:4d, is a triterpenoid compound found in cloves. It has a large pentacyclic structure and multiple hydroxyl groups. Oleanolic acid's lipophilic structure enables integration into microbial lipid bilayers, disrupting membrane integrity and increasing permeability, which leads to the leakage of essential intracellular contents and eventually results in microbial death. Additionally, oleanolic acid inhibits key enzymes involved in DNA replication and protein synthesis by forming hydrogen bonds with nucleophilic residues in the active sites of these enzymes. This enzyme inactivation disrupts microbial growth and replication, making it effective against a broad spectrum of pathogens, such as gram‐positive and gram‐negative bacteria and other fungal pathogenic species.

### 3.5 Ginger

Ginger (*Zingiber officinale*) is a widely used spice and medicinal herb containing a wide array of bioactive phytochemicals, which contribute to its therapeutic effects and antimicrobial properties. The primary phytochemicals in ginger include gingerols, shogaols, zingiberene, zingerone, and paradols. These compounds, especially gingerol and shogaol, play a central role in ginger's antimicrobial, antioxidant, and anti‐inflammatory activities.

First, gingerols, especially [6]‐gingerol, are the most prominent phytochemicals in fresh ginger and are recognized for their potent broad‐spectrum antimicrobial activity against bacterial and fungal pathogens. Structurally, as observed in Table 2:5a, gingerols are classified as phenolic ketones made up of a 4‐hydroxy‐3‐methoxyphenyl group (a benzene ring with hydroxyl and methoxy groups) attached to an alkyl chain containing a hydroxyl and keto (‐CO) group [110]. The unique combination of these functional groups contributes to the high reactivity of gingerols, mainly through hydrogen bonding and nucleophilic interactions, facilitating engagement with microbial targets at multiple sites. The antimicrobial efficacy of gingerols arises from their ability to integrate and disrupt microbial cell membranes. The lipophilic tail of [6]‐gingerol allows partitioning into the lipid bilayers of bacterial and fungal cell membranes, which disrupts membrane fluidity and stability, leading to increased membrane permeability and the loss of essential cellular contents, resulting in microbial cell death. Additionally, the phenolic hydroxyl group in gingerol causes enzyme inhibition as it reacts and covalently bonds with thiol groups in the active sites of essential microbial enzymes involved in DNA replication, protein synthesis, and ATP production, leading to their inactivation, which impairs vital metabolic functions and eventually leads to microbial cell death. Gingerol demonstrates activity against gram‐positive and gram‐negative bacteria, but the mechanisms differ slightly due to the structural differences between these bacterial cells. In gram‐negative bacteria such as *Escherichia coli* and *Pseudomonas aeruginosa*, which have additional outer membranes made of LPS, gingerol can permeate this outer layer. Once inside, it destabilizes the outer and inner membranes, leading to increased permeability and eventual cell lysis. The ability to penetrate this outer membrane barrier is critical, as gram‐negative bacteria are typically more resistant to antimicrobial agents due to the presence of this protective layer [111]. However, in gram‐positive bacteria such as *Staphylococcus aureus*, which lack the outer membrane but have a thick peptidoglycan layer, gingerol exerts its antimicrobial effects by disrupting this peptidoglycan matrix, increasing the permeability of the cytoplasmic membrane. This disruption results in leakage of cellular contents and cell death, demonstrating gingerol's broad‐spectrum activity across different bacterial types. Gingerols also exhibit strong antifungal properties, particularly against pathogenic species such as *Candida*, where their antifungal action is primarily attributed to interference with ergosterol synthesis, as ergosterol is critical for the maintenance of the structural integrity and fluidity of fungal membranes [112]. Through inhibiting the enzymes involved in ergosterol biosynthesis, gingerols compromise the integrity of the fungal membrane, resulting in increased fungal cell permeability and, eventually, cell death.

Similarly, shogaols, the dehydrated forms of gingerols found in higher concentrations in dried or processed ginger, possess a more reactive structure. This is due to their α,β‐unsaturated ketone side chain, which can be observed in Table 2:5b. This structural difference makes shogaols more electrophilic and reactive than gingerols, contributing to their enhanced antimicrobial and antioxidant properties. Shogaols exhibit more potent antimicrobial activity by integrating into microbial cell membranes, destabilizing them and increasing membrane permeability, boosting cell death. Additionally, their electrophilic ketone group can covalently bind to cysteine residues in microbial enzymes, inhibiting critical enzymes involved in energy metabolism, DNA replication, and cell wall synthesis. Shogaols are especially effective against antibiotic‐resistant strains such as MRSA, as they disrupt bacterial defenses and impair their ability to repair damaged membranes, making them powerful agents in combating microbial pathogenic strains that are resistant to traditional antibiotics.

Additionally, zingerone is a bioactive compound found in processed or cooked ginger. Table 2:5c shows that it has a phenolic aldehyde structure with a vanillyl group attached to a propyl chain containing a carbonyl group, making it highly reactive. This reactivity allows zingerone to exert antimicrobial effects through integration with microbial cell membranes, disrupting lipid bilayers and causing leakage of intracellular contents. Zingerone also inhibits microbial enzymes, leading to the impairment of essential cellular functions. In addition to its antimicrobial properties, zingerone is a potent antioxidant and anti‐inflammatory agent, which neutralizes free radicals and reduces oxidative stress, further contributing to microbial dysfunction and death. Although zingerone's potency is slightly lower than that of gingerol, its combined antimicrobial and antioxidant properties make it a valuable compound in ginger's bioactivity.

Furthermore, paradols are formed from gingerols exposed to thermal processing. As observed in Table 2:5d, paradols retain the phenolic group of gingerols but feature a modified side chain, typically with a saturated or unsaturated carbonyl group. This structure allows paradols to target microbial cell membranes, increasing permeability and leading to cell lysis. Additionally, they exhibit enzyme‐inhibiting activity by interacting with microbial proteins, disrupting essential processes within the cells. These combined actions make paradols effective in combating both bacterial and fungal infections.

Finally, zingiberene, the primary sesquiterpene found in ginger essential oil, is significant in its antimicrobial and anti‐inflammatory properties. Zingiberene is particularly effective against gram‐positive bacteria such as *Streptococcus* mutants. Additionally, zingiberene works synergistically with other ginger compounds like gingerol and shogaol, enhancing their overall antimicrobial activity by increasing the permeability of microbial membranes, allowing these other compounds to penetrate quickly and act more effectively.

### 3.6 Black Pepper

Black pepper (*Piper nigrum*) contains a variety of bioactive phytochemicals, with piperine being the most prominent compound responsible for its unique character. In addition to piperine, black pepper contains other interesting phytochemical compounds such as volatile oils including sabinene, limonene, pinene, and caryophyllene, flavonoids, and alkaloids, each contributing to black pepper's unique antimicrobial, antioxidant, and anti‐inflammatory effects. Piperine, however, plays a crucial role in black pepper's antimicrobial activity, targeting a wide range of bacterial, fungal, and viral pathogens.

First, piperine (C_17_H_19_NO_3_) is the primary bioactive alkaloid found in black pepper, and as observed in Table 2:6a, it consists of a nitrogenous piperidine ring linked to a phenyl ring via a conjugated double‐bond system. This structure, along with the methylenedioxy group attached to the phenyl ring, enhances piperine's reactivity and ability to engage in hydrophobic interactions with biological membranes [113]. The lipophilic nature of piperine allows for easy integration into microbial lipid bilayers, which increases microbial cell membrane permeability, leading to the leakage of essential intracellular components and eventually resulting in microbial cell death. Piperine's antimicrobial action also inhibits crucial microbial enzymes through its interactions with enzyme active sites, specifically through the modification of reactive thiol groups in enzymes, leading to the disruption and inhibition of vital metabolic pathways that hinder microbial development and replication. These antibacterial mechanisms are effective against a wide spectrum of pathogens; in gram‐negative bacteria such as *Escherichia coli* and *Salmonella enterica*, piperine's lipophilicity allows penetration into the LPS outer barrier and destabilizes both the outer and inner membranes, resulting in increased susceptibility and eventual cell death. For gram‐positive bacteria such as *Staphylococcus aureus* and *Bacillus subtilis*, which have a thick peptidoglycan layer but lack an outer membrane, piperine disrupts the cytoplasmic membrane, leading to the leakage of cellular contents and inhibition of key enzymes involved in cell wall synthesis, effectively halting bacterial growth [114]. Furthermore, piperine demonstrates potent antifungal activity against *Candida albicans* and *Aspergillus niger*, where piperine disrupts fungal ergosterol synthesis, compromising the structural stability of fungal membranes, increasing permeability and leading to the leakage of cytoplasmic contents. Piperine further enhances its antifungal effect through the inhibition of cytochrome P450 enzymes in fungi, an enzyme group essential for sterol biosynthesis, including ergosterol. This dual membrane disruption and enzyme inhibition mechanism makes piperine particularly effective against fungal infections, disrupting fungal growth and replication [115]. Moreover, piperine enhances the efficacy of other antimicrobial agents, particularly antibiotics, by inhibiting microbial efflux pumps, which are proteins used by bacteria to expel antibiotics from their cells. Therefore, by blocking these pumps, piperine increases the intracellular concentration of antibiotics, enhancing their antimicrobial activity and overcoming bacterial resistance mechanisms. This synergistic action not only restores the potency of antibiotics against resistant strains but also reduces the required dosage, minimizing potential side effects and slowing the development of further resistance [116].

In addition to piperine, black pepper contains volatile oils such as sabinene, limonene, pinene, and caryophyllene, which add to its antimicrobial properties. These volatile oils are primarily terpenes such as sabinene, limonene, and pinene monoterpenes, while caryophyllene is a sesquiterpene. Their cyclic structures facilitate efficient lipophilic interactions with microbial cell membranes, and their hydrophobic nature facilitates integration into the lipid bilayer of microbial membranes, disrupting membrane fluidity and permeability, which impairs crucial processes such as microbial respiration by interference with the electron transport chain and inhibiting ATP production, ultimately leading to cell death. Volatile oils are particularly effective against gram‐positive bacteria and fungi, where they compromise membrane integrity, weaken cell wall structures, and inhibit crucial microbial enzymes, further enhancing their antimicrobial effects [117].

Furthermore, black pepper contains flavonoids like quercetin, kaempferol, and alkaloids. These flavonoids exhibit strong antioxidant properties, protecting cells from oxidative damage and indirectly reducing microbial viability. In addition to their antioxidant effects, these flavonoids inhibit microbial growth by targeting critical metabolic pathways and enzymes essential for microbial survival. Aside from piperine, the alkaloids also contribute to black pepper's bioactivity by modulating cellular processes and working synergistically with other bioactive compounds, enhancing their antimicrobial potency and effectiveness in combating microbial pathogens [118].

### 3.7 Cumin

Cumin (*Cuminum cyminum*) is a spice widely used in culinary and medicinal practices, and it contains a wide array of bioactive phytochemicals that contribute to its therapeutic and antimicrobial properties. The most prominent phytochemicals in cumin include cumin aldehyde and terpenes such as p‐cymene, terpinene, limonene, flavonoids, and phenolic acids. Among these, cumin aldehyde is the most important compound responsible for cumin's robust antimicrobial activity. However, the synergistic effects of terpenes and flavonoids further enhance cumin's ability to inhibit microbial growth.

First, cuminaldehyde is the primary bioactive compound in cumin, and it exhibits potent antimicrobial properties through its ability to disrupt microbial membranes and inhibit key enzymes, making it effective against a wide range of pathogens. Structurally, as observed in Table 2:7a, cuminaldehyde (C_10_H_12_O) is a phenolic aldehyde composed of a benzene ring with an aldehyde group (‐CHO) and an isopropyl group attached. This configuration, particularly the conjugation between the aromatic ring and the aldehyde group, gives cuminaldehyde high reactivity and bioactivity. The aldehyde group is highly electrophilic, facilitating interactions with microbial proteins and enzymes [119]. Its lipophilic nature facilitates cuminaldehyde integration into microbial cell membranes, destabilizing lipid bilayers and increasing membrane permeability, eventually leading to cellular death due to disruptions in osmotic balance and energy production. In addition, cuminaldehyde inhibits key microbial enzymes, as the aldehyde group reacts with thiol groups in cysteine residues of enzymes, which are crucial for microbial catalytic activity. Through this inactivation, cuminaldehyde impairs vital processes such as DNA replication, protein synthesis, and energy metabolism, further crippling microbial cells. This dual mechanism of membrane destabilization and enzyme inhibition makes cuminaldehyde effective against a broad spectrum of pathogens, including bacterial species such as *Escherichia coli*, *Salmonella enterica*, and *Staphylococcus aureus*, and fungal species such as *Candida albicans* [95]. Cuminaldehyde also exhibits synergistic antimicrobial effects when combined with other antimicrobial agents; because of the weakening of microbial membranes and inhibition of enzymatic activity, it enhances the permeability of microbial cells to other compounds, improving the efficacy of antibiotics and other treatments. This makes cuminaldehyde particularly valuable in combating antibiotic‐resistant bacterial strains, where it can improve the efficacy of conventional therapies [120]. Overall, cuminaldehyde's high reactivity and ability to target both microbial structural and metabolic components position it as a powerful natural antimicrobial agent with broad‐spectrum efficacy against various bacterial and fungal pathogens.

Second, cumin is rich in terpenes, such as p‐cymene, gamma‐terpinene, and limonene, which are crucial for enhancing antimicrobial activity. Terpenes are hydrocarbons derived from isoprene units with p‐cymene (C_10_H_14_), as observed in Table 2:7b, being a monoterpene consisting of a benzene ring with methyl and isopropyl groups, while limonene (C_10_H_16_), as observed in Table 2:7c, is a cyclic monoterpene. These hydrophobic molecules integrate into microbial cell membranes, disrupting membrane integrity, increasing membrane permeability, and leading to the leakage of intracellular contents, ultimately causing cell death. Limonene disrupts explicitly the stability and fluidity of microbial membranes, increasing their vulnerability to environmental stress and immune attacks. Moreover, the terpenes in cumin complement cuminaldehyde, enhancing its antimicrobial efficacy by further increasing membrane permeability and allowing cuminaldehyde and other bioactive compounds to penetrate microbial cells more effectively [121]. This synergistic interaction is particularly beneficial in combating resistant strains of bacteria and fungi, making cumin a potent antimicrobial agent.

Additionally, phenolic, caffeic, and chlorogenic acids are crucial in cumin's bioactivity through their antimicrobial and antioxidant properties. Structurally, these compounds are characterized by a benzene ring with carboxyl (‐COOH) and hydroxyl (‐OH) groups, making them highly reactive. Their antimicrobial activity stems from their ability to disrupt microbial cell walls by binding to microbial proteins and polysaccharides, destabilizing the cell wall structure and increasing the microbes' susceptibility to environmental stress. Additionally, phenolic acids inhibit microbial enzymes such as those involved in the citric acid cycle and ATP production, impairing microbial energy metabolism and hindering survival. Phenolic acids also exhibit potent antioxidant properties, neutralizing ROS and protecting host cells from oxidative damage during infections [122]. This indirectly enhances immune function and supports the body's ability to combat microbial pathogens.

### 3.8 Cardamom

Cardamom (*Elettaria cardamomum*), often referred to as the “queen of spices,” is prized for its culinary value and its decadent array of bioactive phytochemicals that exhibit significant medicinal and antimicrobial activity. The main bioactive compounds in cardamom include 1,8‐cineole (eucalyptol), α‐terpineol, terpinyl acetate, limonene, sabinene, and borneol, all of which contribute to cardamom's antioxidant, antimicrobial, and anti‐inflammatory effects. These compounds, particularly 1,8‐cineole and α‐terpineol, play a prominent role in cardamom's ability to inhibit microbial growth. First, 1,8‐cineole, also known as eucalyptol, is a highly bioactive compound abundant in cardamom essential oil and is valued for its potent antimicrobial and anti‐inflammatory properties. Structurally, 1,8‐cineole (C_10_H_18_O), as stated in Table 2:1c, is a monocyclic ether classified as a monoterpenoid oxide consisting of a ten‐carbon backbone derived from two isoprene units, with an oxygen atom embedded in a cyclic ether group. This unique structure gives cineole distinctive polarity and reactivity, effectively allowing interactions with microbial membranes and proteins [123]. The oxygen atom in the ether group facilitates interactions with the polar heads of phospholipids in microbial membranes, altering membrane dynamics. Similarly, 1,8‐cineole's lipophilic properties facilitate penetration of the microbial cell wall and ease integration into the lipid bilayer, increasing microbial cell permeability and eventually resulting in cell death. Furthermore, 1,8‐cineole inhibits microbial enzymes, and this dual mechanism of action, involving membrane destabilization and enzyme inhibition, makes 1,8‐cineole highly effective against a broad spectrum of pathogens, including gram‐negative bacteria, such as *Escherichia coli* and *Pseudomonas aeruginosa*, gram‐positive bacteria, such as *Staphylococcus aureus* and *Bacillus subtilis*, and fungal pathogens such as *Candida* [85].

Similarly, α‐terpineol (C_10_H_18_O) is another critical bioactive compound found in cardamom (Table 2:8a) and is a monoterpenoid alcohol with a hydroxyl group attached to a cyclic structure, which enhances its reactivity. The hydroxyl group facilitates α‐terpineol in bonding hydrogen with microbial proteins and enzymes, disrupting their function. In contrast, its cyclic structure facilitates hydrophobic interactions with the lipid bilayers of microbial membranes. This dual characteristic enables α‐terpineol to disrupt microbial membranes by effectively increasing membrane permeability and fluidity [124]. Additionally, α‐terpineol forms covalent bonds with thiol groups on microbial enzyme active sites, inhibiting key enzymes involved in critical microbial processes such as DNA replication, protein synthesis, and cell wall biosynthesis, thereby inhibiting overall microbial growth and replication. α‐Terpineol's antibacterial efficacy is effective against gram‐positive bacteria such as *Streptococcus pneumoniae*, gram‐negative bacteria such as *Klebsiella pneumoniae*, and fungal pathogens like *Aspergillus niger* and *Candida albicans* [125].

Additionally, terpinyl acetate is an ester derivative of terpineol and is known for its effectiveness against many bacterial and fungal pathogens. Terpinyl acetate (C_12_H_20_O_2_), as stated in Table 2:8b, is formed from terpineol and acetic acid, where the hydroxyl group (‐OH) of terpineol is replaced by an acetyl group (‐COCH_3_), making the compound more hydrophobic and less polar [126]. This architecture enhances terpinyl acetate's ability to integrate into lipid membranes through hydrophobic interactions, increasing its capacity to disrupt microbial cell membranes. The enhanced lipophilicity of terpinyl acetate facilitates the penetration of the lipid bilayers of microbial membranes, leading to increased fluidity and permeability. This disruption causes the leakage of intracellular components, compromising cell integrity and ultimately leading to microbial cell death. Despite terpinyl acetate being less reactive than α‐terpineol, it still interferes with microbial enzymes by interacting with their active sites, disrupting key metabolic processes essential for survival, such as protein synthesis and energy production [127]. This dual mechanism of action makes terpinyl acetate a potent antimicrobial agent effective against a broad spectrum of pathogens.

Furthermore, various terpenes, flavonoids, and phenolic compounds bolster cardamom's antimicrobial activity. Terpenes such as limonene, sabinene, and borneol are crucial in targeting microbial membranes and enzymes. Sabinene (Table 2:8c) is a monoterpene that interacts with microbial membranes, enhancing permeability and fluidity and leading to microbial cell death, particularly in bacterial and fungal cells [128]. Additionally, borneol, as observed in Table 2:8d, is a terpene alcohol that contributes to antimicrobial effects through membrane disruption and enzyme inhibition. Its hydroxyl group facilitates hydrogen bonding with enzyme active sites, thereby impairing catalytic functions.

Finally, cardamom's flavonoids and phenolic compounds further enhance its antimicrobial efficacy. Flavonoids such as kaempferol and quercetin inhibit microbial growth by disrupting cell wall synthesis and interacting with microbial DNA. At the same time, their antioxidant properties help reduce microbial survival under oxidative stress. Phenolic acids such as caffeic acid and chlorogenic acid also contribute to antimicrobial activity by interfering with microbial enzymes and disrupting membrane integrity, further weakening microbial defenses. This combination of terpenes, flavonoids, and phenolic acids makes cardamom a potent natural antimicrobial agent against many pathogens, including bacteria and fungi.

### 3.9 Bay Leaf

Bay leaf (*Laurus nobilis*), widely used as a culinary spice and medicinal herb, contains a rich profile of bioactive phytochemicals, many of which contribute to its potent antimicrobial, antioxidant, and anti‐inflammatory properties. The primary bioactive compounds responsible for its antimicrobial effects include 1,8‐cineole (eucalyptol), linalool, methyl eugenol, sabinene, and α‐terpineol. These compounds, particularly 1,8‐cineole and linalool, as shown in Table 2:9a. work through various mechanisms to disrupt microbial membranes and inhibit critical microbial enzymes, making bay leaf an effective natural antimicrobial agent [129].

### 3.10 Nutmeg

Nutmeg (*Myristica fragrans*) contains a variety of bioactive phytochemicals that contribute to its medicinal properties, particularly its antimicrobial activity. The primary bioactive compounds responsible for nutmeg's antimicrobial effects include myristicin, safrole, eugenol, and terpenes such as α‐pinene, limonene, and sabinene.

First, nutmeg's most abundant bioactive compound is myristicin, known for its potent antimicrobial properties. Structurally, as stated in Table 2:10a, myristicin (C_11_H_12_O_3_) is a phenylpropene compound, specifically a methylenedioxy benzene derivative consisting of a benzene ring attached to a methylenedioxy group (‐O‐CH_2_‐O) and a propenyl side chain. The methylenedioxy group enhances myristicin's ability to engage in electrophilic reactions, facilitating interactions with microbial lipids, enzymes, and proteins. Its phenylpropene backbone also contributes to its hydrophobic nature, facilitating easy integration into microbial cell membranes [130]. Additionally, myristicin's lipophilic nature allows integration into the lipid bilayers of bacterial and fungal cell membranes, increasing membrane permeability. This leads to the loss of essential cellular components, thus disrupting microbial osmotic balance, homeostasis, and energy production, which eventually results in microbial cell death. Furthermore, myristicin inhibits key microbial enzymes as its methylenedioxy group reacts with nucleophilic sites in the active sites of microbial enzymes involved in DNA replication, protein synthesis, and cell wall biosynthesis, which leads to their inactivation and hinders microbial growth and reproduction. Myristicin is effective against a broad spectrum of bacterial and fungal pathogens, including gram‐negative bacteria such as *Escherichia coli*, gram‐positive bacteria such as *Staphylococcus aureus*, and fungal pathogens such as *Candida albicans* [131].

Additionally, safrole (Table 2:10b) is a phenylpropene compound found in nutmeg and is known for its notable antimicrobial properties. Structurally, safrole (C_10_H_10_O_2_) is a methylenedioxy benzene derivative consisting of a methylenedioxy group (‐O‐CH_2_‐O) attached to a benzene ring with a propenyl side chain. This structural similarity to myristicin enhances its reactivity primarily due to the methylenedioxy group, which boosts its electrophilic nature. This increased reactivity facilitates effective interaction with microbial proteins and lipids, disrupting important microbial processes. Safrole exerts antimicrobial activity through integration into microbial lipid bilayers, destabilizing membrane structures and increasing membrane permeability, eventually leading to cellular death. Moreover, safrole inhibits microbial enzymes involved in energy production and DNA replication by interacting with the active sites of enzymes. Safrole is effective against gram‐positive bacteria such as *Staphylococcus aureus*, gram‐negative bacteria such as *Salmonella Typhimurium* and *Pseudomonas aeruginosa*, and fungal pathogens such as *Aspergillus niger* [132].

### 3.11 Basil

Basil (*Ocimum basilicum*) is commonly used in culinary and medicinal contexts. It contains a variety of bioactive phytochemicals that are responsible for its therapeutic properties. The major phytochemicals in basil include eugenol, linalool, 1,8‐cineole (eucalyptol), methyl chavicol (estragole), and camphor. These contribute to basil's ability to inhibit the growth of bacteria, fungi, and viruses.

First, methyl chavicol, or estragole, is a phenylpropene derivative widely recognized for its potent antimicrobial properties. As stated in Table 2:11a, methyl chavicol (C_10_H_12_O) is composed of a benzene ring attached to a propenyl group, with a methoxy group (‐OCH_3_) positioned at the para location on the benzene ring. This methoxy group enhances the compound's lipophilicity, enabling methyl chavicol's effective integration into microbial lipid membranes. The hydrophobic character of the benzene ring facilitates the compound's penetration of lipid bilayers, disrupting overall membrane fluidity and integrity. Following integration, methyl chavicol destabilizes the membrane structure, increasing permeability, which leads to the leakage of cellular components and, eventually, results in cellular death. In addition, methyl chavicol exhibits significant enzyme inhibition as it interacts with thiol groups on the active sites of microbial enzymes, disrupting key microbial processes such as cell wall biosynthesis, protein synthesis, and DNA replication. This dual mechanistic antibacterial action enables methyl chavicol to target both cellular membranes and enzymes, making it highly effective against a wide range of pathogens, including gram‐positive bacteria such as Staphylococcus aureus, gram‐negative bacteria such as *Escherichia coli*, and fungal pathogens like *Candida albicans* [133].

Similarly, camphor is a key terpene in basil essential oil, which plays a significant role in its antimicrobial properties. As shown in Table 2:11b, camphor (C_10_H_16_O) is a bicyclic monoterpene ketone, characterized by two fused rings and a carbonyl group (‐C═O), which enhances its reactivity. The carbonyl group facilitates interactions with microbial proteins and enzymes, while its lipophilic nature enables it to integrate into microbial lipid bilayers. Once embedded, camphor disrupts the membrane structure, increasing membrane permeability and causing the leakage of essential metabolites and ions, which ultimately leads to cell death. Additionally, camphor inhibits microbial enzymes by interacting with their active sites, inactivating key enzymes involved in essential processes such as protein synthesis, DNA replication, and ATP synthesis. This dual mechanism makes camphor effective against a range of microbial pathogens, particularly gram‐positive bacteria such as *Staphylococcus aureus* and fungal pathogens such as *Candida albicans*, contributing to its overall antimicrobial spectrum [134].

### 3.12 Mustard

Mustard (*Brassica* species), particularly *Brassica juncea* (brown mustard) and *Brassica nigra* (black mustard), contains several unique bioactive phytochemicals that are responsible for its potent antimicrobial properties. The essential compounds contributing to mustard's antimicrobial activity are glucosinolates, particularly sinigrin and its hydrolysis product, allyl isothiocyanate (AITC), erucic acid, and phenethyl isothiocyanate (PEITC). Mustard's antimicrobial action is especially effective against bacteria and fungi, including resistant strains.

First, sinigrin, a glucosinolate found in mustard seeds, and its hydrolysis product, AITC, are the primary compounds responsible for mustard's potent antimicrobial properties. As in Table 2:12a, sinigrin (C_10_H_16_KNO_9_S_2_) consists of a glucose molecule attached to a sulfur‐containing isothiocyanate group which, upon enzymatic hydrolysis by myrosinase, breaks down into AITC, a volatile compound known for its strong, spicy aroma and high reactivity. As observed in Table 2:12b, AITC (C_4_H_5_NS) is characterized by an allyl group attached to a thiocyanate (‐N═C═S) group, making it highly electrophilic. This reactivity allows AITC to efficiently interact with nucleophilic sites in microbial proteins, particularly those containing thiol or amine groups, disrupting crucial cellular functions. AITC's lipophilic nature also enables integration into microbial membranes, disrupting the phospholipid bilayers, increasing membrane permeability, and causing leakage of vital intracellular contents. This membrane disruption compromises microbial cell integrity and eventually leads to cell death. In addition, AITC reacts with microbial proteins and enzymes by covalently bonding to thiol groups in enzyme active sites, which leads to the inhibition of vital enzymes and processes. AITC is particularly effective against oxidative stress enzymes, further weakening microbial defenses. Due to these dual mechanisms, AITC exhibits a broad antimicrobial spectrum, showing efficacy against gram‐positive bacteria such as *Staphylococcus aureus*, gram‐negative bacteria such as *Escherichia coli* and *Salmonella enterica*, and fungal pathogens like *Candida albicans* [135].

Similarly, PEITC is a hydrolysis product of the glucosinolate gluconasturtiin found in mustard seeds. As in Table 2:12c, PEITC (C_9_H_9_NS) consists of a phenyl ring attached to an ethyl isothiocyanate group (‐N═C═S), which gives it high reactivity. Like AITC, the isothiocyanate group in PEITC is highly electrophilic, facilitating reactions with nucleophilic amino acids in microbial proteins and enzymes, particularly cysteine residues containing thiol groups. This reactivity enables the disruption of various microbial cellular processes. Additionally, PEITC easily integrates into the microbial lipid bilayer due to its lipophilic nature, which disrupts membrane fluidity and permeability, resulting in the compromise of cellular integrity and ultimately leading to cell death. Furthermore, PEITC inhibits microbial enzymes by forming covalent bonds with thiol groups in enzyme active sites, disrupting essential metabolic processes and halting microbial growth and reproduction. PEITC has demonstrated significant antimicrobial activity against a broad spectrum of pathogens, including gram‐positive bacteria such as *Listeria monocytogenes* and *Staphylococcus aureus*, gram‐negative bacteria such as *Escherichia coli* and *Salmonella typhimurium*, and fungal pathogens such as *Candida albicans* [136].

Finally, erucic acid is a monounsaturated fatty acid found in mustard seed oil. As given in Table 2:12d, erucic acid (C_22_H_42_O_2_) is a long‐chain omega‐9 fatty acid consisting of 22 carbons with a single, double bond between the 13th and 14th carbon atoms. This large, nonpolar hydrocarbon chain gives erucic acid a highly lipophilic nature, enabling easy integration into microbial lipid bilayers. Once embedded in the microbial cell membrane, erucic acid increases membrane fluidity and destabilizes the membrane structure, enhancing permeability. This disruption causes the leakage of vital intracellular components, compromising cellular function, ultimately resulting in microbial cell death. Erucic acid is particularly effective against gram‐positive bacteria such as *Staphylococcus aureus* and exhibits activity against specific fungal pathogens [137].

### 3.13 Fenugreek

Fenugreek (*Trigonella foenum‐graecum*) is a leguminous herb known for its extensive medicinal properties and potent antimicrobial activity. The bioactive compounds in fenugreek, particularly diosgenin, trigonelline, galactomannan, and flavonoids like vitexin and isovitexin, are responsible for its antimicrobial effects. These phytochemicals act through various mechanisms, including membrane disruption, enzyme inhibition, and interference with microbial metabolic pathways, making fenugreek effective against many bacteria, fungi, and viruses.

First, diosgenin (C_27_H_42_O_3_) is a steroidal saponin found in fenugreek and, as stated in Table 2:13a, is a steroidal sapogenin with a complex arrangement of four fused rings (three cyclohexane rings and one cyclopentane ring) featuring a hydroxyl group at the third carbon and a double bond between the fifth and sixth carbons. This unique structure gives diosgenin both hydrophilic and hydrophobic properties, facilitating effective interactions with microbial membranes and proteins. The hydroxyl group enables hydrogen bonding with microbial proteins, while the lipophilic steroidal backbone integrates into microbial lipid bilayers, disrupting membrane integrity and increasing permeability, ultimately resulting in cell death. Diosgenin also inhibits key microbial enzymes involved in cell wall biosynthesis and energy metabolism by interacting with thiol groups and amino groups in enzyme active sites. This enzymatic inhibition further compromises microbial growth due to the disruption of key metabolic pathways. Diosgenin has demonstrated broad‐spectrum antimicrobial activity, being particularly effective against gram‐positive bacteria such as *Staphylococcus aureus* and fungal pathogens such as *Candida albicans* and *Aspergillus niger*, making it a valuable antimicrobial phytochemical in fenugreek [138].

Similarly, trigonelline (C_7_H_7_NO_2_) is an alkaloid found in fenugreek seeds and, as observed in Table 2:13b, is a methylated derivative of nicotinic acid (niacin), featuring a pyridine ring with a methyl group (‐CH_3_) attached to the nitrogen atom, which imparts water solubility and enhances its interaction with microbial proteins and nucleic acids. The pyridine ring facilitates trigonelline effective interaction with microbial DNA, interfering with DNA and protein synthesis by forming hydrogen bonds with nucleic acids. This disruption of DNA replication and transcription processes inhibits microbial growth and slows pathogen proliferation, weakening the microbial ability to cause infection and disease. Additionally, trigonelline acts as an antioxidant by neutralizing ROS within microbial cells, which reduces oxidative stress and weakens microbial cell survival in hostile environments. Its broad antimicrobial activity includes efficacy against both bacterial and fungal pathogens, such as gram‐positive bacteria, including *Staphylococcus aureus*, and fungal species such as *Aspergillus niger* [139].

Galactomannan is a polysaccharide found in fenugreek seeds and contributes to antimicrobial defense alongside its prebiotic benefits. As shown in Table 2:13c, it consists of a mannose backbone with galactose side chains, giving it hydrophilic properties that facilitate interactions with microbial surfaces. By forming hydrogen bonds with microbial cell wall components, particularly in Gram‐positive bacteria such as *Bacillus subtilis*, galactomannan disrupts the peptidoglycan layer, weakening the microbial cell wall and making it more susceptible to environmental stress factors and immune responses. Its hydrophilic nature also absorbs water, increasing osmotic pressure and further destabilizing microbial cells. Additionally, galactomannan supports the growth of beneficial gut bacteria, boosting host immunity and reducing the chance of bacterial infections. Its broad antimicrobial activity has been effective against Gram‐positive bacteria such as *Bacillus subtilis* and Gram‐negative bacteria such as *Escherichia coli* [140].

Finally, vitexin and isovitexin are two flavonoids found in fenugreek, and as given in Table 2:13d,13e, they are both flavone glycosides, consisting of a flavonoid backbone attached to a glucose moiety, with the difference between them being the position of glucose attachment. The hydroxyl groups in vitexin and isovitexin facilitate interactions with microbial proteins and DNA through hydrogen bonding, which disrupts essential processes such as cell wall biosynthesis and DNA replication. This interference in microbial transcription and replication slows down microbial growth and proliferation. Additionally, these flavonoids exhibit potent antioxidant activity, scavenging free radicals and reducing oxidative stress within microbial cells, which weakens microbial defenses and enhances their susceptibility to immune attacks. Vitexin and isovitexin are effective against a range of pathogens, including gram‐positive bacteria such as *Staphylococcus aureus* and fungal species such as *Aspergillus niger* [141].

### 3.14 Lemongrass

Lemongrass (*Cymbopogon citratus*) is rich in several phytochemicals, which are significant in its antimicrobial activity. In addition to well‐known compounds like citral and myrcene, lemongrass contains a variety of other bioactive constituents that contribute to its effectiveness against bacterial, fungal, and viral pathogens.

First, geraniol (C_10_H_18_O), seen in Table 2:14a, is an acyclic monoterpenoid alcohol possessing both hydrophilic and lipophilic properties due to its hydroxyl group and 10‐carbon chain with two double bonds. This structural versatility allows geraniol to interact effectively with microbial cell membranes and proteins. Geraniol exerts broad‐spectrum antimicrobial activity by integrating into microbial lipid membranes, increasing membrane fluidity and causing the leakage of essential intracellular contents, eventually leading to cell death. In addition to membrane disruption, geraniol inhibits key microbial enzymes by interacting with groups in their active sites, interfering with critical cellular processes like DNA replication and protein synthesis. Geraniol is particularly effective against gram‐positive bacterial and fungal pathogens like *Candida albicans*, making it a potent antimicrobial agent [142].

Similarly, cymbopogonol (C_15_H_26_O) is a sesquiterpene alcohol found in lemongrass essential oil and, as displayed in Table 2:14b, is characterized by its 15‐carbon backbone derived from isoprene units and a hydroxyl group that imparts some hydrophilic properties. This structure allows cymbopogonol to exhibit potent antimicrobial activity by integrating into microbial lipid membranes, disrupting membrane fluidity and permeability, which leads to cell death. Additionally, cymbopogonol inhibits key microbial enzymes involved in energy production and cell wall biosynthesis, further hindering microbial growth. This broad‐spectrum antibacterial efficacy includes activity against gram‐positive bacteria such as *Staphylococcus aureus* and gram‐negative bacteria such as *Escherichia coli*, making it a valuable antimicrobial agent [143].

Additionally, as displayed in Table 2:14c, terpinolene (C_10_H_16_) is a cyclic monoterpene with a six‐membered ring and double bonds, and it is a hydrophobic compound that interacts strongly with microbial lipid membranes. Terpinolene's ability to integrate into the lipid bilayer disrupts membrane integrity, leading to increased permeability and leakage of essential intracellular contents, resulting in cell death. In addition to its direct antimicrobial action, terpinolene exhibits antioxidant properties, neutralizing ROS, further weakening microbial defenses. This dual action enhances its antimicrobial effectiveness, particularly against fungal pathogens and gram‐negative bacteria, making terpinolene a potent agent in combating microbial infections [144].

Moreover, as stated in Table 2:14d, elemicin (C_12_H_16_O_3_) is an aromatic ether found in lemongrass. It is characterized by a methoxyphenyl group attached to a propenyl side chain, with its methoxy groups (‐OCH_3_) contributing to its lipophilicity and reactivity. Its primary antimicrobial action stems from its ability to disrupt microbial membranes by embedding itself into the lipid bilayer, thereby increasing membrane fluidity and permeability, which leads to leakage of cellular contents and death. Additionally, the methoxy groups in elemicin can interfere with microbial enzymes by forming covalent bonds with nucleophilic residues in enzyme‐active sites, inhibiting essential processes such as protein synthesis and DNA replication. This dual action of membrane disruption and enzymatic inhibition makes elemicin particularly effective against gram‐positive bacterial and fungal pathogens such as *Aspergillus niger*, contributing to its potent antimicrobial properties [145].

### 3.15 Oregano

Oregano (*Origanum vulgare*) contains a diverse range of bioactive phytochemicals, which collectively contribute to its potent antimicrobial properties. Among these, carvacrol (C_10_H_14_O), shown in Table 2:15a, is a monoterpenoid phenol characterized by a hydroxyl group on a phenolic ring with a hydrophobic isopropyl substituent. This structure enables carvacrol to insert into microbial lipid bilayers, where it disrupts membrane integrity, increases permeability, and causes leakage of ATP and other vital intracellular molecules, ultimately leading to cell death. Carvacrol also destabilizes membrane‐embedded proteins, impairing essential processes such as respiration and nutrient uptake [146].

Similarly, thymol (C_10_H_14_O), an isomer of carvacrol shown in Table 2:15b, has a hydroxyl group positioned differently on the aromatic ring, giving it similar but slightly different bioactivity. Thymol integrates into bacterial membranes, increasing membrane fluidity, causing proton leakage, and disrupting overall ion homeostasis. It also inhibits biofilm formation and quorum‐sensing pathways, thereby reducing microbial virulence [147].

Besides these volatile monoterpenes, oregano contains essential phenolic compounds such as rosmarinic acid (C_18_H_16_O_8_), as shown in Table 2:15c. Rosmarinic acid is a polyphenolic ester made up of caffeic acid and 3,4‐dihydroxyphenyllactic acid, with multiple hydroxyl groups that provide both antioxidant and antimicrobial effects. It neutralizes ROS, weakening microbial defenses, while also inhibiting bacterial enzymes that are involved in nucleic acid and protein synthesis. Rosmarinic acid also disrupts microbial adhesion, preventing colonization and biofilm formation [148].

Furthermore, ursolic acid (C_30_H_48_O_3_), a pentacyclic triterpenoid shown in Table 2:15d, exerts its antimicrobial effects by interacting with microbial membranes and altering lipid order, leading to changes in permeability and cell lysis. Ursolic acid has also been reported to inhibit efflux pumps, thereby increasing intracellular antibiotic accumulation, and to suppress quorum sensing and virulence factors in pathogens such as *Pseudomonas aeruginosa* [149].

## 4 Evidence‐Based Insights Into the Antimicrobial Properties of Spices

Extensive research has previously been conducted on utilizing spice phytochemicals against various pathogenic entities, as shown in Table 3.

**TABLE 3:** Summary of the antibacterial activity of phytochemicals from selected culinary spices, detailing key bioactive compounds, target pathogens, assay methods, minimum inhibitory concentration (MIC) values, and inhibition zones.

Spice	Phytochemical	Microbial species	Antimicrobial testing technique	MIC value	Inhibition zone	References
Cinnamon	Eugenol Cinnamaldehyde	*Cronobacter sakazakii C.malonaticus*	Disc diffusion method	0.512–1.0 mg/mL	n/a	[150]
		*Pseudomonas aeruginosa (P.aeruginosa)* *Escherichia coli (E.coli)* *Staphyloccocus aureus (S.aureus)*		<1 µg/mL	24–22 mm	
Turmeric	Curcumin	*S.aureus* *E.coli*	Disc diffusion method	25 µg/mL	n/a	[151]
		*Staphyloccocus epidermidi*		46.9 µg/mL		
		*Klebsiella pnuemonia* *Enterobacter aerogenes* *Bacillus subtilis*		34 µg/mL		
Garlic	Allicin Ajoene	*E.coli*	TLC‐bioautography technique	0.625 mg/mL	14.03 ± 0.15 mm	[152]
		*Salmonella sp*.		0.325 mg/mL	19.70 ± 0.36 mm	
Cloves	Eugenol	*S.aureus*	Disc diffusion method	0.52 mg/mL	24 mm	[153]
		*E.coli*		0.64 mg/mL	21.9 mm	
Ginger	Zingiberene	*S.aureus*	Agar diffusion method	1 mg/mL	17.1 mm	[154]
		*E.coli*		2.0 mg/mL	12.3 mm	
Black pepper	Piperine	*E.coli*	Agar diffusion method	1.0 µg/mL	17.12–26.13 mm	[155]
		*Listeria monocytogenes (L.monocytes)* *Salmonella typhimurium* *P.aeruginosa*	Broth microdilution	25 mg/mL	n/a	[156]
Cumin	Cuminaldehyde	*Enterococcus faecalis*	Disc diffusion method	14.29 µg/mL	28.75 mm	[157]
		*Br. Thermosphacta* *E.coli* *S.abony*		0.3 mg/mL	>20 mm	[158]
Cardamom	1,8‐Cineole Sabinene α‐Terpinyl acetate	*S. typhimurium*	Disc diffusion method	10 ± 0.00 mg/mL	9.8 ± 0.20 mm	[58]
		*S. mutans* *C. albicans strains*		5 ± 0.00 mg/mL	11.6 ± 0.56 mm	
		*S. aureus*		10 ± 0.00 mg/mL	5.7 ± 0.20 mm	
Bay leaf	Eugenol Cineole	*S. aureus*	Microdilution experimentation	10 mg/mL	n/a	[159]
		*E.coli*		2.5 mg/mL		
		*L. monocytes*		1.25 mg/mL		
Nutmeg	Myristicin	*Streptococcus mutans *	Agar diffusion method	1.25 mg/mL	n/a	[130]
		*Streptococcus mitis*		0.625 mg/mL		
		*S.aureus*		1.25 mg/mL	10.55 ± 0.3 mm	[160]
Basil	Eugenol Linalool	*P.aeruginosa*	Disc diffusion/microtiter plate‐based assay	10.80 mg/mL	11.2 mm	[161]
		*E.coli*		10.80 mg/mL	14.85 mm	
		*S.aureus*		2.45 mg/mL	9.91 mm	
Lemongrass	Citral Limonene Geraniol	*B. thuringiensis*	Broth microdilution	0.15 ± 0 mg/mL	29 ± 2 mm	[162]
		*D. congolensis*		0.15 ± 0 mg/mL	23 ± 2 mm	
		*K. sedentarius*		0.1 ± 0 mg/mL	14 ± 2 mm	
Oregano	Carvacrol Thymol Rosmarinic acid	*S.aureus*	Broth microdilution/disc diffusion Microdilution/agar diffusion	0.125 mg/mL	25–30 mm	[163]
		*E.coli*		0.25 mg/mL	20–24 mm	[163]
		*P.aeruginosa*		0.25–0.5 mg/mL	18–22 mm	[164]
		*L.monocytogenes*		0.125 mg/mL	22–28 mm	[164]
		*C. albicans strains*		0.125–0.25 mg/mL	20–26 mm	[148]

Referenced to Table 3, key phytochemicals such as zingiberene and cuminaldehyde derived from spices like ginger and cumin demonstrate significant activity against notable pathogens. Zingiberene, a sesquiterpene, is particularly effective against foodborne bacteria such as *Salmonella typhimurium, Listeria monocytogenes*, and *Pseudomonas aeruginosa* [165]. These pathogens are associated with foodborne illnesses and opportunistic infections, highlighting the potential of zingiberene as a natural antimicrobial agent for food safety and medical applications. Similarly, cuminaldehyde, a monoterpenoid aldehyde found in cumin, shows potent antibacterial properties, especially against *Pseudomonas aeruginosa* and *Listeria monocytogenes* [166]. Its dual role as a flavoring agent and antimicrobial underscores its potential use in food preservation. Other referenced phytochemicals, such as citral, eugenol, linalool, and cinnamaldehyde, are well‐documented for their antibacterial efficacy. These compounds, derived from lemongrass, cloves, and cinnamon, disrupt bacterial membranes, alter permeability, and inhibit essential enzymes such as eugenol, which has been widely studied for its ability to target gram‐positive bacteria [101]. At the same time, citral demonstrates potent activity against gram‐positive and gram‐negative strains [162]. Furthermore, the methodologies referenced in the table, such as disc diffusion, broth microdilution, and agar diffusion, validate the findings and provide reliable indicators of antibacterial potency, including MIC values and inhibition zones. These values not only standardize the effectiveness of these compounds but also establish their potential for real‐world applications. The studies further suggest that these phytochemicals could be integrated into food preservation systems, antimicrobial coatings, and even therapeutic formulations, particularly for infections caused by resistant pathogens, leading to various novel innovations [167].

The utilization and infusion of phytochemicals for developing antimicrobial applications to combat real‐world pathogenic activity have already begun and are steadily increasing. This has been observed through developing novel wound dressings using cinnamon infusions, which were proven to be effective against *Candida albicans* [168]. This efficacy was further demonstrated through the development of novel “green” wound dressings manufactured using alginate and infused with cinnamon and cloves, which showed efficient antibacterial action against *E.coli* and *S.aureus* species while maintaining excellent biocompatibility and very low cytotoxicity [169]. Furthermore, curcumin and eugenol‐infused biosensors were developed to detect microbial contamination in food and water [170]. Additionally, basil‐infused tablets have successfully been developed for water purification and are now commercially sold to overcome and prevent waterborne diseases such as diarrhea, cholera, and typhoid fever [127]; this highlights the applicability of these natural phytochemicals to combat various pathogenic bacteria and assist in overcoming infections in real‐world settings.

Furthermore, Table 4 presents data and results from various preclinical animal studies conducted to investigate the antimicrobial activity of spice phytochemicals.

**TABLE 4:** Summary of preclinical animal studies investigating the antimicrobial effects of spice‐derived phytochemicals.

Phytochemical	Pathogen	Animal, dose, and route	Main antimicrobial outcomes	Mechanisms
Curcumin [171]	*Staphylococcus aureus*	Mice: 100 mg/kg subcutaneously, administered 2 h after infection and then every 8 h	Significant increase in survival at 24, 48, and 72 h post‐infection, along with reduced lung injury on histological examination	Inhibition of α‐hemolysin pore assembly
Thymol [172]	*Salmonella Typhimurium*	Mice; 50 mg/kg by oral gavage, given 1 day prior to infection and then every 8 h for 5 days	Marked increase in survival (approximately 70–80% compared with 0% in untreated controls), significantly reduced bacterial loads in the caecum, liver and spleen, and decreased levels of proinflammatory cytokines	Inhibition of T3SS‐1 secretion system and anti‐invasion activity
Carvacrol [173]	Multidrug‐resistant *Klebsiella pneumoniae*	Mice; therapeutic dosing (varied by study)	Significant improvement in survival and reduced bacterial burden in vivo; in vitro time‐kill studies confirmed rapid bactericidal activity	Membrane disruption with broad‐spectrum activity
Cinnamaldehyde [174]	*E. coli*	Mice; oral administration	Significant reduction in intestinal colonization compared with untreated controls	Multiple cellular targets, including antiadhesion effects
Cinnamaldehyde [175]	Extra‐intestinal pathogenic *E. coli*	Mice; intraperitoneal administration	Increased survival, reduced bacterial load in tissues, and decreased inflammatory markers compared with controls	Combination of direct antimicrobial and anti‐inflammatory effects
Cinnamaldehyde (adjunct) [176]	MRSA—in vivo synergy with β‐lactam antibiotics	Murine models; combined therapy with cinnamaldehyde and β‐lactams	Restoration of β‐lactam antibiotic activity and increased survival in animals receiving combination therapy compared with monotherapy	Synergistic activity through effects on bacterial membranes and cell walls

Table 4 highlights the preclinical studies conducted using spice phytochemicals to evaluate their antimicrobial activity. These animal studies are crucial for bridging the gap between in vitro results and clinical applications by providing valuable insights into the in vivo efficacy, safety, pharmacokinetics, and potential mechanisms of action of bioactive phytocompounds under physiologically relevant conditions. Compared with in vitro experiments, animal models enable the study of complex interactions between the host, pathogen, and therapeutic agent within an intact biological system. This is particularly important for antimicrobial agents, where factors such as tissue distribution, immune response, and metabolic stability can significantly influence therapeutic outcomes. Additionally, preclinical models facilitate the identification of optimal dosing strategies, the assessment of synergistic effects with conventional antibiotics, and the early detection of potential toxicity, thereby offering a solid evidence base to support the translation of promising natural compounds into clinical trials [177]. Therefore, Table 4 highlights the importance of preclinical trials and further validates the antibacterial properties of phytochemicals.

## 5 Extraction Processes for These Phytochemicals

Additionally, as mentioned in Table 1, these phytochemicals can be extracted from natural spice plant extracts using various methods, including solvent extraction, steam distillation, supercritical fluid extraction, cold pressing, maceration, ultrasound‐assisted extraction, microwave‐assisted extraction, hydrodistillation, solid‐phase extraction, and fermentation [178], where each spice's unique characteristics and properties and its phytochemicals determine the optimal extraction method to obtain the highest yields with minimal waste. The average yield of spice extracts obtained from extraction processes can vary based on factors such as the type of spice, extraction method, and conditions used. These yields can fluctuate depending on various factors, such as the quality of the spice and the specific extraction conditions. However, commercially, the five most utilized manufacturing processes are illustrated below:

### 5.1 Solvent Extraction

**FIGURE 1:**
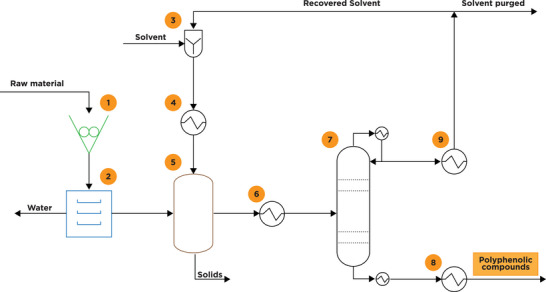
Schematic process flow diagram for the extraction and recovery of polyphenolic compounds from raw material. The process includes raw material pretreatment (1–2), solvent addition and mixing (3–5), solid–liquid separation, heat exchange (6), distillation for solvent recovery (7–9), and final collection of concentrated polyphenolic compounds, with recovered solvent recycled and excess solvent purged [179]. Reproduced from Ref. [179] with permission from Carlos Orrego and Carlos Ariel Cardona.

As shown in Figure 1, solvent extraction is a process that utilizes a liquid solvent, such as ethanol, methanol, hexane, or water, to dissolve phytochemicals from plant material. The solvent penetrates the plant cells, dissolving the desired compounds, which are subsequently separated through solvent evaporation. This method is particularly effective for extracting nonvolatile phytochemicals, including alkaloids, flavonoids, phenolic compounds, and tannins. One of the key advantages of solvent extraction is its efficiency in handling heat‐sensitive compounds, as the process avoids excessive temperatures. Additionally, the choice of solvent can be tailored based on polarity, facilitating selective extraction of specific phytochemicals [180]. On average, solvent extraction produces a yield typically ranging from 5 to 10% on a dry‐weight basis [72].

### 5.2 Supercritical CO_2_ Extraction

**FIGURE 2:**
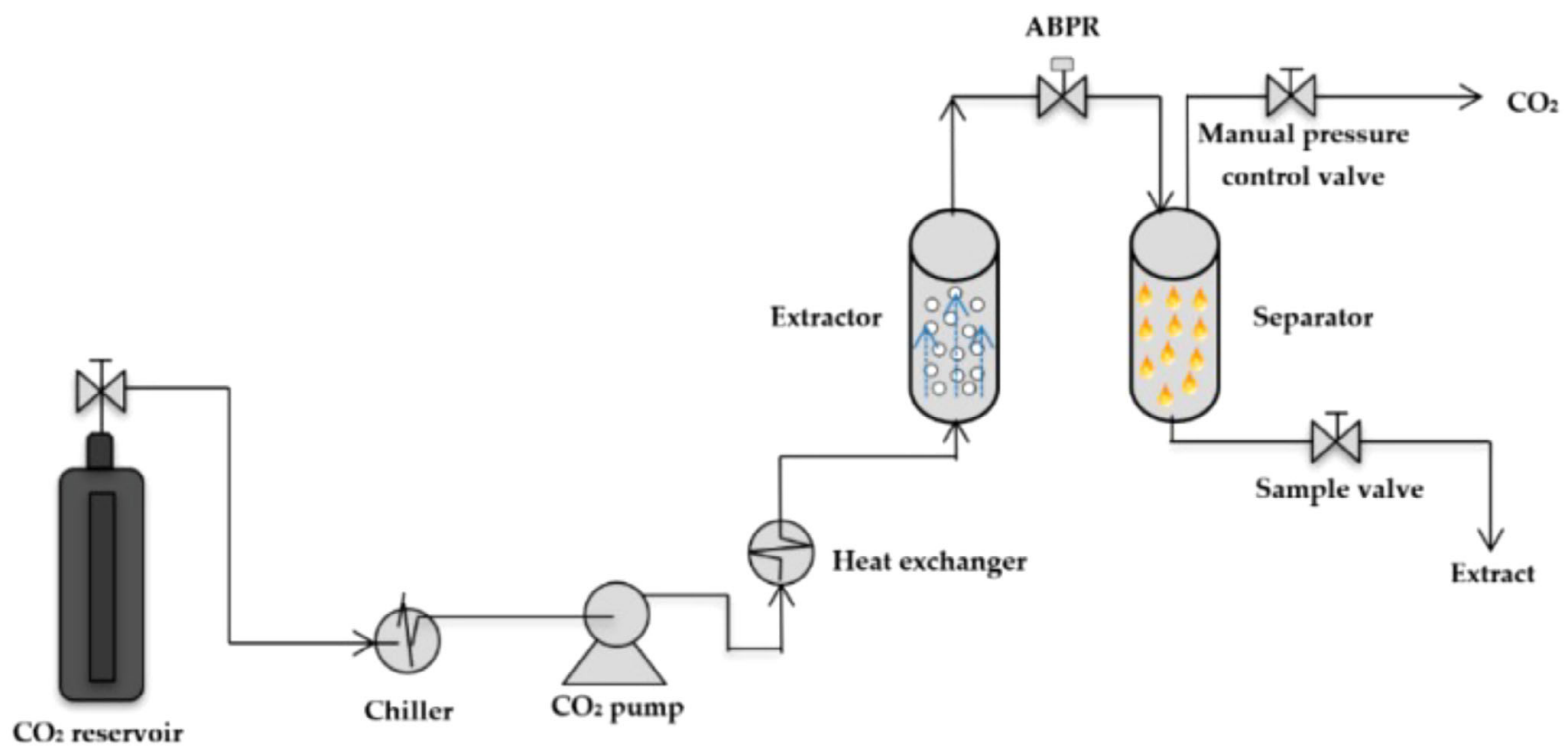
Schematic diagram of a supercritical CO_2_ extraction system, showing the CO_2_ reservoir, chiller, pump, heat exchanger, extractor vessel, separator, pressure control valve, and collection of the extract [181]. Reproduced from Ref. [181] with permission from MDPI, licensed under an open‐access Creative Commons CC‐BY license.

As in Figure 2, supercritical CO_2_ extraction is a technique where carbon dioxide is pressurized above its critical point (31.1°C and 7.38 MPa), enabling the exhibition of both gaseous and liquid properties. This supercritical CO_2_ is passed through plant material, dissolving and extracting lipophilic compounds. Once the pressure is reduced, the CO_2_ reverts to its gaseous state, leaving behind a pure extract. This method is particularly effective in extracting nonpolar or lipophilic phytochemicals such as terpenes, carotenoids, and essential oils. One of its main advantages is the production of solvent‐free extracts, as no residual solvents remain in the final product. Additionally, the process is highly selective, allowing adjustments in pressure and temperature to target specific compounds [182]. Supercritical CO_2_ extraction generally produces yields between 2 and 15% [72].

### 5.3 Steam Distillation

**FIGURE 3:**
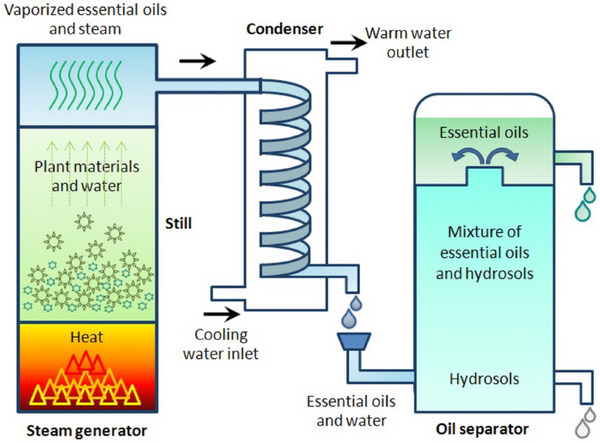
Schematic diagram of a steam distillation system for essential oil extraction, illustrating steam generation and contact with plant material in the still, condensation of volatile compounds in the condenser, and subsequent phase separation of essential oil and hydrosol in the separator [183]. Reproduced from Ref. [183] with permission from John Wiley and Sons.

As Figure 3 portrays, steam distillation involves passing steam through plant material, causing the evaporation of volatile compounds. The resulting vapors are then condensed and collected into essential oils and hydrosol. This method is ideal for extracting volatile phytochemicals such as monoterpenes, terpenoids, and aromatic compounds commonly found in flowers, leaves, and roots. It is a straightforward and widely used technique, particularly for producing essential oils, due to its simplicity and effectiveness in isolating aromatic compounds [184]. Steam distillation generally produces yields between 2.0% and 6.5% [72].

### 5.4 Cold Pressing

**FIGURE 4:**
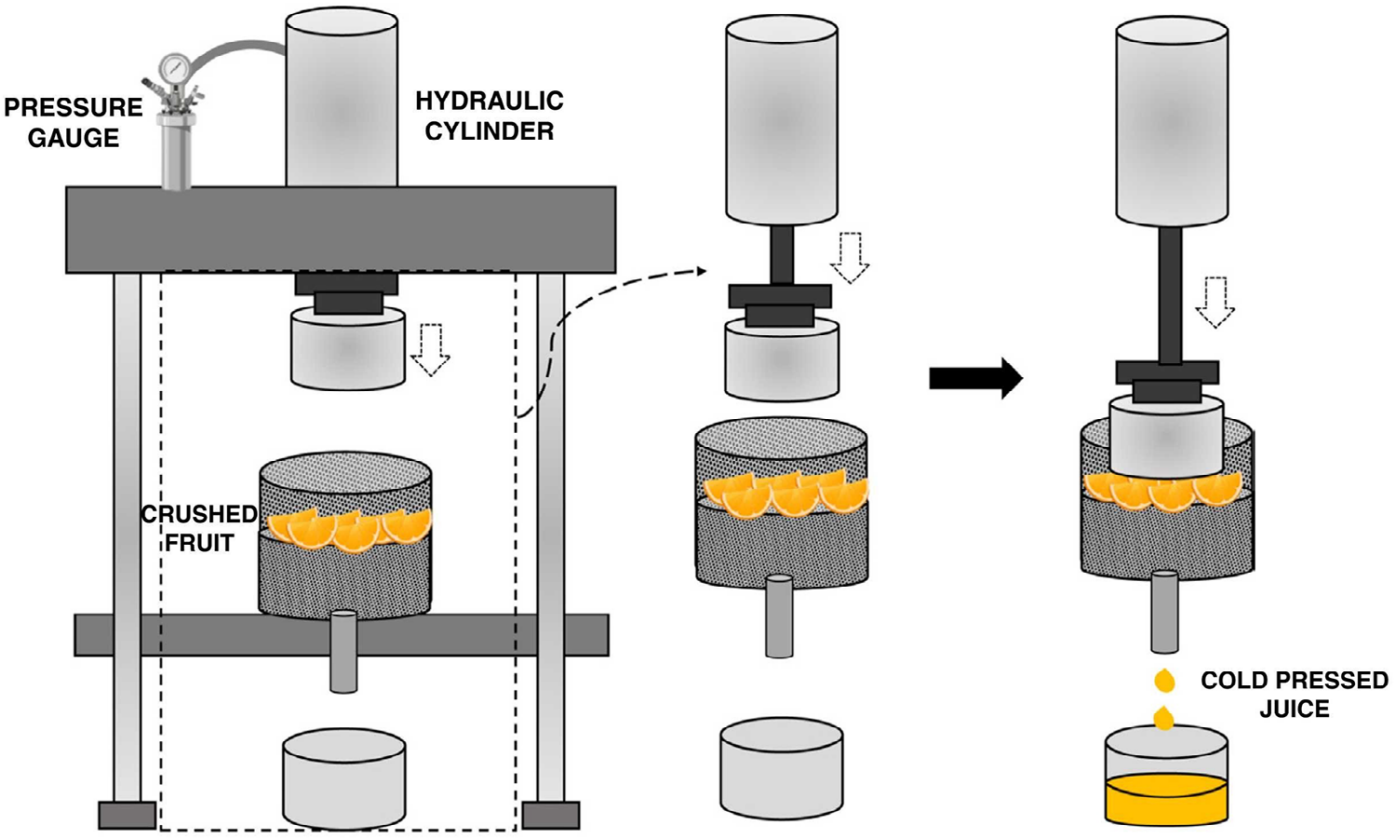
A diagrammatic representation of the cold‐pressing extraction process [185]. Reproduced from Ref. [185] with permission from Elsevier.

As illustrated in Figure 4, cold pressing is a mechanical process where plant materials, such as citrus peels or seeds, are pressed to release oils without applying heat, making it a purely physical process that does not involve solvents. This technique is primarily used for extracting fixed oils and citrus essential oils, which are rich in limonene, flavonoids, and vitamins. One of its significant advantages is that it preserves the natural integrity and bioactivity of the phytochemicals, as the absence of heat and solvents prevents degradation or alteration of the compounds [186]. Generally, cold press extraction yields around 1–5% [181].

### 5.5 Hydrodistillation

**FIGURE 5:**
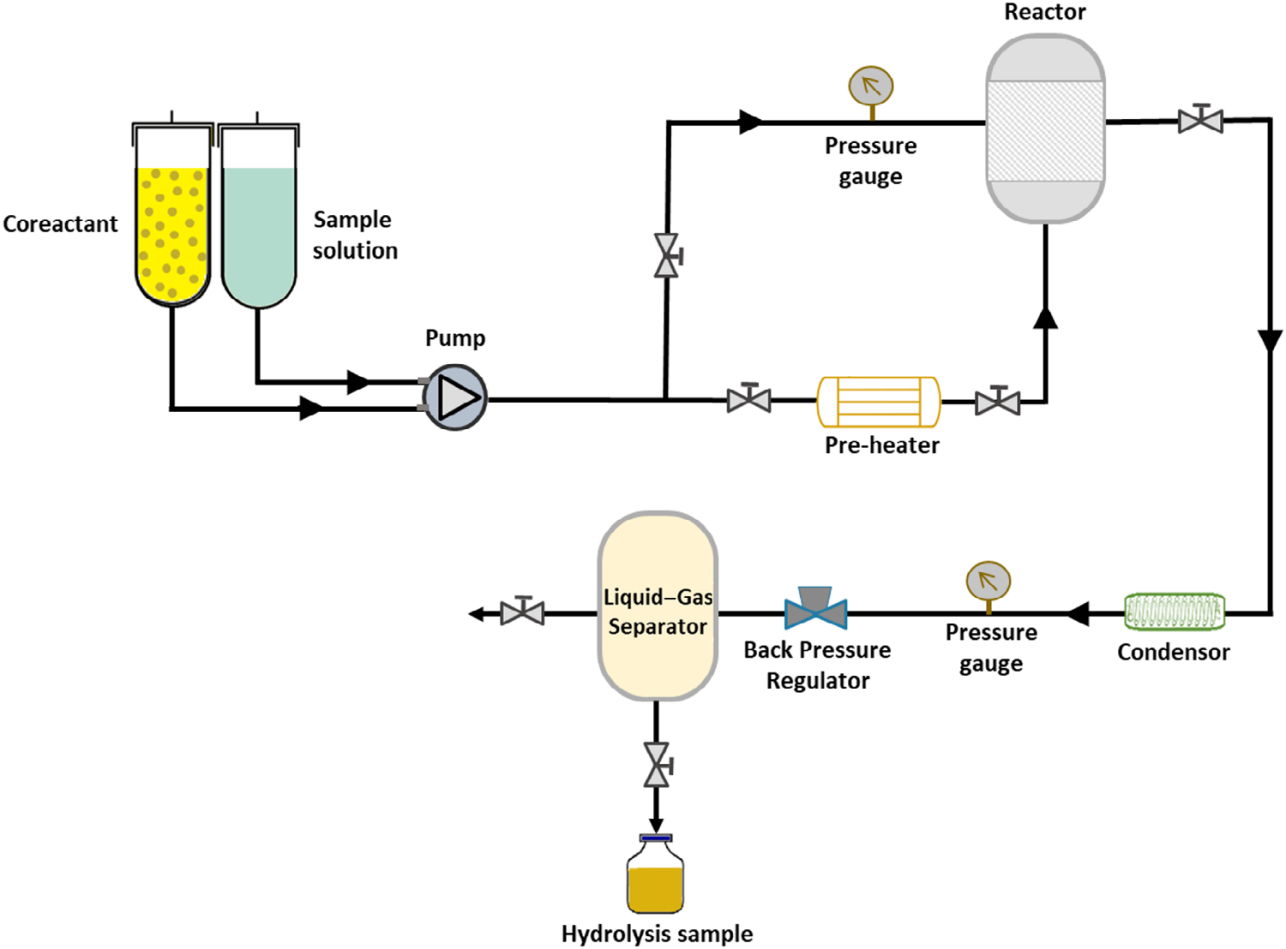
Schematic diagram of a continuous‐flow high‐pressure reactor system for hydrolysis, illustrating coreactant and sample feeds delivered by a pump through a preheater into the reactor, followed by pressure control via a back‐pressure regulator, condensation, and product collection through a liquid–gas separator [187]. Reproduced from Ref. [187] with permission from The Korean Society of Fisheries and Aquatic Science, licensed under an open access Creative Commons Attribution Non‐Commercial license.

As observed in Figure 5, hydrodistillation is a hydrolysis‐based extraction process where water or hydrolyzing agents, such as enzymes or acids, break down complex phytochemicals like glycosides or polysaccharides into simpler, bioactive forms. This method is commonly applied to extract compounds such as saponins, flavonoid glycosides, and oligosaccharides. Its primary advantage is its effectiveness in releasing bioactive compounds from complex matrices, making it particularly useful for obtaining bioavailable phytochemicals from plant materials [188]. Hydrodistillation generally produces yields of 1–5% [72].

These processes are currently the most commercially utilized extraction methods. However, there is potential for improvement as each of these processes has its unique disadvantages, ranging from solvent extraction requiring post‐extraction removal of the solvent, supercritical CO_2_ extraction inducing high costs and requiring sophisticated equipment, steam distillation's incompatibility in extracting thermally labile or nonvolatile compounds, cold pressing being limited to only certain plant materials and producing very low yields, and hydrolysis posing the risk of degradation of sensitive compounds due to the involvement of heat and acids [189].

## 6 Antibiotics and Phytochemicals

**FIGURE 6:**
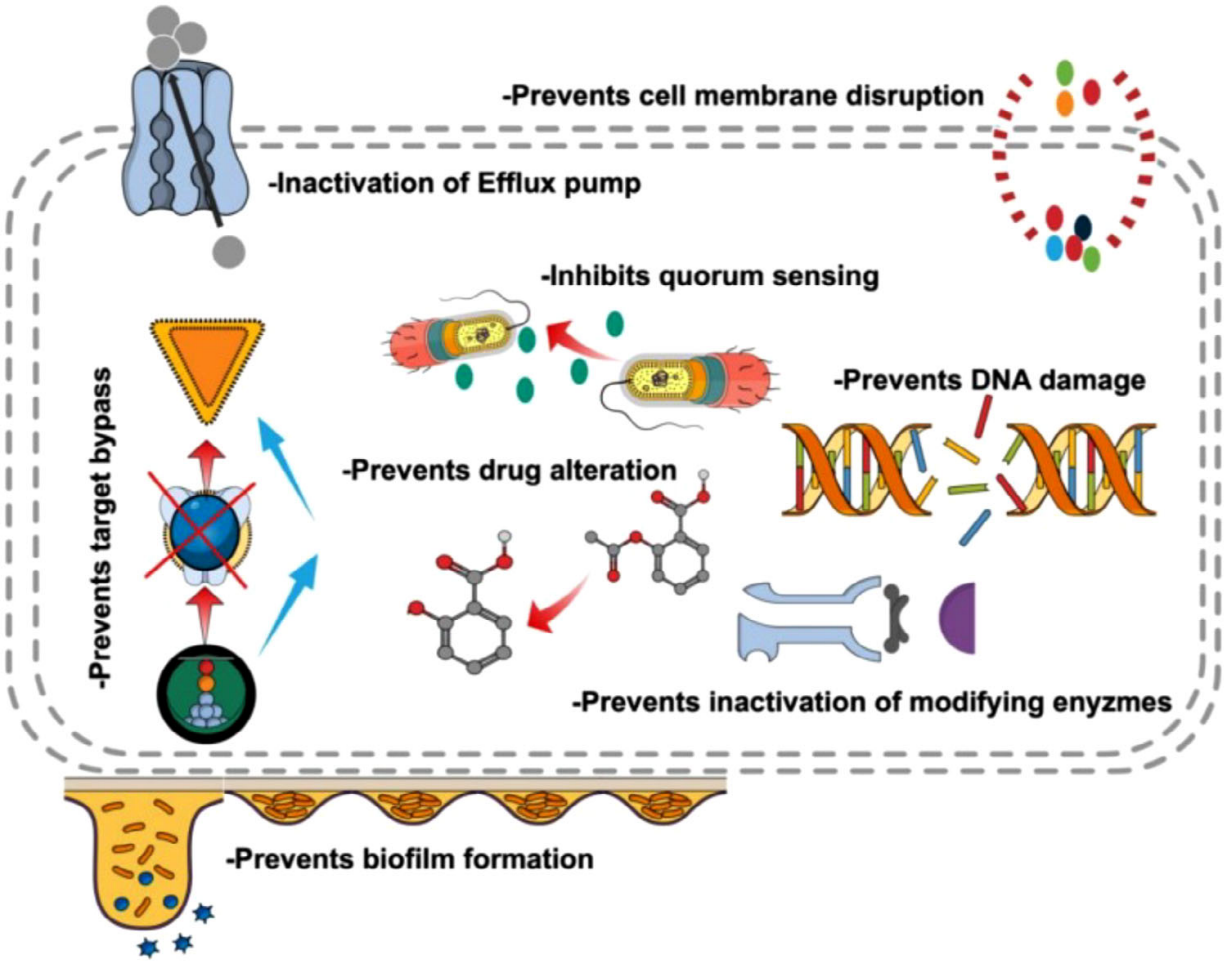
Showcases the vast array of mechanisms phytochemicals utilized to induce antimicrobial action against a wide range of pathogenic microbes [190]. Reproduced from Ref. [190] with permission from MDPI, licensed under an open access Creative Commons CC‐BY license.

Finally, natural phytochemicals from plant extracts offer a promising alternative or complement to traditional antibiotics, especially in combating microbial infections and addressing the growing challenge of antibiotic resistance. These bioactive compounds, including essential oils, alkaloids, flavonoids, phenolic acids, and terpenes, possess unique and diverse mechanisms of action that can target microbes in ways distinct from conventional antibiotics. Unlike antibiotics that typically target specific bacterial functions, such as cell wall synthesis or protein synthesis, phytochemicals often work by attacking multiple targets simultaneously. This broad‐spectrum, multitargeted approach, as illustrated in Figure 6, is a crucial advantage, as it reduces the likelihood of microbes developing resistance mutations, which is a significant problem with single‐target antibiotics. This approach facilitates the use of spice phytochemicals as alternative replacements for antibiotics and as synergistic adjuvants to antibiotics.

First, phytochemicals from spices can serve as direct antimicrobial alternatives, especially against multidrug‐resistant pathogens, owing to their broad‐spectrum and multitargeted activity. Compounds such as allicin, cinnamaldehyde, eugenol, and curcumin have been demonstrated to inhibit or eliminate various microbes, including *MRSA*, *E. coli*, and *Pseudomonas aeruginosa* [191]. Their structural diversity and ability to target multiple pathways simultaneously, such as membrane integrity, DNA replication, protein synthesis, and quorum sensing, significantly reduce the likelihood of bacteria developing resistance. This positions phytochemicals as a valuable reservoir of antimicrobial agents that could replace traditional antibiotics, especially in cases where drug resistance renders standard therapies ineffective.

While phytochemicals can serve as alternatives, their most clinically important role may be as synergistic partners with antibiotics to restore or enhance antibiotic activity against resistant bacteria. This synergy could arise through various mechanisms.

First, efflux pumps such as AcrAB–TolC in *E. coli* and MexAB–OprM in *P. aeruginosa* actively expel antibiotics, leading to treatment failure [192]. Phytochemicals like curcumin and piperine block these pumps, increasing intracellular concentrations of antibiotics. For instance, curcumin has been shown to restore tetracycline and ciprofloxacin susceptibility in resistant *E. coli* by inhibiting the activity of the pump [193]. Piperine not only inhibits efflux but also enhances the bioavailability of coadministered drugs, amplifying therapeutic outcomes [194].

Similarly, many bacteria produce enzymes that inactivate antibiotics, making treatments ineffective and leading to therapeutic failure. Phytochemicals have been shown to counteract this process by directly inhibiting these enzymes, thereby restoring antibiotic effectiveness. For example, flavonoids such as quercetin bind to and inhibit β‐lactamases, the enzymes responsible for breaking down the β‐lactam ring of penicillins and cephalosporins, which effectively re‐sensitize resistant bacteria to these drugs [195]. Likewise, phenolic acids and tannins interfere with aminoglycoside‐modifying enzymes, such as APH, AAC, and ANT, which would otherwise inactivate antibiotics like gentamicin and tobramycin by attaching chemical groups that prevent ribosomal binding. By reducing or blocking the activity of these enzymes, phytochemicals can restore the effectiveness of antibiotics that are otherwise compromised, thereby extending their clinical usefulness against resistant pathogens [196].

Another essential aspect of phytochemical synergy involves disrupting biofilms and inhibiting quorum sensing, which are significant factors in persistent and recurrent infections. Biofilms, composed of extracellular polymeric substances, form a physical and chemical barrier that limits antibiotic penetration, while quorum sensing regulates virulence factors and coordinates bacterial community behavior. Phytochemicals such as cinnamaldehyde, eugenol, and ajoene effectively interfere with these systems. For instance, cinnamaldehyde disrupts *luxS* signaling pathways and reduces biofilm biomass, while eugenol penetrates biofilms due to its lipophilic nature and destabilizes microbial membranes [197]. Ajoene specifically targets the *las* and *rhl* quorum‐sensing systems in *Pseudomonas aeruginosa*, suppressing the production of virulence factors such as elastase and rhamnolipids, which are crucial for biofilm maturation and pathogenicity [198]. By dismantling biofilms and silencing bacterial communication, these phytochemicals expose bacterial populations to antibiotics and the host's immune defenses, increasing the likelihood of successful eradication. Phytochemicals also enhance antibiotic effectiveness by lowering the MICs of traditional drugs, thereby boosting their potency against resistant strains. Carvacrol and thymol, two monoterpenes from oregano and thyme, insert into bacterial membranes, destabilize the lipid bilayer, and increase permeability. This effect sensitizes Gram‐negative bacteria such as *Klebsiella pneumoniae* and *Salmonella* to aminoglycosides, reducing their MICs by up to 16‐fold [199]. Similarly, AITC, found in mustard, disrupts bacterial detoxification pathways and increases outer membrane permeability, allowing antibiotics such as ciprofloxacin and ampicillin to reach their intracellular targets more effectively [135]. Curcumin, when combined with fluoroquinolones, not only boosts antibacterial activity but also diminishes the emergence of resistant mutants by interfering with bacterial DNA gyrase and topoisomerase IV. This dual action both extends the useful lifespan of fluoroquinolones and hinders the rapid development of resistant subpopulations, a common issue in clinical treatment [140].

Furthermore, beyond these direct antimicrobial and resistance‐modifying effects, phytochemicals can influence the host immune response, providing a dual therapeutic benefit. Flavonoids, such as quercetin and kaempferol, enhance the activity of macrophages, improve the phagocytosis of bacterial cells, and increase the secretion of cytokines, including IL‐1β and TNF‐α, which are crucial for coordinating an effective immune response [200]. Simultaneously, they help regulate overactive inflammation by scavenging ROS and downregulating proinflammatory mediators, thus preventing tissue damage during infection. This immunomodulatory effect complements antibiotic therapy by enhancing bacterial clearance while also supporting recovery in chronic or device‐associated infections, where the host immune system is often compromised [201].

Additionally, antibiotic use has declined due to increased antibiotic resistance. There is a dramatic decrease in the amount of funding invested into antibiotic research, with the most dramatic decrease occurring in public sector investments, where the funding decreases by almost $150,000,000 from 2017 to 2021 [202], highlighting the decreasing interest and the improving opportunity for phytochemicals to be an efficient alternative or synergistic enhancer for antibiotics, to assist in the combating and overcoming of pathogenic and antibiotic‐resistant microbes.

Therefore, utilizing phytochemicals together enhances antibiotic efficacy through a multipronged approach, and this layered mechanism of action not only restores the usefulness of existing antibiotics but also reduces the selective pressure that drives resistance, making phytochemicals powerful allies in the ongoing fight against multidrug‐resistant infections.

## 7 Challenges and Future Directions in the Use of Phytochemicals for Antimicrobial Therapy

### 7.1 Safety and Toxicological Considerations

However, the overuse of phytochemicals and spices can lead to significant toxicity and cytotoxicity issues, depending on the dosage and individual sensitivity. Nevertheless, it should be noted that most spices, when used at usual dosage or in daily food intake, are considered safe; however, in high doses, they can cause various toxicological effects, as shown in Table 5.

**TABLE 5:** Hazardous phytochemicals identified in common culinary spices, with associated toxicological concerns and supporting literature references.

Spice	Primary hazardous chemical	Toxicological concerns	References
Turmeric	Curcumin	Curcumin supplements linked to rare liver injury	[203]
Garlic	Allicin, organosulfur compounds	GI issues, rare hepatotoxicity, potentiates anticoagulants	[204]
Cinnamon	Coumarin (esp. Cassia)	Hepatotoxicity in sensitive individuals; EFSA tolerable daily intake 0.1 mg/kg/day	[205]
Clove	Eugenol	Acute hepatic necrosis, mucosal irritation, CNS depression	[206]
Ginger	6‐Gingerol, shogaols	GI issues, rare liver events	[207]
Black pepper	Piperine	Drug interactions (CYP3A4, P‐gp inhibition), GI irritation	[208]
Cumin	Cuminaldehyde	Airway/skin irritation, hepatotoxic potential	[209]
Cardamom	1,8‐Cineole, terpenes	Terpenes can be an irritant, and cineole can be toxic in overdoses to animals.	[210]
Bay leaf	Methyl eugenol, eugenol, cineole	Methyl eugenol is genotoxic in animals.	[211]
Nutmeg	Myristicin, safrole	Myrsiticin can cause neurotoxicity, hallucinations, arrhythmia; safrole carcinogenic in rodents	[212]
Basil	Estragole, methyl eugenol	Genotoxic/carcinogenic, liver necrosis	[213]
Mustard	AITC	Skin/airway irritant; bladder tumors and skin irritations	[214]
Fenugreek	Trigonelline, saponins	Hypoglycemia, GI issues, allergic reactions	[215]
Lemongrass	Citral, geraniol	Skin sensitizer/irritant; dermatitis	[216]
Oregano	Carvacrol, thymol	Mucosal irritation; GI issues, cytotoxicity	[217]

Table 5 highlights the various impacts of high‐dose exposure to spice phytochemicals. Some phytochemicals, such as curcumin, when consumed in excessive doses (above 8 g/day), may cause gastrointestinal problems, including diarrhea and bloating [218], with chronically high doses potentially causing oxidative stress in healthy cells. Similarly, eugenol is hepatotoxic at high concentrations, and cases of eugenol poisoning have been reported as caused primarily by the ingestion of undiluted clove oil, resulting in acute liver failure and abdominal pain [219]. Similarly, in animal studies, 5 g or more of safrole found in nutmeg and cinnamon has been linked to liver damage and carcinogenicity, with excessive nutmeg intake leading to nutmeg poisoning, which is characterized by liver damage, nausea and even hallucinations due to the neurotoxic effects of myristicin, another compound in nutmeg [220]. Moreover, coumarin, a phytochemical primarily found in *Cinnamomum cassia*, has been banned by the United States Food and Drug Administration since 1954 due to proven hepatoxicity concerns [221]. Therefore, these examples underscore the importance of moderation and careful regulation of spices and phytochemicals because they offer immense health benefits. Still, overuse or improper handling can lead to severe toxicological consequences.

### 7.2 Challenges With Integration Into Mainstream Healthcare

The application of phytochemicals as antimicrobials, either as standalone therapies or as synergistic adjuvants with conventional antibiotics, faces significant challenges before they can be integrated into routine clinical practice. One of the foremost barriers lies in the regulatory landscape. For adoption into health systems such as the NHS, phytochemical‐based therapies must undergo rigorous evaluation by bodies like NICE in the UK, which requires robust, large‐scale clinical trial data on safety, efficacy, pharmacokinetics, and cost effectiveness [222]. Currently, most evidence supporting the use of phytochemicals is preclinical or limited to small‐scale studies, indicating that substantial translational gaps remain. Similarly, another major obstacle concerning the integration of phytochemicals into mainstream healthcare is the issue of standardization and quality control. Phytochemicals are derived from natural plant sources, which are subject to variability due to factors such as climate, soil composition, cultivation practices, and harvest timing. These factors make it challenging to ensure consistent concentrations of active compounds. Seasonal dependency and geographical variation in spice production further complicate large‐scale procurement [223]. For use in healthcare and to ensure efficacy and safety, extracts must practice and adhere to good plant authentication and identification practice, good agricultural and collection practice, good manufacturing practice, and good laboratory practice with strict quality assurance to prevent contamination and to guarantee reproducibility [224]. This introduces substantial costs and logistical hurdles. Additionally, phytochemicals face pharmacological limitations, including poor solubility, stability, and rapid metabolism in the human body, which reduce their bioavailability and therapeutic efficacy. For example, curcumin is well known for its potent antimicrobial and anti‐inflammatory properties, but its oral bioavailability is extremely low due to poor absorption and rapid hepatic metabolism [225]. Without delivery systems such as nanoparticles, liposomes, or chemical derivatization, many promising phytochemicals fail to achieve therapeutic plasma concentrations. Furthermore, there is limited understanding of their long‐term safety profiles and potential for adverse interactions with antibiotics or other drugs, which may delay regulatory approval.

### 7.3 Future Directions to Improve Phytochemical Use

In the near future, several technological and strategic innovations could help overcome these barriers and challenges, such as advancements in nanotechnology, which offer promising solutions to poor solubility and bioavailability by encapsulating phytochemicals in nanoemulsions [226], lipid‐based carriers, polymeric nanoparticles, and thereby enhancing absorption, stability, and targeted delivery [227]. Similarly, synthetic biology and metabolic engineering could enable the large‐scale biosynthesis of phytochemicals in controlled microbial or plant cell factories, reducing reliance on seasonal crops and ensuring a consistent supply [228].

Furthermore, using artificial intelligence (AI) and machine learning in drug discovery can accelerate the identification of new phytochemicals, predict their molecular targets, and design optimized phytochemical‐antibiotic combinations that improve synergy while decreasing toxicity. AI‐based modeling might also forecast resistance patterns, supporting the strategic deployment of phytochemicals alongside existing antibiotics [229]. Even more advanced, quantum biomedicine, which applies quantum chemistry and physics to examine biomolecular interactions at an unmatched level of detail, would enable the early detection of antimicrobial resistance mutations and predict microbial responses to phytochemicals before resistance arises [230]. Last, challenges related to acceptance and education must also be addressed. Clinicians and patients alike may view phytochemicals as “natural remedies” rather than rigorously tested medicines, which can lead to either overenthusiastic use without proper dosing or skepticism regarding their clinical validity. Overcoming this will require various high quality, randomized controlled trials, regulatory clarity, and public health communication that highlights their evidence base [231], thereby facilitating and smoothing the integration of phytochemical therapy into mainstream and commercial healthcare.

## 8 Conclusions

Phytochemicals from plant extracts offer a promising and innovative approach to combating antibiotic resistance, potentially playing a vital role in the future of infectious disease treatment. These compounds provide broad‐spectrum antimicrobial activity by disrupting multiple microbial processes, including cell membrane integrity, enzyme function, and DNA replication, while also overcoming resistance mechanisms such as efflux pumps and biofilm formation. Their ability to work synergistically with traditional antibiotics enhances treatment efficacy and helps reduce the selective pressure that drives the emergence of resistant strains. In the future, continued advanced research into phytochemicals can help identify and optimize their use as unique or complementary agents in combination with antibiotics to eradicate infections and cure diseases. This prospective work is critical in developing natural alternatives to antibiotics, particularly in addressing the growing threat of multidrug‐resistant infections. As resistance to conventional drugs increases, integrating phytochemicals into clinical practice could significantly enhance our ability to manage and treat diseases more effectively, offering a sustainable and long‐term solution in the ongoing fight against antimicrobial resistance.

## Author Contributions

H.B. De Silva conducted the literature search and drafted the manuscript. Yanqi Dai, Shervanthi Homer‐Vanniasinkam, and Mohan Edirisinghe contributed by providing additional information, critically reviewing the content, and editing the manuscript for intellectual clarity and accuracy. All authors have read and accepted the final version of the manuscript.

## Conflicts of Interest

Author Mohan Edirisinghe is an Editorial Board member of Medcomm. Author Mohan Edirisinghe was not involved in the journal's review of or decisions related to this manuscript. HDDES Extracts PVT. Ltd, Sri Lanka, sponsors and supports the doctoral studies of H.B. De Silva. The other authors declared no conflicts of interest.

## Data Availability

The authors have nothing to report.

## References

[1] M. A. Salam , M. Y. Al‐Amin , M. T. Salam , et al., “Antimicrobial Resistance: A Growing Serious Threat for Global Public Health,” Healthcare 11 (2023): 1946. 37444780 10.3390/healthcare11131946 PMC10340576

[2] C. S. Ho , C. T. H. Wong , T. T. Aung , et al., “Antimicrobial Resistance: A Concise Update,” The Lancet Microbe 6 (2024): 100947. 39305919 10.1016/j.lanmic.2024.07.010

[3] L. Diniz do Nascimento , A. A. B. de Moraes , K. S. da Costa , et al., “Bioactive Natural Compounds and Antioxidant Activity of Essential Oils from Spice Plants: New Findings and Potential Applications,” Biomolecules 10 (2020): 988. 32630297 10.3390/biom10070988 PMC7407208

[4] P. Khatri , A. Rani , S. Hameed , S. Chandra , C. M. Chang , and R. P. Pandey , “Current Understanding of the Molecular Basis of Spices for the Development of Potential Antimicrobial Medicine,” Antibiotics 12 (2023): 270. 36830181 10.3390/antibiotics12020270 PMC9952367

[5] K. E. Vivekanandan , P. V. Kumar , R. C. Jaysree , and T. Rajeshwari , “Exploring Molecular Mechanisms of Drug Resistance in Bacteria and Progressions in CRISPR/Cas9‐Based Genome Expurgation Solutions,” Global Medical Genetics 12, no. 2 (2025): 100042. 40051841 10.1016/j.gmg.2025.100042 PMC11883354

[6] K. Bush and P. A. Bradford , “β‐Lactams and β‐Lactamase Inhibitors: An Overview,” Cold Spring Harbor Perspectives in Medicine 6 (2016): a025247. 27329032 10.1101/cshperspect.a025247 PMC4968164

[7] C. L. Tooke , P. Hinchliffe , E. C. Bragginton , et al., “β‐Lactamases and β‐Lactamase Inhibitors in the 21st Century,” Journal of Molecular Biology 431 (2019): 3472–3500. 30959050 10.1016/j.jmb.2019.04.002 PMC6723624

[8] G. Alvisi , A. Curtoni , R. Fonnesu , et al., “Epidemiology and Genetic Traits of Carbapenemase‐Producing Enterobacterales: A Global Threat to Human Health,” Antibiotics 14 (2025): 141–141. 40001385 10.3390/antibiotics14020141 PMC11852015

[9] M. Mora‐Ochomogo and C. T. Lohans , “β‐Lactam Antibiotic Targets and Resistance Mechanisms: From Covalent Inhibitors to Substrates,” RSC Medicinal Chemistry 12 (2021): 1623–1639. 34778765 10.1039/d1md00200g PMC8528271

[10] D. Brdová , T. Ruml , and J. Viktorová , “Mechanism of Staphylococcal Resistance to Clinically Relevant Antibiotics,” Drug Resistance Updates 77 (2024): 101147–101147. 39236354 10.1016/j.drup.2024.101147

[11] C. Sibold , J. Henrichsen , A. König , C. Martin , L. Chalkley , and R. Hakenbeck , “Mosaic*pbpX*genes of Major Clones of Penicillin‐resistant Streptococcus*pneumoniae*have Evolved from*pbpX*genes of a Penicillin‐Sensitive *Streptococcus Oralis* ,” Molecular Microbiology 12 (1994): 1013–1023. 7934893 10.1111/j.1365-2958.1994.tb01089.x

[12] D. Zeng , D. Debabov , T. L. Hartsell , et al., “Approved Glycopeptide Antibacterial Drugs: Mechanism of Action and Resistance,” Cold Spring Harbor Perspectives in Medicine 6 (2016): a026989. 27663982 10.1101/cshperspect.a026989 PMC5131748

[13] J. Xie , J. G. Pierce , R. C. James , A. Okano , and D. L. Boger , “A Redesigned Vancomycin Engineered for Duald‐Ala‐d‐Ala andd‐Ala‐d‐Lac Binding Exhibits Potent Antimicrobial Activity Against Vancomycin‐Resistant Bacteria,” Journal of the American Chemical Society 133 (2011): 13946–13949. 21823662 10.1021/ja207142h PMC3164945

[14] C. M. Hill , K. M. Krause , S. R. Lewis , et al., “Specificity of Induction of the vanA and vanB Operons in Vancomycin‐Resistant Enterococci by Telavancin,” Antimicrobial Agents and Chemotherapy 54 (2010): 2814–2818. 20404117 10.1128/AAC.01737-09 PMC2897282

[15] K. M. Krause , A. W. Serio , T. R. Kane , and L. E. Connolly , “Aminoglycosides: An Overview,” Cold Spring Harbor Perspectives in Medicine 6 (2016): a027029. 27252397 10.1101/cshperspect.a027029 PMC4888811

[16] M. S. Ramirez , N. Nikolaidis , and M. E. Tolmasky , “Rise and Dissemination of Aminoglycoside Resistance: The Aac(6′)‐Ib Paradigm,” Frontiers in Microbiology 4 (2013): 121. 23730301 10.3389/fmicb.2013.00121 PMC3656343

[17] M. S. Ramirez and M. E. Tolmasky , “Aminoglycoside Modifying Enzymes,” Drug Resistance Updates 13 (2010): 151–171. 20833577 10.1016/j.drup.2010.08.003 PMC2992599

[18] S. R. Connell , D. M. Tracz , K. H. Nierhaus , and D. E. Taylor , “Ribosomal Protection Proteins and Their Mechanism of Tetracycline Resistance,” Antimicrobial Agents and Chemotherapy 47 (2003): 3675–3681. 14638464 10.1128/AAC.47.12.3675-3681.2003 PMC296194

[19] T. Tenson , M. Lovmar , and M. Ehrenberg , “The Mechanism of Action of Macrolides, Lincosamides and Streptogramin B Reveals the Nascent Peptide Exit Path in the Ribosome,” Journal of Molecular Biology 330 (2003): 1005–1014. 12860123 10.1016/s0022-2836(03)00662-4

[20] M. S. Svetlov , E. A. Syroegin , E. V. Aleksandrova , et al., “Structure of Erm‐modified 70S Ribosome Reveals the Mechanism of Macrolide Resistance,” Nature Chemical Biology 17 (2021): 412–420. 33462493 10.1038/s41589-020-00715-0 PMC7990689

[21] K. S. Long and B. Vester , “Resistance to Linezolid Caused by Modifications at Its Binding Site on the Ribosome,” Antimicrobial Agents and Chemotherapy 56 (2011): 603–612. 22143525 10.1128/AAC.05702-11 PMC3264260

[22] P. Gupta , S. Sothiselvam , N. Vázquez‐Laslop , and A. S. Mankin , “Deregulation of Translation due to Post‐transcriptional Modification of rRNA Explains Why Erm Genes Are Inducible,” Nature Communications 4 (2013): 1984. 10.1038/ncomms2984 23749080

[23] H. Chen , W. Wu , M. Ni , et al., “Linezolid‐resistant Clinical Isolates of Enterococci and Staphylococcus Cohnii from a Multicentre Study in China: Molecular Epidemiology and Resistance Mechanisms,” International Journal of Antimicrobial Agents 42 (2013): 317–321. 23880167 10.1016/j.ijantimicag.2013.06.008

[24] D. C. Hooper and G. A. Jacoby , “Topoisomerase Inhibitors: Fluoroquinolone Mechanisms of Action and Resistance,” Cold Spring Harbor Perspectives in Medicine 6 (2016): a025320. 27449972 10.1101/cshperspect.a025320 PMC5008060

[25] G. A. Jacoby , J. Strahilevitz , and D. C. Hooper , “Plasmid‐Mediated Quinolone Resistance,” Microbiology Spectrum 2 (2014), 10.1128/microbiolspec.plas-0006-2013. PMC4288778 25584197

[26] M. Li , L. J. Chao , Y. Lu , et al., “rpoB Mutations and Effects on Rifampin Resistance in Mycobacterium Tuberculosis,” Infection and Drug Resistance 14 (2021): 4119–4128. 34675557 10.2147/IDR.S333433 PMC8502021

[27] M. Kiani , A. Astani , G. Eslami , et al., “Upstream Region of OprD Mutations in Imipenem‐resistant and Imipenem‐sensitive Pseudomonas Isolates,” AMB Express 11 (2021): 82. 34089411 10.1186/s13568-021-01243-3 PMC8179858

[28] C. Bornet , N. Saint , L. Fetnaci , et al., “Omp35, a New *Enterobacter aerogenes* Porin Involved in Selective Susceptibility to Cephalosporins,” Antimicrobial Agents and Chemotherapy 48 (2004): 2153–2158. 15155215 10.1128/AAC.48.6.2153-2158.2004 PMC415628

[29] M. Masi , J. Vergalli , I. Ghai , et al., “Cephalosporin Translocation across Enterobacterial OmpF and OmpC Channels, a Filter across the Outer Membrane,” Communications Biology 5 (2022): 1059. 36198902 10.1038/s42003-022-04035-y PMC9534850

[30] J. Anes , M. P. McCusker , S. Fanning , and M. Martins , “The Ins and Outs of RND Efflux Pumps in Escherichia coli,” Frontiers in Microbiology 6 (2015): 587. 26113845 10.3389/fmicb.2015.00587 PMC4462101

[31] E. W. Yu , J. R. Aires , and H. Nikaido , “AcrB Multidrug Efflux Pump of Escherichia coli: Composite Substrate‐Binding Cavity of Exceptional Flexibility Generates Its Extremely Wide Substrate Specificity,” Journal of Bacteriology 185 (2003): 5657–5664. 13129936 10.1128/JB.185.19.5657-5664.2003 PMC193975

[32] A. B. Lorusso , J. A. Carrara , C. D. N. Barroso , F. F. Tuon , and H. Faoro , “Role of Efflux Pumps on Antimicrobial Resistance in Pseudomonas aeruginosa,” International Journal of Molecular Sciences 23 (2022): 15779. 36555423 10.3390/ijms232415779 PMC9779380

[33] M. Sánchez‐Osuna , P. Cortés , J. Barbé , and I. Erill , “Origin of the Mobile Di‐Hydro‐Pteroate Synthase Gene Determining Sulfonamide Resistance in Clinical Isolates,” Frontiers in Microbiology 9 (2019): 3332. 30687297 10.3389/fmicb.2018.03332 PMC6335563

[34] S. M. Reeve , D. Si , J. Krucinska , et al., “Toward Broad Spectrum Dihydrofolate Reductase Inhibitors Targeting Trimethoprim Resistant Enzymes Identified in Clinical Isolates of Methicillin Resistant Staphylococcus aureus,” ACS Infectious Diseases 5 (2019): 1896–1906. 31565920 10.1021/acsinfecdis.9b00222 PMC7025792

[35] M. Venkatesan , M. Fruci , L. A. Verellen , et al., “Molecular Mechanism of Plasmid‐borne Resistance to Sulfonamide Antibiotics,” Nature Communications 14 (2023): 4031. 10.1038/s41467-023-39778-7 PMC10328974 37419898

[36] F. F. Andrade , D. Silva , A. Rodrigues , and C. Pina‐Vaz , “Colistin Update on Its Mechanism of Action and Resistance, Present and Future Challenges,” Microorganisms 8 (2020): 1716. 33147701 10.3390/microorganisms8111716 PMC7692639

[37] D. Lakshmanan , D. Ramasamy , V. Subramanyam , and S. Suresh , “Mobile Colistin Resistance (*mcr*) Genes and Recent Developments in Colistin Resistance Detection,” Letters in Applied Microbiology 76 (2023): ovad102. 37673673 10.1093/lambio/ovad102

[38] P. Hinchliffe , Q. E. Yang , E. Portal , et al., “Insights into the Mechanistic Basis of Plasmid‐Mediated Colistin Resistance from Crystal Structures of the Catalytic Domain of MCR‐1,” Scientific Reports 7 (2017): 39392. 28059088 10.1038/srep39392 PMC5216409

[39] A. M. S. Shein , D. L. Wannigama , P. G. Higgins , et al., “High Prevalence of mgrB‐mediated Colistin Resistance among Carbapenem‐resistant Klebsiella pneumoniae Is Associated with Biofilm Formation, and Can be Overcome by Colistin‐EDTA Combination Therapy,” Scientific Reports 12 (2022): 12939. 35902639 10.1038/s41598-022-17083-5 PMC9334626

[40] H. Y. Liu , E. L. Prentice , and M. A. Webber , “Mechanisms of Antimicrobial Resistance in Biofilms,” Npj Antimicrobials and Resistance 2 (2024): 27. 39364333 10.1038/s44259-024-00046-3 PMC11445061

[41] S. Sharma , J. Mohler , S. D. Mahajan , S. A. Schwartz , L. Bruggemann , and R. Aalinkeel , “Microbial Biofilm: A Review on Formation, Infection, Antibiotic Resistance, Control Measures, and Innovative Treatment,” Microorganisms 11 (2023): 1614. 37375116 10.3390/microorganisms11061614 PMC10305407

[42] L. R. Mulcahy , J. L. Burns , S. Lory , and K. Lewis , “Emergence of Pseudomonas aeruginosa Strains Producing High Levels of Persister Cells in Patients with Cystic Fibrosis,” Journal of Bacteriology 192 (2010): 6191–6199. 20935098 10.1128/JB.01651-09 PMC2981199

[43] S. Yang , I. D. Hay , D. R. Cameron , et al., “Antibiotic Regimen Based on Population Analysis of Residing Persister Cells Eradicates Staphylococcus epidermidis Biofilms,” Scientific Reports 5 (2015): 18578. 26687035 10.1038/srep18578 PMC4685274

[44] K. W. K. Tang , B. C. Millar , and J. E. Moore , “Antimicrobial Resistance (AMR),” British Journal of Biomedical Science 80 (2023): 11387. 37448857 10.3389/bjbs.2023.11387 PMC10336207

[45] K. A. Cohen , A. L. Manson , C. A. Desjardins , T. Abeel , and A. M. Earl , “Deciphering Drug Resistance in Mycobacterium Tuberculosis Using Whole‐genome Sequencing: Progress, Promise, and Challenges,” Genome Medicine 11 (2019): 45. 31345251 10.1186/s13073-019-0660-8 PMC6657377

[46] S. Wang , J. Yao , B. Zhou , et al., “Bacteriostatic Effect of Quercetin as an Antibiotic Alternative in Vivo and Its Antibacterial Mechanism in Vitro,” Journal of Food Protection 81, no. 1 (2018): 68–78. 29271686 10.4315/0362-028X.JFP-17-214

[47] R. Hakenbeck , “Mosaic Genes and Their Role in Penicillin‐resistant *Streptococcus pneumoniae* (minireview),” Electrophoresis 19 (1998): 597–601. 9588809 10.1002/elps.1150190423

[48] C. R. Scharn , F. C. Tenover , and R. V. Goering , “Transduction of Staphylococcal Cassette ChromosomemecElements between Strains of Staphylococcus aureus,” Antimicrobial Agents and Chemotherapy 57 (2013): 5233–5238. 23939891 10.1128/AAC.01058-13 PMC3811280

[49] K. A. Moser , L. Zhang , I. Spicknall , et al., “The Role of Mobile Genetic Elements in the Spread of Antimicrobial‐Resistant Escherichia coli From Chickens to Humans in Small‐Scale Production Poultry Operations in Rural Ecuador,” American Journal of Epidemiology 187 (2017): 558–567. 10.1093/aje/kwx286 PMC5860545 29506196

[50] F. Prestinaci , P. Pezzotti , and A. Pantosti , “Antimicrobial Resistance: A Global Multifaceted Phenomenon,” Pathogens and Global Health 109 (2015): 309–318. 26343252 10.1179/2047773215Y.0000000030 PMC4768623

[51] N. Dosoky and W. Setzer , “Chemical Composition and Biological Activities of Essential Oils of Curcuma Species,” Nutrients 10, no. 9 (2018): 1196. 30200410 10.3390/nu10091196 PMC6164907

[52] S. G. Santhosha , P. Jamuna , and S. N. Prabhavathi , “Bioactive Components of Garlic and Their Physiological Role in Health Maintenance: A Review,” Food Bioscience 3 (2013): 59–74.

[53] H. H. Phu , K. Pham Van , T. H. Tran , and D. T. N. Pham , “Extraction, Chemical Compositions and Biological Activities of Essential Oils of Cinnamomum Verum Cultivated in Vietnam,” Processes 10 (2022): 1713.

[54] A. Hazim , D. N. Rizkiyah , L. Qomariyah , I. Irianto , and N. R. Mohd Putra , “Unlocking the Full Potential of Clove (Syzygium aromaticum) Spice: An Overview of Extraction Techniques, Bioactivity, and Future Opportunities in the Food and Beverage Industry,” Processes 11 (2023): 2453–2453.

[55] W. Hu , A. Yu , S. Wang , et al., “Extraction, Purification, Structural Characteristics, Biological Activities, and Applications of the Polysaccharides from Zingiber Officinale Roscoe. (Ginger): A Review,” Molecules (Basel, Switzerland) 28 (2023): 3855. 37175266 10.3390/molecules28093855 PMC10179780

[56] A. Milenković and L. Stanojević , “Black Pepper: Chemical Composition and Biological Activities,” Advanced Technologies 10 (2021): 40–50.

[57] O. Merah , B. Sayed‐Ahmad , T. Talou , et al., “Biochemical Composition of Cumin Seeds, and Biorefining Study,” Biomolecules 10 (2020): 1054. 32679821 10.3390/biom10071054 PMC7407589

[58] Abdullah , A. Asghar , M. S. Butt , M. Shahid , and Q. Huang , “Evaluating the Antimicrobial Potential of Green Cardamom Essential Oil Focusing on Quorum Sensing Inhibition of Chromobacterium Violaceum,” Journal of Food Science and Technology 54, no. 8 (2017): 2306–2315. 28740287 10.1007/s13197-017-2668-7 PMC5502022

[59] L. Hartanti , S. M. K. Yonas , J. J. Mustamu , S. Wijaya , H. K. Setiawan , and L. Soegianto , “Influence of Extraction Methods of Bay Leaves (Syzygium polyanthum) on Antioxidant and HMG‐CoA Reductase Inhibitory Activity,” Heliyon 5 (2019): e01485. 31008409 10.1016/j.heliyon.2019.e01485 PMC6458466

[60] K. Ashokkumar , J. Simal‐Gandara , M. Murugan , M. K. Dhanya , and A. Pandian , “Nutmeg (Myristica fragrans Houtt.) Essential Oil: A Review on Its Composition, Biological, and Pharmacological Activities,” Phytotherapy Research 36 (2022): 2839–2851. 35567294 10.1002/ptr.7491 PMC9541156

[61] M. Ferreira , D. H. Kringel , D. de , R. Zavareze , and A. Renato , “Basil Essential Oil: Methods of Extraction, Chemical Composition, Biological Activities, and Food Applications,” Food and Bioprocess Technology 15 (2021): 1–27.

[62] J. Lietzow , “Biologically Active Compounds in Mustard Seeds: A Toxicological Perspective,” Foods 10 (2021): 2089. 34574199 10.3390/foods10092089 PMC8472142

[63] S. Akbari , N. H. Abdurahman , R. M. Yunus , O. R. Alara , and O. O. Abayomi , “Extraction, Characterization and Antioxidant Activity of Fenugreek (Trigonella‐Foenum Graecum) Seed Oil,” Materials Science for Energy Technologies 2 (2019): 349–355.

[64] B. Ashaq , K. Rasool , S. Habib , et al., “Insights into Chemistry, Extraction and Industrial Application of Lemon Grass Essential Oil ‐A Review of Recent Advances,” Food Chemistry: X 22 (2024): 101521. 38952570 10.1016/j.fochx.2024.101521 PMC11215000

[65] S. Soltani , A. Shakeri , M. Iranshahi , and M. Boozari , “A Review of the Phytochemistry and Antimicrobial Properties of Origanum vulgare L. and Subspecies,” Iranian Journal of Pharmaceutical Research IJPR 20 (2021): 268–285. 34567161 10.22037/ijpr.2020.113874.14539 PMC8457725

[66] A. Amalraj , A. Pius , S. Gopi , and S. Gopi , “Biological Activities of Curcuminoids, Other Biomolecules from Turmeric and Their Derivatives—A Review,” Journal of Traditional and Complementary Medicine 7 (2017): 205–233. 28417091 10.1016/j.jtcme.2016.05.005 PMC5388087

[67] A. Shang , S. Cao , X. Xu , et al., “Bioactive Compounds and Biological Functions of Garlic (Allium sativum L.),” Foods 8 (2019): 246. 31284512 10.3390/foods8070246 PMC6678835

[68] D. Kurnia , D. Ajiati , L. Heliawati , and D. Sumiarsa , “Antioxidant Properties and Structure‐Antioxidant Activity Relationship of Allium Species Leaves,” Molecules (Basel, Switzerland) 26 (2021): 7175. 34885755 10.3390/molecules26237175 PMC8659087

[69] P. Songvut , W. Nakareangrit , W. Cholpraipimolrat , et al., “Unraveling the Interconversion Pharmacokinetics and Oral Bioavailability of the Major Ginger Constituents: [6]‐gingerol, [6]‐shogaol, and Zingerone after Single‐dose Administration in Rats,” Frontiers in Pharmacology 15 (2024): 1391019. 38904001 10.3389/fphar.2024.1391019 PMC11187260

[70] S. Banerjee , P. Katiyar , V. Kumar , et al., “Black Pepper and Piperine Induce Anticancer Effects on Leukemia Cell Line,” Toxicology Research 10 (2021): 169–182. 33884168 10.1093/toxres/tfab001 PMC8045589

[71] F. S. Mohammed , M. Sevindik , İ. Uysal , C. Çesko , and H. Koraqi , “Chemical Composition, Biological Activities, Uses, Nutritional and Mineral Contents of Cumin (Cuminum cyminum),” Measurement: Food 14 (2024): 100157.

[72] N. Abdullah , N. Ahmad , T. Wenni , et al., “Recent Advances in the Extraction, Chemical Composition, Therapeutic Potential, and Delivery of Cardamom Phytochemicals,” Frontiers in Nutrition 9 (2022): 1024820. 36245491 10.3389/fnut.2022.1024820 PMC9562589

[73] S. Batool , R. A. Khera , M. A. Hanif , and M. A. Ayub , “Bay Leaf,” Medicinal Plants of South Asia (2020): 63–74, 10.1016/B978-0-08-102659-5.00005-7.

[74] M. E. Götz , B. Sachse , B. Schäfer , and A. Eisenreich , “Myristicin and Elemicin: Potentially Toxic Alkenylbenzenes in Food,” Foods 11 (2022): 1988. 35804802 10.3390/foods11131988 PMC9265716

[75] R. Joshi , “Chemical Composition and Antimicrobial Activity of the Essential Oil of Ocimum Basilicum L. (sweet basil) From Western Ghats of North West Karnataka, India,” Ancient Science of Life 33 (2014): 149. 10.4103/0257-7941.144618 PMC4264302 25538349

[76] J. Gerendás , J. Podestát , T. Stahl , et al., “Interactive Effects of Sulfur and Nitrogen Supply on the Concentration of Sinigrin and Allyl Isothiocyanate in Indian Mustard (Brassica juncea L.),” Journal of Agricultural and Food Chemistry 57 (2009): 3837–3844. 19309148 10.1021/jf803636h

[77] T. Visuvanathan , L. T. L. Than , J. Stanslas , S. Y. Chew , and S. Vellasamy , “Revisiting Trigonella Foenum‐graecum L.: Pharmacology and Therapeutic Potentialities,” Plants 11 (2022): 1450. 35684222 10.3390/plants11111450 PMC9182856

[78] A. A. A. Murtadlo , A. N. M. Ansori , V. D. Kharisma , et al., “A Mini Review of Curcuma Longa: Antimicrobial Properties,” Journal of Medicinal and Chemical Sciences 7, no. 1 (2023): 215–221.

[79] K. Priyadarsini , “The Chemistry of Curcumin: From Extraction to Therapeutic Agent,” Molecules (Basel, Switzerland) 19, no. 12 (2014): 20091–20112. 25470276 10.3390/molecules191220091 PMC6270789

[80] C. Dai , J. Lin , H. Li , et al., “The Natural Product Curcumin as an Antibacterial Agent: Current Achievements and Problems,” Antioxidants 11, no. 3 (2022): 459. 35326110 10.3390/antiox11030459 PMC8944601

[81] A. Hassaninasab , Y. Hashimoto , K. Tomita‐Yokotani , and M. Kobayashi , “Discovery of the Curcumin Metabolic Pathway Involving a Unique Enzyme in an Intestinal Microorganism,” Pnas 108, no. 16 (2011): 6615–6620. 21467222 10.1073/pnas.1016217108 PMC3080977

[82] M. R. Riaz , S. A. Rauf , R. Lupoli , and A. R. Siddiqi , Potential of Turmeric Extract and Its Fractions to Control Peach Fruit Fly (Diptera: Tephritidae), Accessed January 1, 2024 (ResearchGate, 2015), https://www.researchgate.net/publication/285899967_Potential_of_turmeric_extract_and_its_fractions_to_control_peach_fruit_fly_Diptera_Tephritidae.

[83] E. Essien , J. Newby , T. Walker , W. Setzer , and O. Ekundayo , “Chemotaxonomic Characterization and in‐Vitro Antimicrobial and Cytotoxic Activities of the Leaf Essential Oil of Curcuma Longa Grown in Southern Nigeria,” Medicines 2, no. 4 (2015): 340–349. 28930216 10.3390/medicines2040340 PMC5456208

[84] A. Nair , A. Amalraj , J. Jacob , A. B. Kunnumakkara , and S. Gopi , “Non‐Curcuminoids from Turmeric and Their Potential in Cancer Therapy and Anticancer Drug Delivery Formulations,” Biomolecules 9, no. 1 (2019): 13. 30609771 10.3390/biom9010013 PMC6358877

[85] Y. Di , A. Cao , Y. Zhang , and L. Zhang , Effects of Dietary 1,8‐Cineole Supplementation on Growth Performance, Antioxidant Capacity, Immunity, and… ResearchGate, Accessed January 1, 2024 (2022), https://www.researchgate.net/publication/363578938_Effects_of_Dietary_18‐Cineole_Supplementation_on_Growth_Performance_Antioxidant_Capacity_Immunity_and_Intestine_Health_of_Broilers. 10.3390/ani12182415 PMC9495220 36139274

[86] N. Arizmendi , S. B. Alam , K. Azyat , D. Makeiff , A. D. Befus , and M. Kulka , “The Complexity of Sesquiterpene Chemistry Dictates Its Pleiotropic Biologic Effects on Inflammation,” Molecules (Basel, Switzerland) 27, no. 8 (2022): 2450. 35458648 10.3390/molecules27082450 PMC9032002

[87] S. Ankri and D. Mirelman , “Antimicrobial Properties of Allicin from Garlic,” Microbes and Infection 1, no. 2 (1999): 125–129. 10594976 10.1016/s1286-4579(99)80003-3

[88] M. Nakamoto , K. Kunimura , J. Suzuki , and Y. Kodera , “Antimicrobial Properties of Hydrophobic Compounds in Garlic: Allicin, Vinyldithiin, Ajoene and Diallyl Polysulfides (Review),” Experimental and Therapeutic Medicine 19, no. 2 (2019): 1550–1553. 32010337 10.3892/etm.2019.8388 PMC6966194

[89] R. Leontiev , N. Hohaus , C. Jacob , M. C. H. Gruhlke , and A. J. Slusarenko , “A Comparison of the Antibacterial and Antifungal Activities of Thiosulfinate Analogues of Allicin,” Scientific Reports 8, no. 1 (2018): 6763. 29712980 10.1038/s41598-018-25154-9 PMC5928221

[90] Y. Tang , F. Li , D. Gu , W. Wang , J. Huang , and X. Jiao , “Antimicrobial Effect and the Mechanism of Diallyl Trisulfide against Campylobacter jejuni,” Antibiotics Basel 10, no. 3 (2021): 246–246. 33801353 10.3390/antibiotics10030246 PMC7999961

[91] T. H. Jakobsen , A. N. Warming , R. M. Vejborg , et al., “A Broad Range Quorum Sensing Inhibitor Working through sRNA Inhibition,” Scientific Reports 7 (2017): 9857. 28851971 10.1038/s41598-017-09886-8 PMC5575346

[92] R. Naganawa , N. Iwata , K. Ishikawa , H. Fukuda , T. Fujino , and A. Suzuki , “Inhibition of Microbial Growth by Ajoene, a Sulfur‐containing Compound Derived from Garlic,” Applied and Environmental Microbiology 62, no. 11 (1996): 4238–4242. 8900018 10.1128/aem.62.11.4238-4242.1996 PMC168248

[93] N. Rais , A. Ved , R. Ahmad , et al., “S‐Allyl‐L‐Cysteine — A Garlic Bioactive: Physicochemical Nature, Mechanism, Pharmacokinetics, and Health Promoting Activities,” Journal of Functional Foods 107 (2023): 105657.

[94] K. Yukihiro , H. Matsuura , S. Yoshida , et al., “Allixin, a Stress Compound from Garlic,” Chemical & Pharmaceutical Bulletin 37, no. 6 (1989): 1656–1658.

[95] A. P. Li , Y. H. He , S. Y. Zhang , and Y. P. Shi , “Antibacterial Activity and Action Mechanism of Flavonoids against Phytopathogenic Bacteria,” Pesticide Biochemistry and Physiology 188 (2022): 105221. 36464329 10.1016/j.pestbp.2022.105221

[96] M. Silva , V. Caro , C. Guzmán , et al., “Chapter 1 ‐ α‐Synuclein and Tau, Two Targets for Dementia,” Studies in Natural Products Chemistry 67 (2020): 1–25, https://www.sciencedirect.com/science/article/abs/pii/B9780128194836000011.

[97] F. Usai and A. Di Sotto , “trans‐Cinnamaldehyde as a Novel Candidate to Overcome Bacterial Resistance: An Overview of in Vitro Studies,” Antibiotics 12, no. 2 (2023): 254. 36830165 10.3390/antibiotics12020254 PMC9952841

[98] F. Mousavi , B. Bojko , V. Bessonneau , and J. Pawliszyn , “Cinnamaldehyde Characterization as an Antibacterial Agent toward E. coli Metabolic Profile Using 96‐Blade Solid‐Phase Microextraction Coupled to Liquid Chromatography‐Mass Spectrometry,” Journal of Proteome Research 15, no. 3 (2016): 963–975. 26811002 10.1021/acs.jproteome.5b00992

[99] Q. OuYang , X. Duan , L. Li , and N. Tao , “Cinnamaldehyde Exerts Its Antifungal Activity by Disrupting the Cell Wall Integrity of Geotrichum Citri‐aurantii,” Frontiers in Microbiology 10 (2019): 55. 30761105 10.3389/fmicb.2019.00055 PMC6364577

[100] S. H. Topa , E. A. Palombo , P. Kingshott , and L. L. Blackall , “Activity of Cinnamaldehyde on Quorum Sensing and Biofilm Susceptibility to Antibiotics in Pseudomonas aeruginosa,” Microorganisms 8, no. 3 (2020): 455. 32210139 10.3390/microorganisms8030455 PMC7143970

[101] M. Ulanowska and B. Olas , “Biological Properties and Prospects for the Application of Eugenol—A Review,” International Journal of Molecular Sciences 22, no. 7 (2021): 3671. 33916044 10.3390/ijms22073671 PMC8036490

[102] A. Marchese , R. Barbieri , E. Coppo , et al., “Antimicrobial Activity of Eugenol and Essential Oils Containing Eugenol: A Mechanistic Viewpoint,” Critical Reviews in Microbiology 43, no. 6 (2017): 668–689. 28346030 10.1080/1040841X.2017.1295225

[103] M. K. M. Elbestawy , G. M. El‐Sherbiny , and S. A. Moghannem , “Antibacterial, Antibiofilm and Anti‐Inflammatory Activities of Eugenol Clove Essential Oil against Resistant Helicobacter pylori,” Molecules (Basel, Switzerland) 28, no. 6 (2023): 2448. 36985419 10.3390/molecules28062448 PMC10058968

[104] M. Mingoia , C. Conte , A. Di Rienzo , et al., “Synthesis and Biological Evaluation of Novel Cinnamic Acid‐Based Antimicrobials,” Pharmaceuticals (Basel) 15, no. 2 (2022): 228–228. 35215340 10.3390/ph15020228 PMC8878811

[105] H. Yang , X. Tuo , L. Wang , et al., “Bioactive Procyanidins from Dietary Sources: The Relationship between Bioactivity and Polymerization Degree,” Trends in Food Science & Technology 111 (2021): 114–127.

[106] M. M. G. Mattos , S. A. Filho , G. R. Martins , et al., “Antimicrobial and Antibiofilm Properties of Procyanidins: Potential for Clinical and Biotechnological Applications,” Critical Reviews in Microbiology (2024): 1–24, 10.1080/1040841x.2024.2404509. 39301598

[107] R. M. Alkufeidy , L. Ameer Altuwijri , N. S. Aldosari , N. Alsakabi , and T. M. Dawoud , “Antimicrobial and Synergistic Properties of Green Tea Catechins against Microbial Pathogens,” Journal of King Saud University ‐ Science 36, no. 8 (2024): 103277–103277.

[108] S. S. Dahham , Y. M. Tabana , M. A. Iqbal , et al., “The Anticancer, Antioxidant and Antimicrobial Properties of the Sesquiterpene β‐Caryophyllene from the Essential Oil of Aquilaria Crassna,” Molecules (Basel, Switzerland) 20, no. 7 (2015): 11808–11829. 26132906 10.3390/molecules200711808 PMC6331975

[109] A. Scalbert , “Antimicrobial Properties of Tannins,” Phytochemistry 30, no. 12 (1991): 3875–3883.

[110] M. A. Elfaky , H. M. Okairy , H. M. Abdallah , et al., “Assessing the Antibacterial Potential of 6‐gingerol: Combined Experimental and Computational Approaches,” Saudi Pharmaceutical Journal 32, no. 5 (2024): 102041. 38558886 10.1016/j.jsps.2024.102041 PMC10981156

[111] S. Y. Ham , H. S. Kim , M. J. Jo , et al., “Combined Treatment of 6‐Gingerol Analog and Tobramycin for Inhibiting Pseudomonas aeruginosa Infections. Oglesby AG, Ed,” Microbiology Spectrum 9, no. 2 (2021): e0019221. 34704784 10.1128/Spectrum.00192-21 PMC8549756

[112] A. H. Rahmani , F. M. A. Shabrmi , and S. M. Aly , “Active Ingredients of Ginger as Potential Candidates in the Prevention and Treatment of Diseases via Modulation of Biological Activities,” International Journal of Physiology, Pathophysiology and Pharmacology 6, no. 2 (2014): 125–136. 25057339 PMC4106649

[113] T. Shelke , S. Talegaonkar , and M. Mishra , “Phytonanoparticles toward the Treatment of Diabetes,” Drug Delivery Systems for Metabolic Disorders (2022): 433–458, 10.1016/b978-0-323-99616-7.00027-x.

[114] L. Zou , Y. Y. Hu , and W. X. Chen , “Antibacterial Mechanism and Activities of Black Pepper Chloroform Extract,” Journal of Food Science and Technology 52, no. 12 (2015): 8196–8203. 26604394 10.1007/s13197-015-1914-0 PMC4648884

[115] C. P. Bravo‐Chaucanés , L. C. Chitiva , and Y. Vargas‐Casanova , “Exploring the Potential Mechanism of Action of Piperine against Candida albicans and Targeting Its Virulence Factors,” Biomolecules 13, no. 12 (2023): 1729–1729. 38136600 10.3390/biom13121729 PMC10742119

[116] S. Arora , B. Singh , S. Kumar , A. Kumar , A. Singh , and C. Singh , “Piperine Loaded Drug Delivery Systems for Improved Biomedical Applications: Current Status and Future Directions,” Health Sciences Review 9 (2023): 100138.

[117] L. T. M. Hien and D. T. A. Dao , “Antibacterial Activity of Black Pepper Essential Oil Nanoemulsion Formulated by Emulsion Phase Inversion Method,” Current Research in Nutrition and Food Science Journal 10, no. 1 (2022): 311–320.

[118] P. V. Karsha and O. B. Lakshmi , “Antibacterial Activity of Black Pepper (Piper nigrum Linn.) With Special Reference to Its Mode of Action…,” Indian Journal of Natural Products and Resources 1 (2010): 213–215.

[119] N. Wongkattiya , P. Sanguansermsri , I. H. Fraser , and D. Sanguansermsri , “Antibacterial Activity of Cuminaldehyde on Food‐borne Pathogens, the Bioactive Component of Essential Oil from Cuminum Cyminum L. collected in Thailand,” Journal of Complementary and Integrative Medicine 16, no. 4 (2019), 10.1515/jcim-2018-0195. 31129652

[120] V. Monteiro‐Neto , C. D. de Souza , L. F. Gonzaga , et al., “Cuminaldehyde Potentiates the Antimicrobial Actions of Ciprofloxacin against Staphylococcus aureus and Escherichia coli. Omri A, Ed,” PLoS ONE 15, no. 5 (2020): e0232987. 32407399 10.1371/journal.pone.0232987 PMC7224478

[121] S. Bourgou , A. Pichette , B. Marzouk , and J. Legault , “Bioactivities of Black Cumin Essential Oil and Its Main Terpenes from Tunisia,” South African Journal of Botany 76, no. 2 (2010): 210–216.

[122] A. Gueffai , D. J. Gonzalez‐Serrano , M. C. Christodoulou , et al., “Phenolics from Defatted Black Cumin Seeds (Nigella sativa L.): Ultrasound‐Assisted Extraction Optimization, Comparison, and Antioxidant Activity,” Biomolecules 12, no. 9 (2022): 1311. 36139150 10.3390/biom12091311 PMC9496517

[123] C. C. Hoch , J. Petry , L. Griesbaum , et al., “1,8‐cineole (eucalyptol): A Versatile Phytochemical with Therapeutic Applications across Multiple Diseases,” Biomedicine & Pharmacotherapy 167 (2023): 115467. 37696087 10.1016/j.biopha.2023.115467

[124] L. Li , C. Shi , Z. Yin , et al., “Antibacterial Activity of α‐terpineol May Induce Morphostructural Alterations in Escherichia coli,” Brazilian Journal of Microbiology 45, no. 4 (2015): 1409–1413. 25763048 10.1590/s1517-83822014000400035 PMC4323317

[125] X. Yang , S. Zhao , Y. Deng , et al., “Antibacterial Activity and Mechanisms of α‐terpineol against Foodborne Pathogenic Bacteria,” Applied Microbiology and Biotechnology 107, no. 21 (2023): 6641–6653. 37682300 10.1007/s00253-023-12737-4

[126] I. Noui Mehidi , A. Ait Ouazzou , W. Tachoua , and K. Hosni , “Investigating the Antimicrobial Properties of Essential Oil Constituents and Their Mode of Action,” Molecules (Basel, Switzerland) 29, no. 17 (2024): 4119–4119. 39274967 10.3390/molecules29174119 PMC11397014

[127] V. Vaičiulytė , K. Ložienė , J. Švedienė , V. Raudonienė , and A. Paškevičius , “α‐Terpinyl Acetate: Occurrence in Essential Oils Bearing Thymus Pulegioides, Phytotoxicity, and Antimicrobial Effects,” Molecules (Basel, Switzerland) 26, no. 4 (2021): 1065. 33670506 10.3390/molecules26041065 PMC7922985

[128] S. Zhou , C. Wei , C. Zhang , C. Han , N. Kuchkarova , and H. Shao , “Chemical Composition, Phytotoxic, Antimicrobial and Insecticidal Activity of the Essential Oils of Dracocephalum Integrifolium,” Toxins 11, no. 10 (2019): 598. 31614937 10.3390/toxins11100598 PMC6832822

[129] I. D. Dewijanti , W. Mangunwardoyo , and A. Dwianti , “Antimicrobial Activity of Bay Leaf (Syzygium polyanthum (wight) Walp) Extracted Using Various Solvent,” in Proc 5TH Int Symp Appl Chem 2019 , 10.1063/1.5134585, Published online 2019.

[130] Z. Shafiei , N. N. Shuhairi , N. Md Fazly Shah Yap , C. A. Harry Sibungkil , and J. Latip , “Antibacterial Activity of Myristica Fragrans against Oral Pathogens,” Evidence‐Based Complementary and Alternative Medicine ECAM 2012 (2012): 825362. 23049613 10.1155/2012/825362 PMC3434417

[131] E. F. Seneme , D. C. dos Santos , E. M. R. Silva , Y. E. M. Franco , and G. B. Longato , “Pharmacological and Therapeutic Potential of Myristicin: A Literature Review,” Molecules (Basel, Switzerland) 26, no. 19 (2021): 5914. 34641457 10.3390/molecules26195914 PMC8512857

[132] Q. P. S. Barbosa , C. A. G. da Câmara , C. S. Ramos , D. C. O. Nascimento , J. V. Lima‐Filho , and E. F. Guimarães , “Chemical Composition, Circadian Rhythm and Antibacterial Activity of Essential Oils of Piper Divaricatum: A New Source of Safrole,” Quím Nova 35 (2012): 1806–1808.

[133] M. Moghaddam , M. R. Alymanesh , L. Mehdizadeh , H. Mirzaei , and A. Ghasemi Pirbalouti , “Chemical Composition and Antibacterial Activity of Essential Oil of Ocimum Ciliatum, as a New Source of Methyl Chavicol, against Ten Phytopathogens,” Industrial Crops and Products 59 (2014): 144–148.

[134] A. Abdollahi , N. Fereydouni , H. Moradi , A. Karimivaselabadi , E. Zarenezhad , and M. Osanloo , “Nanoformulated Herbal Compounds: Enhanced Antibacterial Efficacy of Camphor and Thymol‐loaded Nanogels,” BMC Complementary Medicine and Therapies 24, no. 1 (2024): 138. 38566054 10.1186/s12906-024-04435-z PMC10985855

[135] C. Zimmermann , S. Dähn , and A. E. Wagner , “Effect of Allyl‐isothiocyanate on Survival and Antimicrobial Peptide Expression Following Oral Bacterial Infections in Drosophila Melanogaster,” Frontiers in Immunology 15 (2024): 1404086. 38803500 10.3389/fimmu.2024.1404086 PMC11128604

[136] A. Abreu , A. Borges , L. Simoes , M. Saavedra , and M. Simoes , “Antibacterial Activity of Phenyl Isothiocyanate on Escherichia coli and Staphylococcus aureus,” Medicinal Chemistry 9, no. 5 (2013): 756–761. 22974327 10.2174/1573406411309050016

[137] A. Galanty , M. Grudzińska , W. Paździora , and P. Paśko , “Erucic Acid—Both Sides of the Story: A Concise Review on Its Beneficial and Toxic Properties,” Molecules (Basel, Switzerland) 28, no. 4 (2023): 1924. 36838911 10.3390/molecules28041924 PMC9962393

[138] A. Raquel , J. Santos , M. Barbosa , et al., “Evaluation of the Antibacterial and Inhibitory Activity of NorA and MepA Efflux Pumps from Staphylococcus aureus by Diosgenin,” Life Sciences 308 (2022): 120978–120978. 36122765 10.1016/j.lfs.2022.120978

[139] A. Kar , S. Kumar Mukherjee , S. Barik , and S. Tofajjen Hossain , “Antimicrobial Activity of Trigonelline Hydrochloride Against Pseudomonas aeruginosa and Its Quorum‐Sensing Regulated Molecular Mechanisms on Biofilm Formation and Virulence,” ACS Infectious Diseases 10, no. 2 (2024): 746–762. 38232080 10.1021/acsinfecdis.3c00617

[140] S. Yadav , A. K. Singh , A. K. Agrahari , et al., “Making of Water Soluble Curcumin to Potentiate Conventional Antimicrobials by Inducing Apoptosis‐Like Phenomena among Drug‐resistant Bacteria,” Scientific Reports 10 (2020): 14204. 32848171 10.1038/s41598-020-70921-2 PMC7450046

[141] L. Zhong , Y. Lin , C. Wang , et al., “Chemical Profile, *Antimicrobial and Antioxidant Activity Assessment of the Crude Extract and Its Main Flavonoids From Tartary Buckwheat Sprouts* ,” Molecules (Basel, Switzerland) 27, no. 2 (2022): 374. 35056695 10.3390/molecules27020374 PMC8779668

[142] A. Fajdek‐Bieda , J. Pawlińska , A. Wróblewska , and A. Łuś , “Evaluation of the Antimicrobial Activity of Geraniol and Selected Geraniol Transformation Products against Gram‐Positive Bacteria,” Molecules (Basel, Switzerland) 29, no. 5 (2024): 950. 38474462 10.3390/molecules29050950 PMC10933843

[143] M. I. Naik , B. A. Fomda , E. Jaykumar , and J. A. Bhat , “Antibacterial Activity of Lemongrass (Cymbopogon citratus) Oil against some Selected Pathogenic Bacterias,” Asian Pacific Journal of Tropical Medicine 3 (2010): 535–538.

[144] J. R. Scherf , C. R. Barbosa dos Santos , T. Sampaio de Freitas , et al., “Effect of Terpinolene against the Resistant Staphylococcus aureus Strain, Carrier of the Efflux Pump QacC and β‐lactamase Gene, and Its Toxicity in the Drosophila Melanogaster Model,” Microbial Pathogenesis 149 (2020): 104528. 33002597 10.1016/j.micpath.2020.104528

[145] L. Dedieu , J. M. Brunel , V. Lorenzi , A. Muselli , L. Berti , and J. M. Bolla , “Antibacterial Mode of Action of the Daucus Carota Essential Oil Active Compounds against Campylobacter Jejuni and Efflux‐Mediated Drug Resistance in Gram‐Negative Bacteria,” Molecules (Basel, Switzerland) 25, no. 22 (2020): 5448. 33233754 10.3390/molecules25225448 PMC7699865

[146] L. Tao , Y. Liang , Z. Xia , et al., “Antibacterial Activities of Oregano Essential Oils and Their Active Components,” Frontiers in Pharmacology 16 (2025): 1579283. 40264663 10.3389/fphar.2025.1579283 PMC12011810

[147] A. Hajibonabi , M. Yekani , S. Sharifi , J. S. Nahad , S. M. Dizaj , and M. Y. Memar , “Antimicrobial Activity of Nanoformulations of Carvacrol and Thymol: New Trend and Applications,” OpenNano 13 (2023): 100170.

[148] M. Aydin , N. Unusan , E. Sumlu , and E. N. Korucu , “Rosmarinic Acid Exhibits Antifungal and Antibiofilm Activities Against Candida albicans: Insights into Gene Expression and Morphological Changes,” Journal of Fungi 10 (2024): 751. 39590670 10.3390/jof10110751 PMC11595412

[149] N. Ghadimi , L. Asadpour , and M. Mokhtary , “Enhanced Antimicrobial, Anti‐biofilm, and Efflux Pump Inhibitory Effects of Ursolic Acid‐conjugated Magnetic Nanoparticles against Clinical Isolates of Multidrug‐resistant Pseudomonas aeruginosa,” Microbial Pathogenesis 199 (2025): 107241. 39716652 10.1016/j.micpath.2024.107241

[150] S. Nabavi , A. Di Lorenzo , M. Izadi , E. Sobarzo‐Sánchez , M. Daglia , and S. Nabavi , “Antibacterial Effects of Cinnamon: From Farm to Food, Cosmetic and Pharmaceutical Industries,” Nutrients 7, no. 9 (2015): 7729–7748. 26378575 10.3390/nu7095359 PMC4586554

[151] Y. Hussain , W. Alam , H. Ullah , et al., “Antimicrobial Potential of Curcumin: Therapeutic Potential and Challenges to Clinical Applications,” Antibiotics 11, no. 3 (2022): 322. 35326785 10.3390/antibiotics11030322 PMC8944843

[152] Z. Al Noman , T. Tabassum Anika , S. Sachi , et al., “Evaluation of Antibacterial Efficacy of Garlic (Allium sativum) and Ginger (Zingiber officinale) Crude Extract against Multidrug‐resistant (MDR) Poultry Pathogen,” Journal of Advanced Veterinary and Animal Research 10, no. 2 (2023): 151–151. 37534079 10.5455/javar.2023.j664 PMC10390675

[153] J. Bai , J. Li , Z. Chen , et al., “Antibacterial Activity and Mechanism of Clove Essential Oil against Foodborne Pathogens,” LWT 173 (2023): 114249. 10.1089/fpd.2022.0084 37010405

[154] X. Wang , Y. Shen , K. Thakur , et al., “Antibacterial Activity and Mechanism of Ginger Essential Oil against Escherichia coli and Staphylococcus aureus,” Molecules (Basel, Switzerland) 25, no. 17 (2020): 3955. 32872604 10.3390/molecules25173955 PMC7504760

[155] J. Zhang , K. P. Ye , X. Zhang , D. D. Pan , Y. Y. Sun , and J. X. Cao , “Antibacterial Activity and Mechanism of Action of Black Pepper Essential Oil on Meat‐Borne Escherichia coli,” Frontiers in Microbiology 7 (2017): 2094. 28101081 10.3389/fmicb.2016.02094 PMC5209337

[156] E. A. Rodrigues dos Santos , L. Ereno Tadielo , J. Arruda Schmiedt , et al., “Inhibitory Effects of Piperine and Black Pepper Essential Oil on Multispecies Biofilm Formation by Listeria monocytogenes, Salmonella Typhimurium, and Pseudomonas aeruginosa,” LWT 182 (2023): 114851. 10.1007/s42770-023-01075-2 PMC10689688 37668830

[157] A. Abbaszadegan , A. Gholami , Y. Ghahramani , et al., “Antimicrobial and Cytotoxic Activity of Cuminum Cyminum as an Intracanal Medicament Compared to Chlorhexidine Gel,” Iranian Endodontic Journal 11, no. 1 (2016): 44–50. 26843877 10.7508/iej.2016.01.009 PMC4731533

[158] J. Wanner , S. Bail , L. Jirovetz , et al., “Chemical Composition and Antimicrobial Activity of Cumin Oil (Cuminum Cyminum, Apiaceae),” Natural Products Communications 5, no. 9 (2010): 1934578×1000500. 20922990

[159] S. M. da Silveira , F. B. Luciano , N. Fronza , A. Cunha , G. N. Scheuermann , and C. R. W. Vieira , “Chemical Composition and Antibacterial Activity of Laurus Nobilis Essential Oil towards Foodborne Pathogens and Its Application in Fresh Tuscan Sausage Stored at 7°C,” LWT ‐ Food Science and Technology 59, no. 1 (2014): 86–93.

[160] K. A. Panggabean , H. Rusmarilin , and D. Suryanto , “The Utilization of Nutmeg Seed (Myristica fragrans Houtt) Extract as an Antimicrobial on Tempeh Sausage,” IOP Conference Series: Earth and Environmental Science 260 (2019): 012087.

[161] C. A. Semeniuc , C. R. Pop , and A. M. Rotar , “Antibacterial Activity and Interactions of Plant Essential Oil Combinations against Gram‐positive and Gram‐negative Bacteria,” Journal of Food and Drug Analysis 25, no. 2 (2017): 403–408. 28911683 10.1016/j.jfda.2016.06.002 PMC9332530

[162] B. Schweitzer , V. L. Balázs , S. Molnár , et al., “Antibacterial Effect of Lemongrass (Cymbopogon citratus) against the Aetiological Agents of Pitted Keratolyis,” Molecules (Basel, Switzerland) 27, no. 4 (2022): 1423. 35209211 10.3390/molecules27041423 PMC8878996

[163] M. Walasek‐Janusz , A. Grzegorczyk , A. Malm , R. Nurzyńska‐Wierdak , and D. Zalewski , “Chemical Composition, and Antioxidant and Antimicrobial Activity of Oregano Essential Oil,” Molecules (Basel, Switzerland) 29 (2024): 435–435. 38257351 10.3390/molecules29020435 PMC10818459

[164] D. Gwiazdowska , A. Waśkiewicz , K. Juś , et al., “Antimicrobial and Antibiofilm Activity of *Origanum vulgare* Extracts Obtained by Supercritical Fluid Extraction Under Various Extraction Conditions,” Molecules (Basel, Switzerland) 29 (2024): 5823. 39769912 10.3390/molecules29245823 PMC11676637

[165] J. W. He , M. Hadidi , S. Yang , M. R. Khan , W. Zhang , and X. Cong , “Natural Food Preservation with Ginger Essential Oil: Biological Properties and Delivery Systems,” Food Research International 173 (2023): 113221–113221. 37803539 10.1016/j.foodres.2023.113221

[166] S. Chatterjee , P. Paul , P. Chakraborty , et al., “Cuminaldehyde Exhibits Potential Antibiofilm Activity against Pseudomonas aeruginosa Involving Reactive Oxygen Species (ROS) Accumulation: A Way Forward towards Sustainable Biofilm Management,” 3 Biotech 11 (2021): 485. 10.1007/s13205-021-03013-1 PMC8558144 34790509

[167] R. Irkin and O. K. Esmer , “Novel Food Packaging Systems with Natural Antimicrobial Agents,” Journal of Food Science and Technology 52 (2015): 6095–6111. 26396358 10.1007/s13197-015-1780-9 PMC4573172

[168] J. Ahmed , E. Altun , M. O. Aydogdu , et al., “Anti‐fungal Bandages Containing Cinnamon Extract,” International Wound Journal 16, no. 3 (2019): 730–736. 30767437 10.1111/iwj.13090 PMC6849878

[169] Y. Dai , M. Gultekinoglu , C. Bayram , H. B. De Silva , and M. Edirisinghe , “Antibacterial Properties of Natural Cinnamon‐alginate Fibrous Patches Produced by Modified Nozzle‐pressurized Spinning,” MedComm 5, no. 9 (2024): e731. 39263606 10.1002/mco2.731 PMC11387719

[170] C. Kotlowski , P. Aspermair , H. Ullah Khan , et al., “Electronic Biosensing with Flexible Organic Transistor Devices,” Flexible and Printed Electronics 3, no. 3 (2018): 034003–034003.

[171] J. Wang , X. Zhou , W. Li , X. Deng , Y. Deng , and X. Niu , “Curcumin Protects Mice from Staphylococcus aureus Pneumonia by Interfering with the Self‐assembly Process of α‐hemolysin,” Scientific Reports 6 (2016): 28254. 27345357 10.1038/srep28254 PMC4921848

[172] R. Wang , L. van Dorp , L. P. Shaw , et al., “The Global Distribution and Spread of the Mobilized Colistin Resistance Gene Mcr‐1,” Nature Communications 9 (2018): 1179. 10.1038/s41467-018-03205-z PMC5862964 29563494

[173] G. Hellen , J. A. Santos , and M. Soares , “In Vitro and in Vivo Antibacterial Activity Assays of Carvacrol: A Candidate for Development of Innovative Treatments against KPC‐producing Klebsiella pneumoniae,” PLoS ONE 16 (2021): e0246003. 33617571 10.1371/journal.pone.0246003 PMC7899316

[174] W. A. Pereira , C. D. S. Pereira , R. G. Assunção , et al., “New Insights into the Antimicrobial Action of Cinnamaldehyde towards Escherichia coli and Its Effects on Intestinal Colonization of Mice,” Biomolecules 11 (2021): 302. 33670478 10.3390/biom11020302 PMC7922552

[175] I. F. S. Figueiredo , L. G. Araújo , R. G. Assunção , et al., “Cinnamaldehyde Increases the Survival of Mice Submitted to Sepsis Induced by Extraintestinal Pathogenic Escherichia coli,” Antibiotics 11 (2022): 364. 35326827 10.3390/antibiotics11030364 PMC8944619

[176] J. Li , T. Lu , Y. Chu , et al., “Cinnamaldehyde Targets SarA to Enhance β‐lactam Antibiotic Activity against Methicillin‐resistant *Staphylococcus aureus* ,” mLife 3 (2024): 291–306. 38948140 10.1002/mlf2.12121 PMC11211666

[177] H. B. van der Worp , D. W. Howells , E. S. Sena , et al., “Can Animal Models of Disease Reliably Inform Human Studies?,” Plos Medicine 7 (2010): e1000245. 20361020 10.1371/journal.pmed.1000245 PMC2846855

[178] C. Bitwell , S. S. Indra , C. Luke , and M. K. Kakoma , “A Review of Modern and Conventional Extraction Techniques and Their Applications for Extracting Phytochemicals from Plants,” Scientific African 19 (2023): e01585.

[179] A. S. Caballero Galván , C. E. O. Alzate , and C. Á. Cardona Álzate , Economic Assessment of Polyphenolic Compounds Production at Different Purities and Applications (2017), https://www.researchgate.net/publication/330369288_Economic_assessment_of_polyphenolic_compounds_production_at_different_purities_and_applications.

[180] A. E. Atabani , A. S. Silitonga , H. C. Ong , et al., “Non‐edible Vegetable Oils: A Critical Evaluation of Oil Extraction, Fatty Acid Compositions, Biodiesel Production, Characteristics, Engine Performance and Emissions Production,” Renewable and Sustainable Energy Reviews 18 (2013): 211–245.

[181] P. Kapadia , A. S. Newell , J. Cunningham , M. R. Roberts , and J. G. Hardy , “Extraction of High‐Value Chemicals from Plants for Technical and Medical Applications,” International Journal of Molecular Sciences 23, no. 18 (2022): 10334. 36142238 10.3390/ijms231810334 PMC9499410

[182] Shahid‐ul‐Islam , M. Shahid , and F. Mohammad , “Perspectives for Natural Product Based Agents Derived from Industrial Plants in Textile Applications—A Review,” Journal of Cleaner Production 57 (2013): 2–18.

[183] P. Tongnuanchan and S. Benjakul , “Essential Oils: Extraction, Bioactivities, and Their Uses for Food Preservation,” Journal of Food Science 79, no. 7 (2014): R1231–R1249. 24888440 10.1111/1750-3841.12492

[184] A. Rafiq , B. Manzoor , M. A. Nayeem , A. Jabeen , and Q. A. Amin , Extraction of Essential Oils, Published online (Elsevier EBooks, 2024), 279–298, 10.1016/b978-0-12-819516-1.00005-3.

[185] A. Alves Prestes , M. Helena , C. Vieira Helm , and A. G. Cruz , “Elane Schwinden Prudencio. The Use of Cold Pressing Technique Associated with Emerging Nonthermal Technologies in the Preservation of Bioactive Compounds in Tropical Fruit Juices: An Overview,” Current Opinion in Food Science 51 (2023): 101005–101005.

[186] M. Gonzalez‐Miquel and J. Esteban , Novel Solvents for Biotechnology Applications, Published online (2019), 790–806, 10.1016/b978-0-444-64046-8.00459-6.

[187] I. S. Pratama , Y. Putra , R. Pangestuti , S. K. Kim , and E. A. Siahaan , “Bioactive Peptides‐derived from Marine by‐products: Development, Health Benefits and Potential Application in Biomedicine,” Fisheries and Aquatic Sciences 25, no. 7 (2022): 357–379.

[188] C. J. Qin , “Spices and Flavoring (Flavouring) Crops | Properties and Analysis,” in Elsevier EBooks, Published online January (2003), 5491–5501, 10.1016/b0-12-227055-x/01126-3.

[189] V. I. Sousa , J. F. Parente , J. F. Marques , M. A. Forte , and C. J. Tavares , “Microencapsulation of Essential Oils: A Review,” Polymers 14, no. 9 (2022): 1730. 35566899 10.3390/polym14091730 PMC9099681

[190] H. M. A. AlSheikh , I. Sultan , V. Kumar , et al., “Plant‐Based Phytochemicals as Possible Alternative to Antibiotics in Combating Bacterial Drug Resistance,” Antibiotics 9, no. 8 (2020): 480. 32759771 10.3390/antibiotics9080480 PMC7460449

[191] A. Shariati , M. Noei , M. Askarinia , A. Khoshbayan , A. Farahani , and Z. Chegini , “Inhibitory Effect of Natural Compounds on Quorum Sensing System in Pseudomonas aeruginosa: A Helpful Promise for Managing Biofilm Community,” Frontiers in Pharmacology 15 (2024): 1350391. 38628638 10.3389/fphar.2024.1350391 PMC11019022

[192] A. Avakh , G. Grant , M. J. Cheesman , T. Kalkundri , and S. Hall , “The Art of War with Pseudomonas aeruginosa: Targeting Mex Efflux Pumps Directly to Strategically Enhance Antipseudomonal Drug Efficacy,” Antibiotics 12 (2023): 1304–1304. 37627724 10.3390/antibiotics12081304 PMC10451789

[193] N. S. Sundaramoorthy , A. Sivasubramanian , and S. Nagarajan , “Simultaneous Inhibition of MarR by Salicylate and Efflux Pumps by Curcumin Sensitizes Colistin Resistant Clinical Isolates of Enterobacteriaceae,” Microbial Pathogenesis 148 (2020): 104445. 32814143 10.1016/j.micpath.2020.104445

[194] M. Ghasemi , M. H. Nowroozzadeh , F. Ghorat , A. Iraji , and M. H. Hashempur , “Piperine and Its Nanoformulations: A Mechanistic Review of Their Anti‐cancer Activities,” Biomedicine and Pharmacotherapy 187 (2025): 118075. 40273688 10.1016/j.biopha.2025.118075

[195] N. R. Khanna and V. Gerriets , Beta Lactamase Inhibitors, Published online September (2022), https://www.ncbi.nlm.nih.gov/books/NBK557592/. 32491524

[196] K. S. fang , X. J , C. Y. tao , P. X. xian , H. Li , and B. Peng , “Exogenous Pyruvate Promotes Gentamicin Uptake to Kill Antibiotic‐resistant Vibrio Alginolyticus,” International Journal of Antimicrobial Agents 63 (2024): 107036–107036. 37981076 10.1016/j.ijantimicag.2023.107036

[197] B. R. Wagle , A. Upadhyay , I. Upadhyaya , et al., “Trans‐Cinnamaldehyde, Eugenol and Carvacrol Reduce Campylobacter Jejuni Biofilms and Modulate Expression of Select Genes and Proteins,” Frontiers in Microbiology 10 (2019): 1837. 31456771 10.3389/fmicb.2019.01837 PMC6698798

[198] T. H. Jakobsen , M. van Gennip , R. K. Phipps , et al., “Ajoene, a Sulfur‐Rich Molecule from Garlic, Inhibits Genes Controlled by Quorum Sensing,” Antimicrobial Agents and Chemotherapy 56 (2012): 2314–2325. 22314537 10.1128/AAC.05919-11 PMC3346669

[199] K. Farhadi , E. Rajabi , H. A. Varpaei , M. Iranzadasl , S. Khodaparast , and M. Salehi , “Thymol and Carvacrol against Klebsiella: Anti‐bacterial, Anti‐biofilm, and Synergistic Activities—a Systematic Review,” Frontiers in Pharmacology 15 (2024): 1487083. 39512827 10.3389/fphar.2024.1487083 PMC11540684

[200] R. Bernini and F. Velotti , “Natural Polyphenols as Immunomodulators to Rescue Immune Response Homeostasis: Quercetin as a Research Model against Severe COVID‐19,” Molecules (Basel, Switzerland) 26 (2021): 5803. 34641348 10.3390/molecules26195803 PMC8510228

[201] H. Dong , G. Song , Z. Wang , X. Wu , Q. Wang , and Y. H. Wang , “Kaempferol as a Multifaceted Immunomodulator: Implications for Inflammation, Autoimmunity, and Cancer,” Frontiers in Immunology 16 (2025): 1671519. 40918131 10.3389/fimmu.2025.1671519 PMC12408509

[202] R. E. Glover , A. C. Singer , A. P. Roberts , and C. Kirchhelle , “NIMble Innovation—a Networked Model for Public Antibiotic Trials,” The Lancet Microbe 2, no. 11 (2021): e637–e644. 35544083 10.1016/S2666-5247(21)00182-8

[203] E. Zhang , “Turmeric‐Induced Liver Injury in a Patient with Concurrent Semaglutide Use,” American Journal of Hospital Medicine 8 (2024), 10.24150/ajhm/2023.012.

[204] D. Soleimani , Z. Paknahad , and M. H. Rouhani , “Therapeutic Effects of Garlic on Hepatic Steatosis in Nonalcoholic Fatty Liver Disease Patients: A Randomized Clinical Trial,” Diabetes, Metabolic Syndrome and Obesity: Targets and Therapy 13 (2020): 2389–2397. 32753923 10.2147/DMSO.S254555 PMC7354004

[205] N. Iwata , M. Kainuma , D. Kobayashi , et al., “The Relation between Hepatotoxicity and the Total Coumarin Intake from Traditional Japanese Medicines Containing Cinnamon Bark,” Frontiers in Pharmacology 7 (2016): 174. 27378929 10.3389/fphar.2016.00174 PMC4913087

[206] LiverTox Eugenol (Clove Oil), Published online 2012, https://www.ncbi.nlm.nih.gov/books/NBK551727/.

[207] M. Nikkhah Bodagh , I. Maleki , and A. Hekmatdoost , “Ginger in Gastrointestinal Disorders: A Systematic Review of Clinical Trials,” Food Science & Nutrition 7 (2019): 96–108. 30680163 10.1002/fsn3.807 PMC6341159

[208] R. Ziegenhagen , K. Heimberg , A. Lampen , and K. I. Hirsch‐Ernst , “Safety Aspects of the Use of Isolated Piperine Ingested as a Bolus,” Foods 10 (2021): 2121. 34574230 10.3390/foods10092121 PMC8467119

[209] H. Çelik and E. Kumaş , “Natural Aldehydes on Health Effects,” Oriental Journal of Chemistry 41 (2025), https://www.orientjchem.org/vol41no4/natural‐aldehydes‐on‐health‐effects/.

[210] V. Bampidis , G. Azimonti , M. de Lourdes Bastos , et al., “Safety and Efficacy of an Essential Oil from Elettaria Cardamomum (L.) Maton When Used as a Sensory Additive in Feed for all Animal Species,” EFSA Journal 17 (2019): e05721. 32626343 10.2903/j.efsa.2019.5721 PMC7009127

[211] H. J. Sipe , O. M. Lardinois , and R. P. Mason , “Free Radical Metabolism of Methyleugenol and Related Compounds,” Chemical Research in Toxicology 27 (2014): 483–489. 24564854 10.1021/tx400256b PMC4002132

[212] K. Usui , E. Kubota , H. Kobayashi , et al., “Detection of Major Psychoactive Compounds (safrole, myristicin, and elemicin) of Nutmeg in human Serum via GC‐MS/MS Using MonoSpin® Extraction: Application in a Nutmeg Poisoning Case,” Journal of Pharmaceutical and Biomedical Analysis 234 (2023): 115565. 37453146 10.1016/j.jpba.2023.115565

[213] A. Eisenreich , L. Wittek , M. Sagmeister , et al., “Comparative Analysis of Estragole, Methyleugenol, Myristicin, and Elemicin Regarding Micronucleus Formation in V79 Cells,” Molecules (Basel, Switzerland) 30 (2025): 806–806. 40005118 10.3390/molecules30040806 PMC11858557

[214] W. Alibrahem , H. Nguyen , N. K. Helu , et al., “Health Benefits, Applications, and Analytical Methods of Freshly Produced Allyl Isothiocyanate,” Foods 14 (2025): 579–579. 40002023 10.3390/foods14040579 PMC11853810

[215] M. Shabil , G. Bushi , P. K. Bodige , et al., “Effect of Fenugreek on Hyperglycemia: A Systematic Review and Meta‐Analysis,” Medicina 59 (2023): 248. 36837450 10.3390/medicina59020248 PMC9962665

[216] A. Sindle and K. Martin , “Essential Oils—Natural Products Not Necessarily Safe,” International Journal of Women's Dermatology 7 (2020): 304–308. 10.1016/j.ijwd.2020.10.013 PMC8243157 34222588

[217] M. Llana‐Ruiz‐Cabello , D. Gutiérrez‐Praena , S. Pichardo , et al., “Cytotoxicity and Morphological Effects Induced by Carvacrol and Thymol on the human Cell Line Caco‐2,” Food and Chemical Toxicology 64 (2014): 281–290. 24326232 10.1016/j.fct.2013.12.005

[218] S. C. Gupta , S. Patchva , and B. B. Aggarwal , “Therapeutic Roles of Curcumin: Lessons Learned from Clinical Trials,” The Aaps Journal [Electronic Resource] 15, no. 1 (2012): 195–218. 23143785 10.1208/s12248-012-9432-8 PMC3535097

[219] G. Hartnoll , D. Moore , and D. Douek , “Near Fatal Ingestion of Oil of Cloves,” Archives of Disease in Childhood 69, no. 3 (1993): 392–393. 8215554 10.1136/adc.69.3.392 PMC1029532

[220] J. E. Ehrenpreis , C. DesLauriers , P. Lank , P. K. Armstrong , and J. B. Leikin , “Nutmeg Poisonings: A Retrospective Review of 10 Years Experience from the Illinois Poison Center, 2001–2011,” Journal of Medical Toxicology 10, no. 2 (2014): 148–151. 24452991 10.1007/s13181-013-0379-7 PMC4057546

[221] T. Yamada , N. Katsutani , T. Maruyama , et al., “Combined Risk Assessment of Food‐derived Coumarin with in Silico Approaches,” Food Safety 10, no. 3 (2022): 73–82. 36237397 10.14252/foodsafetyfscj.D-21-00015 PMC9509535

[222] A. Vidalis , O. Dumoulin , M. Godbole , and C. C. Proenca , “The Role and Value of Real‐world Evidence in Health Technology Decision‐making in France, Germany, Italy, Spain, and the UK: Insights on External Control Arms,” International Journal of Technology Assessment in Health Care 41 (2025): e25. 40260460 10.1017/S0266462324004720 PMC12019763

[223] D. H. Ryu , J. Y. Cho , S. H. Yang , and H. Y. Kim , “Effects of Harvest Timing on Phytochemical Composition in Lamiaceae Plants under an Environment‐Controlled System,” Antioxidants 12 (2023): 1909–1909. 38001762 10.3390/antiox12111909 PMC10669742

[224] S. Govindaraghavan and N. J. Sucher , “Quality Assessment of Medicinal Herbs and Their Extracts: Criteria and Prerequisites for Consistent Safety and Efficacy of Herbal Medicines,” Epilepsy & Behavior 52 (2015): 363–371. 25899015 10.1016/j.yebeh.2015.03.004

[225] R. Tabanelli , S. Brogi , and V. Calderone , “Improving Curcumin Bioavailability: Current Strategies and Future Perspectives,” Pharmaceutics 13 (2021): 1715. 34684008 10.3390/pharmaceutics13101715 PMC8540263

[226] Z. Goktas , Y. Zu , M. Abbasi , et al., “Recent Advances in Nanoencapsulation of Phytochemicals to Combat Obesity and Its Comorbidities,” Journal of Agricultural and Food Chemistry 68 (2020): 8119–8131. 32633507 10.1021/acs.jafc.0c00131 PMC8507418

[227] M. Gera , N. Sharma , M. Ghosh , et al., “Nanoformulations of Curcumin: An Emerging Paradigm for Improved Remedial Application,” Oncotarget 8 (2017): 66680–66698. 29029547 10.18632/oncotarget.19164 PMC5630447

[228] D. Yang , H. Eun , and C. Pricilia , “Metabolic Engineering and Synthetic Biology Approaches for the Heterologous Production of Aromatic Polyketides,” International Journal of Molecular Sciences 24 (2023): 8923–8923. 37240269 10.3390/ijms24108923 PMC10219323

[229] A. Gangwal and A. Lavecchia , “Artificial Intelligence in Natural Product Drug Discovery: Current Applications and Future Perspectives,” Journal of Medicinal Chemistry 68, no. 4 (2025): 3948–3969, Published online February 2025. 39916476 10.1021/acs.jmedchem.4c01257 PMC11874025

[230] M. Ghazi Vakili , C. Gorgulla , J. Snider , et al., “Quantum‐computing‐enhanced Algorithm Unveils Potential KRAS Inhibitors,” Nature Biotechnology 43, no. 12 (2025): 1954–1959, Published online January 2025. 10.1038/s41587-024-02526-3 PMC12700792 39843581

[231] S. Salm , J. Rutz , R. A. van Blaheta , and B. E. Bachmeier , “Current state of Research on the Clinical Benefits of Herbal Medicines for Non‐life‐threatening Ailments,” Frontiers in Pharmacology 14 (2023): 1234701. 37841934 10.3389/fphar.2023.1234701 PMC10569491

